# Bioinspired Diversification Approach Toward the Total Synthesis of Lycodine-Type Alkaloids

**DOI:** 10.1021/jacs.1c00457

**Published:** 2021-03-17

**Authors:** Hannah M. S. Haley, Stefan E. Payer, Sven M. Papidocha, Simon Clemens, Jonathan Nyenhuis, Richmond Sarpong

**Affiliations:** Department of Chemistry, University of California, Berkeley, California 94720, United States

## Abstract

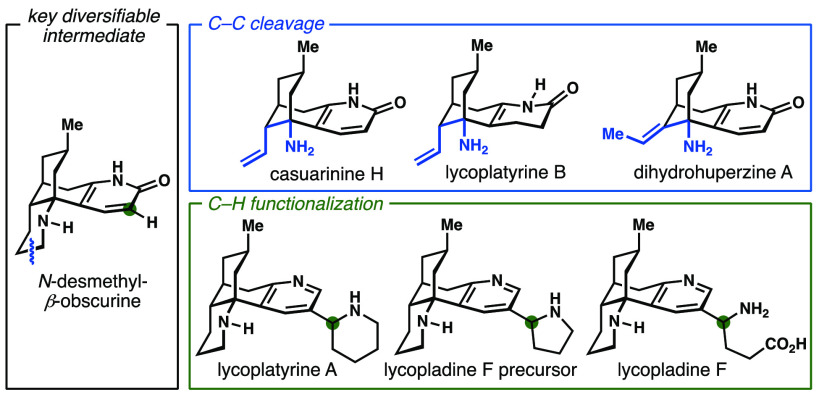

Nitrogen heterocycles (azacycles) are common structural motifs in numerous pharmaceuticals, agrochemicals, and natural products. Many powerful methods have been developed and continue to be advanced for the selective installation and modification of nitrogen heterocycles through C–H functionalization and C–C cleavage approaches, revealing new strategies for the synthesis of targets containing these structural entities. Here, we report the first total syntheses of the lycodine-type *Lycopodium* alkaloids casuarinine H, lycoplatyrine B, lycoplatyrine A, and lycopladine F as well as the total synthesis of 8,15-dihydrohuperzine A through bioinspired late-stage diversification of a readily accessible common precursor, *N*-desmethyl-β-obscurine. Key steps in the syntheses include oxidative C–C bond cleavage of a piperidine ring in the core structure of the obscurine intermediate and site-selective C–H borylation of a pyridine nucleus to enable cross-coupling reactions.

## Introduction

**Figure 1:**
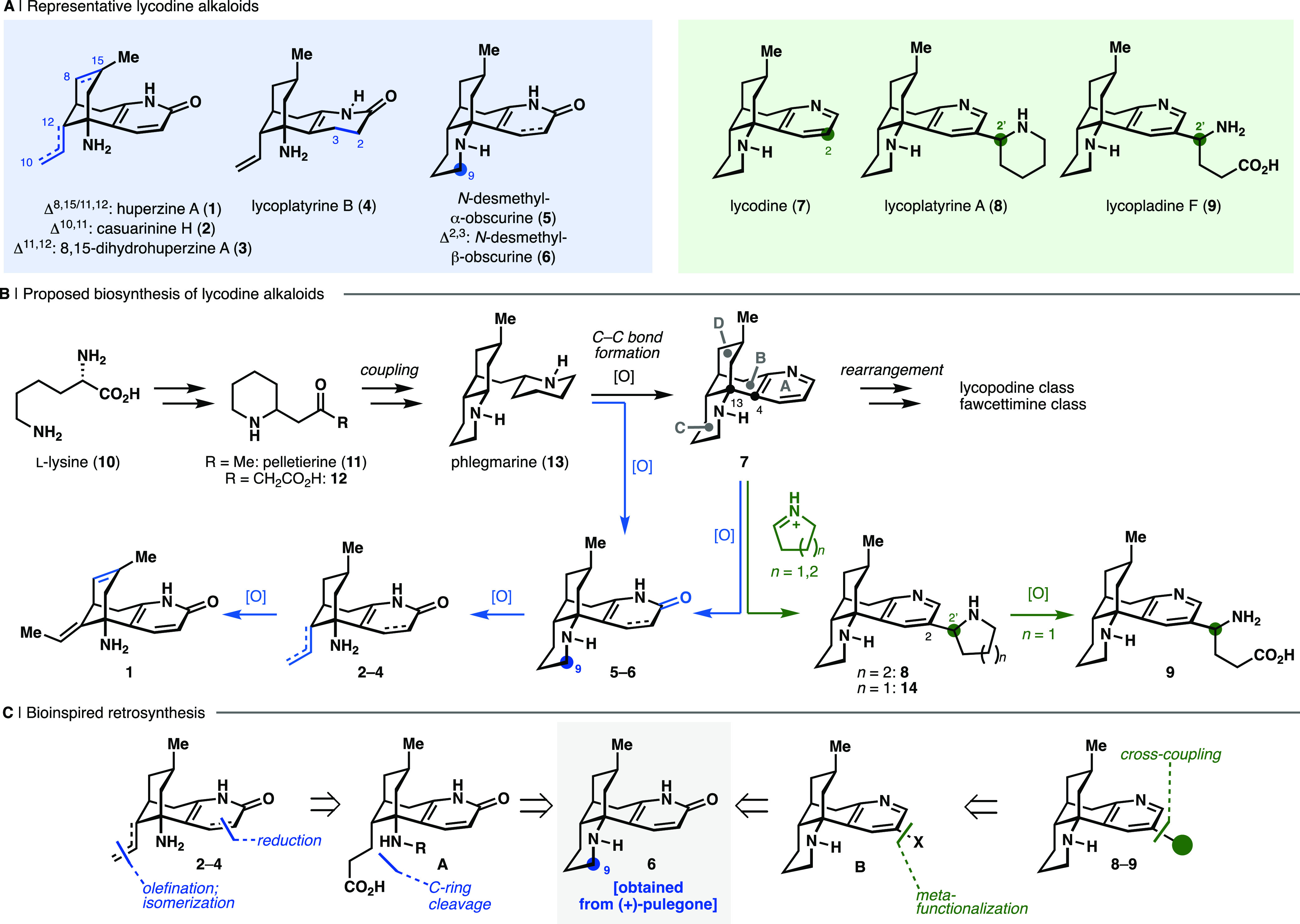
Bioinspired plans for the synthesis of lycodine alkaloids.

The *Lycopodium* alkaloids are a diverse group of natural products found in plants of the widely distributed *Lycopodium* genus, commonly known as clubmosses.^1,2^ Since the isolation of the first of these alkaloids, lycopodine, in 1881,^3^ a wealth of biosynthetically related alkaloids have also been isolated and characterized. These natural products are organized into four main classes (lycodine, lycopodine, fawcettimine, and a miscellaneous class) on the basis of their distinct carbon backbones, which arise as a consequence of C–C bond formation and rearrangement events during their putative biosyntheses.^2^ Many *Lycopodium* alkaloids possess intriguing and complex molecular architectures, and also display promising bioactivity profiles. The archetypical lycodine alkaloid huperzine A (**1**, Figure 1a), for example, is a potent and selective acetylcholinesterase (AChE) inhibitor and also demonstrates noncholinergic neuroprotective effects.^4−6^ This bioactivity is of interest for the symptomatic treatment of Alzheimer’s disease and other neurodegenerative disorders.^2,4−6^ The combination of interesting structural features and noteworthy bioactivity continue to drive synthetic studies toward *Lycopodium* alkaloids and their analogues.^7−12^

Synthetic strategies that enable late-stage structural modification and diversification of a common advanced intermediate can provide versatility that facilitates efficient access to a range of products that might otherwise each require significant synthetic investment. A rapidly growing catalog of C–H bond functionalization technologies has powerfully expanded the processes available for such structural alterations, typically elaborating around the periphery of a molecule.^13^ Alternatively, C–C bond cleavage and functionalization strategies represent a key complementary approach which can be applied to remodel not only the periphery but also the core carbon skeleton of organic compounds.^14^ Although C–C cleavage tactics typically result in a decrease in molecular complexity—in contrast to Corey’s retrosynthetic paradigm^15^—they can lead to the identification of new retrosynthetic disconnections. In turn, such methods could enable rapid access to a diverse range of natural products or bioactive agents from a single compound, which, albeit more structurally complex, is easily obtained through chemical synthesis, biosynthesis, or synthetic biology.

The ubiquity of nitrogen heterocycles in pharmaceuticals,^16^ agrochemicals, and alkaloids^17^ render them attractive structural motifs for diversification to efficiently access underexplored chemical space.^18^ Therefore, a variety of methods for both the introduction and selective functionalization of azacycles continue to be reported.^19−21^ Inspired by these contributions, we envisioned nitrogen heterocycles as versatile synthetic handles that would enable the expedient preparation of a collection of lycodine-type alkaloids (**2**–**4**, **8**, **9**, Figure 1a) from a common, readily prepared, precursor through a series of programmed oxidation and C–C bond cleavage events in analogy to their biosynthesis.^2,22^

Although the complete biosynthetic pathways to the *Lycopodium* alkaloids remain to be fully elucidated,^23^ biochemical studies have suggested that these compounds derive from phlegmarine (**13**), which arises from the coupling of pelletierine (**11**) and 4-(2-piperidyl) acetoacetate (**12**), both of which originate from l-lysine (**10**, Figure 1b).^2^

Subsequent closure of ring B through bond formation between C13 and C4 furnishes the characteristic [3.3.1]-bicyclic scaffold of the lycodine class. A series of oxidative modifications, which include oxidation of the A-ring to the corresponding pyridone (e.g., in *N*-desmethyl-β-obscurine, **6**) or pyridine (e.g., in lycodine, **7**), C-ring cleavage, and excision of C9 further diversifies the parent scaffold, yielding a range of alkaloids including **1**–**6** (Figure 1b, blue arrows).

On the basis of these presumed biosynthetic events, we envisioned a retrosynthesis (Figure 1c) in which 8,15-dihydrohuperzine A (**3**)^24^ could arise from casuarinine H (**2**)^25^ through olefin isomerization, whereas lycoplatyrine B (**4**)^26^ could be accessed from **2** through semireduction of the pyridone. Casuarinine H (**2**) was traced back to functionalized tricyclic intermediate **A** through decarboxyolefination. In turn, **A** could be formed from the readily accessible key precursor *N*-desmethyl-β-obscurine (**6**) through oxidative functionalization and cleavage of the C9–N bond.

Another small set of structurally unique lycodine alkaloids bearing substitution at the C2 position of the pyridine A-ring (e.g., lycoplatyrine A,^26^**8**, and lycopladine F,^27^**9**) is proposed to arise biosynthetically through electrophilic substitution on lycodine (**7**) or the corresponding dihydropyridine by a Δ^1^-piperidinium or Δ^1^-pyrrolinium cation (or the corresponding imines; Figure 1b, green arrows).^26,27^ Subsequent oxidative cleavage of the pyrrolidine ring in **14** is suggested to provide lycopladine F (**9**), analogous to the oxidative ring cleavage pathway that leads to metabolic products of nicotine.^28^ Overall, we envisioned lycoplatyrine A (**8**) and lycopladine F (**9**) could be accessed through cross-coupling of appropriate C(*sp*^3^) nucleophiles with a functionalized lycodine analog (**B**), which again would be prepared from the key obscurine scaffold **6**. The required deoxygenation of precursor **6** and site-selective functionalization at C2 would rely upon precedent demonstrated by our laboratories in the total synthesis of the dimeric lycodine alkaloids complanadine A and B.^29,30^

## Results and Discussion

### Preparation of the Key Diversifiable Precursor

Our investigations commenced with the development of a robust synthesis of *N*-desmethyl-β-obscurine (**6**), the late-stage common intermediate for the synthesis of all of the alkaloids described here. A convergent route featuring a diastereoselective formal (3 + 3)-cycloaddition to form the three contiguous stereocenters and two C–C bonds in ring B of **6**(31) was adapted from literature protocols by Schuster,^32^ Caine,^33^ Dake,^34^ and Jung^35^ as well as our own previous studies.^29^

**Scheme 1:**
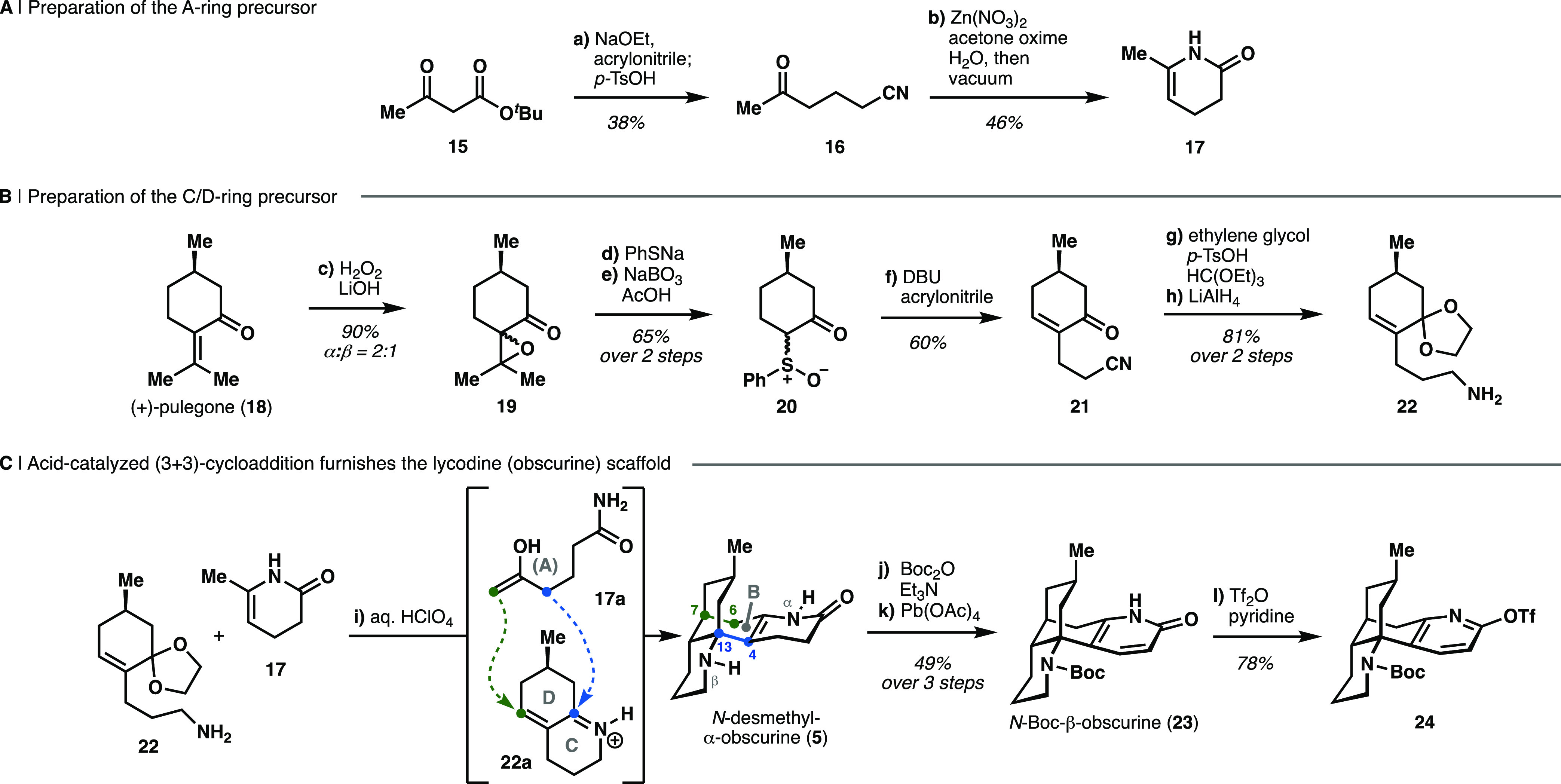
Synthesis of the Bicyclo[3.3.1]nonane Core in *N*-Desmethyl-α-obscurine through Formal (3 + 3)-Cycloaddition Reagents and conditions: (a) NaOEt, EtOH, 21 °C, then acrylonitrile, 0 to 21 °C, then TsOH, 145 °C (38%, > 13 g scale); (b) Zn(NO_3_)_2_·6H_2_O, acetone oxime, H_2_O, 90 °C, then vacuum, 120 °C (46%, > 2 g scale); (c) aq. H_2_O_2_, LiOH·H_2_O, MeOH, H_2_O, 21 °C (90%, 30 g scale); (d) PhSH, Na, THF, 21 °C, then **19**, 85 °C; (e) NaBO_3_·H_2_O, AcOH, 40 °C (65%, 2 steps, > 17 g scale); (f) DBU, ^*i*^PrOH, 0 °C, then acrylonitrile, 0 to 40 °C (60%, > 7 g scale); (g) ethylene glycol, *p*-TsOH, HC(OEt)_3_, 75 °C (97%); (h) LiAlH_4_, Et_2_O, 0 °C (84%, > 2 g scale); (i) aq. HClO_4_, 1,4-dioxane, 105 °C; (j) Boc_2_O, Et_3_N, THF, 60 °C (54%, 2 steps); (k) Pb(OAc)_4_, CHCl_3_, 21 °C (90%); (l) Tf_2_O, pyridine, CH_2_Cl_2_, −78 to 21 °C (78%).

The coupling partner that would lead to ring A, dihydropyridone **17**, was prepared from β-ketoester **15** through a Michael addition into acrylonitrile followed by decarboxylation to give nitrile **16**. Subsequent nitrile hydration and cyclization in vacuo delivered **17** in 18% overall yield (Scheme 1a).^29,31^

The C/D ring cycloaddition partner **22** was prepared from (+)-pulegone (**18**) in six steps and 28% overall yield (Scheme 1b).^32,33^ The sequence was initiated by Weitz–Scheffer-type epoxidation of the exocyclic olefin group of **18**, which provided a 1:2 mixture of epoxide isomers (**19**).^38,39^ Subsequent nucleophilic opening of the epoxide with sodium thiophenolate and concomitant retro-aldol reaction delivered the phenylthioether,^33^ which was selectively oxidized to sulfoxide **20** with sodium perborate.^34^ α-Alkylation of **20** with acrylonitrile, followed by thermal *syn*-elimination of phenylsulfenic acid gave enone **21**,^35,36^ which was protected as the ethylene glycol ketal and reduced with LiAlH_4_ to deliver primary amine **22**.^32^ The two building blocks (**17** and **22**) were ultimately coupled upon heating with perchloric acid (Scheme 1c). Under these conditions, oxygen-sensitive α,β-unsaturated iminium ion **22a** and the open-chain enolamide **17a** are presumably formed in situ and undergo the desired formal cycloaddition to furnish *N*-desmethyl-α-obscurine (**5**).^29,31,37^ Boc-protection of the piperidine nitrogen in **5** and dehydrogenation of the dihydropyridone ring using lead(IV) acetate provided *N*-Boc-β-obscurine (**23**) in 49% yield over three steps.

As an alternative to the oxidation of Boc-protected **5** using stoichiometric lead(IV) acetate, we investigated a photocatalytic dehydrogenation protocol.^40,41^ Our preliminary results demonstrated that *N*-Boc-**5** was readily oxidized to **23** (57% yield) in the presence of an iridium(III) photoredox catalyst (Ir[dF(CF_3_)ppy]_2_(dtbbpy)PF_6_) with potassium persulfate as the terminal oxidant upon irradiation with blue light (λ = 450 nm) under anoxic conditions. In the absence of light or the photoredox catalyst, only traces of product (6%) were formed in the best case, whereas under aerobic conditions complete decomposition of the substrate was noted (see Section S3.1 in the Supporting Information, SI). Despite attempts to optimize this reaction, we were unable to obtain yields comparable with those achieved with lead(IV) acetate (90%). Therefore, the latter conditions were employed for the preparation of large quantities of material. Finally, pyridone *O*-triflation of **23** delivered fully protected β-obscurine scaffold **24** in 78% yield.^29^

### Synthesis of (−)-Casuarinine H, (−)-8,15-Dihydrohuperzine A, and (+)-Lycoplatyrine B

Our envisioned route toward the lycodine alkaloids casuarinine H (**2**), 8,15-dihydrohuperzine A (**3**), and lycoplatyrine B (**4**) required the identification of suitable conditions to effect the bioinspired oxidative cleavage of the C9–N bond in protected tetracycle **24** or a related obscurine congener. To this end, we pursued several conditions for C–N cleavage and functionalization that included biocatalytic and transition metal-mediated approaches.

Biocatalytic methods were explored as a means to achieve a protecting group-free oxidation of the C9–N bond, reminiscent of the proposed biosynthetic tailoring process. Although the requisite biosynthetic enzymes have not been identified, we posited that other established biocatalysts capable of oxidizing C-heteroatom bonds could accept the bicyclo[3.3.1]nonane scaffold of **5** as a substrate while retaining site-selectivity. A screening set composed of 14 commercial and in-house heterologously expressed copper-^42,43^ and flavin-dependent oxidases,^44−46^ a pyrroloquinoline (PQQ) dependent dehydrogenase,^47^ a horseradish peroxidase (HRP),^48^ and a laccase/TEMPO redox mediator system^49^ was assembled. However, overview screenings under representative conditions did not identify any oxidation activity with unprotected substrate **5** (see SI Section S3.2 for details).

**Scheme 2:**
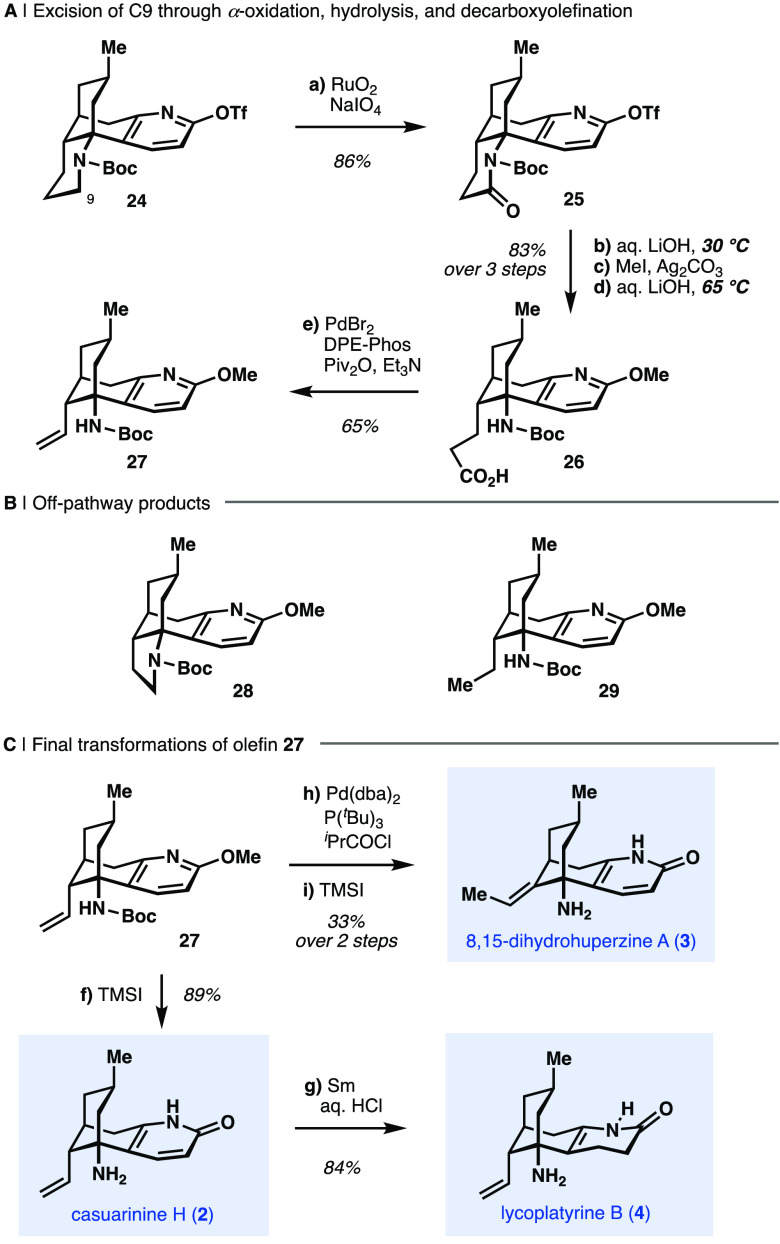
Synthesis of C-Ring Cleaved Lycodine Alkaloids from Protected β-Obscurine. Reagents and conditions: (a) RuO_2_·H_2_O, NaIO_4_, H_2_O, 21 °C, then **24**, ^*t*^BuOH, 60 °C (86%); (b) aq. 1 M LiOH, THF, 30 °C; (c) MeI, Ag_2_CO_3_, CHCl_3_, 75 °C (86%, 2 steps); (d) aq. 1 M LiOH, THF, 65 °C (97%); (e) PdBr_2_, DPE-Phos, Piv_2_O, Et_3_N, DMPU, 130 °C (65%); (f) TMSI, CHCl_3_, 65 °C (89%); (g) Sm, aq. 3 M HCl, 0 to 21 °C (84%); (h) Pd(dba)_2_, P(^*t*^Bu)_3_, ^*i*^PrCOCl, toluene, 90 °C (81%); (i) TMSI, CHCl_3_, 65 °C (41%).

We therefore sought to examine other established chemical conditions for the oxidation of carbamate-protected saturated nitrogen heterocycles. While methods employing iron^50^ and copper^51^ redox mediators in combination with peroxides failed to generate the anticipated enamine or enamide products, we observed that substoichiometric quantities of RuO_2_ with sodium periodate as stoichiometric oxidant in a mixture of ^*t*^BuOH and water resulted in piperidine oxidation to yield **25** (Scheme 2a).^52,53^ Although oxidation under these conditions by the presumed in situ generated RuO_4_ catalyst was expected to give the corresponding amino acid (i.e., following hydrolysis of an intermediate C-ring iminium ion and oxidation of the resulting aldehyde), cyclic imide **25** was obtained in 86% yield. Additional experiments demonstrated that the electronically deactivating triflyl group on the pyridone oxygen was critical to the success of the piperidine oxidation–oxidation of derivatives of **25** bearing methyl, benzyl, or SEM groups instead of the triflyl moiety proved unsuccessful under identical conditions.

We envisioned that hydrolysis of imide **25** followed by decarboxyolefination of the resulting carboxylic acid could offer an attractive strategy to excise C9 and install the required unsaturation at C10–C11. Treatment of **25** with aqueous LiOH at the elevated temperatures required for imide hydrolysis resulted in undesired concomitant triflate cleavage. Therefore, a methyl ether was introduced in place of the triflate prior to imide hydrolysis to yield carboxylic acid **26**. Unfortunately, subjecting **26** to classic Kochi oxidative decarboxylation conditions^54^ failed to deliver alkene **27**. Additionally, an attempted Hunsdiecker-type decarboxyhalogenation^55^ resulted in the C-ring contracted pyrrolidine **28** (Scheme 2b), presumably the result of an S_N_2 displacement of the intermediate alkyl halide. While more recently developed decarboxyolefination conditions using metallo-organo-^56^ or organo-photocatalysts^57^ in combination with cobalt-based dehydrogenation catalysts furnished olefin **27** in 50% yield, a competing protodemetalation pathway leading to ethyl derivative **29** hindered further optimization of this process. Alternatively, desired terminal olefin **27** was obtained in higher yield (65%) through a Pd(0)-catalyzed decarbonylative elimination of an in situ-generated mixed anhydride of **26**.^58^ Deprotection of **27** using TMSI^12^ completed the first total synthesis of the neuroprotective compound (−)-casuarinine H (**2**, Scheme 2c).^25^ Semireduction of the pyridone moiety in **2** with samarium metal in aqueous HCl^59^ cleanly yielded (+)-lycoplatyrine B (**4**)^26^ in 84% yield, also constituting the first total synthesis of this *Lycopodium* alkaloid. Furthermore, treatment of terminal olefin **27** with an in situ-generated palladium hydride catalyst effected isomerization to the internal (*E*)-alkene in 81% yield.^60^ A subsequent TMSI-mediated deprotection delivered (−)-8,15-dihydrohuperzine A (**3**).^24,61^

The spectroscopic data for synthetic (−)-casuarinine H (**2**), (+)-lycoplatyrine B (**4**), and (−)-8,15-dihydrohuperzine A (**3**) were in full agreement with those reported upon isolation of these natural products from the producing organisms.^24−26^ Taking advantage of this late-stage diversification approach, the target alkaloids **2**–**4** were prepared in 16 to 17 steps (longest linear sequence, LLS) and 1.7–4.5% overall yield from (+)-pulegone.

### Synthesis of Lycoplatyrine A and Lycopladine F

**Scheme 3:**
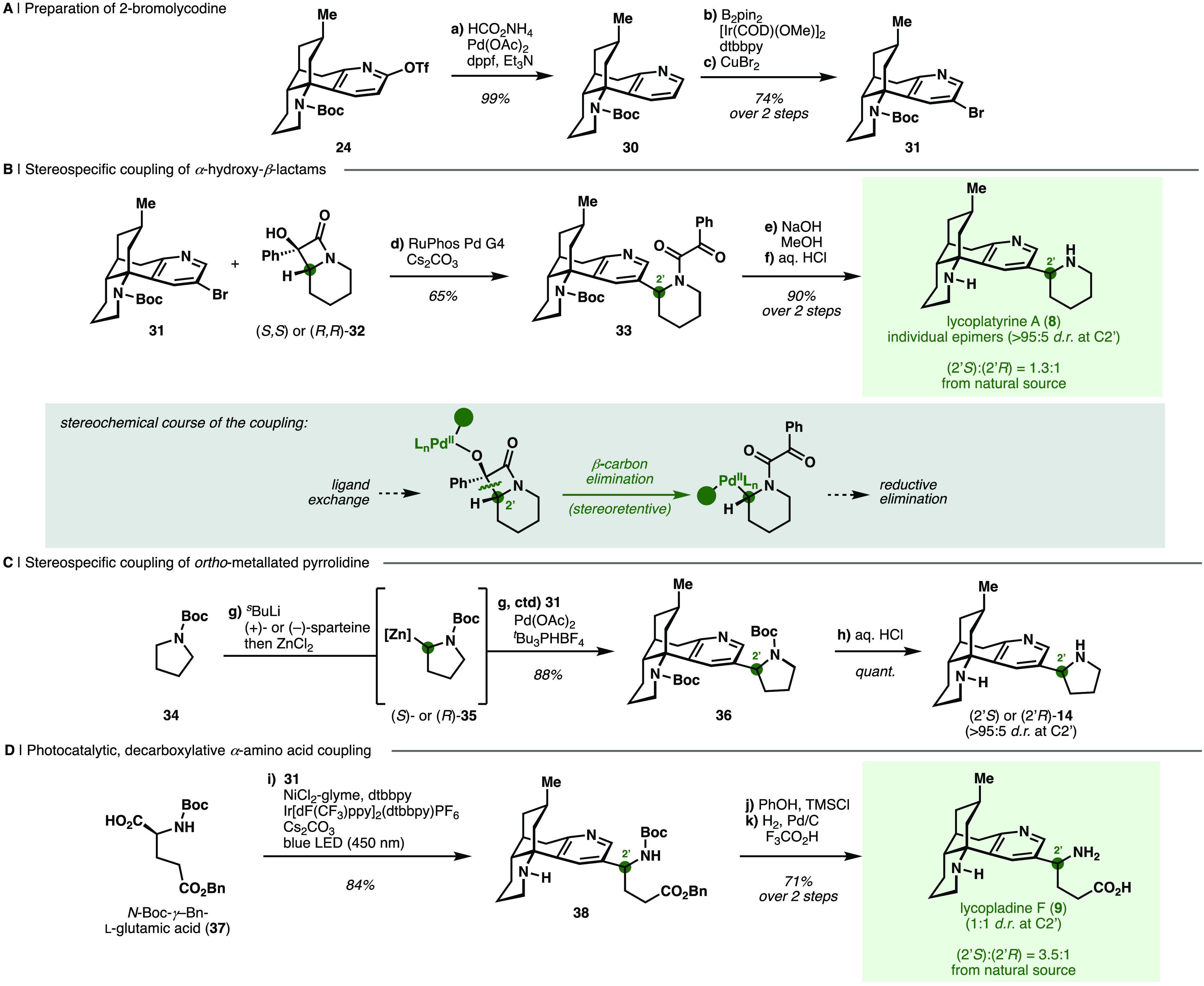
Couplings of a Site-Selectively Functionalized Lycodine Congener in the Syntheses of C2-Substituted Alkaloids Reagents and Conditions: (a) HCO_2_NH_4_, Pd(OAc)_2_, dppf, Et_3_N, DMF, 60 °C (99%); (b) B_2_ pin_2_, [Ir(COD)(OMe)]_2_, dtbbpy, THF, 80 °C; (c) CuBr_2_, MeOH, H_2_O, 80 °C (74%, 2 steps); (d) RuPhos Pd G4, Cs_2_CO_3_, toluene, 70 °C [(2′*S*)-**33**: 65%, (2′*R*)-**33**: 65%, **33** as epimeric mixture at C2′ with *rac*-**32**: 72%]; (e) NaOH, MeOH, 1,4-dioxane, 70 °C (f) aq. 6 M HCl, 70 °C [(2′*S*)-**8**: 90%, (2′*R*)-**8**: 68%; 2 steps]; (g) ^*s*^BuLi, (+)- or (−)-sparteine, MTBE, −78 °C, then ZnCl_2_, THF, −78 to 21 °C, then **31**, Pd(OAc)_2_, ^*t*^Bu_3_PHBF_4_, MTBE, 60 °C [(2′*S*)-**36**: 55%, (2′*R*)-**36**: 88%]; (h) aq. 6 M HCl, 21 °C [(2′*S*)-**14**: quantitative, (2′*R*)-**14**: 70%]; (i) **31**, NiCl_2_-glyme, dtbbpy, Ir[dF(CF_3_)ppy]_2_(dtbbpy)PF_6_, Cs_2_CO_3_, DMF, 450 nm LED, 21 °C (84%); (j) PhOH, TMSCl, CH_2_Cl_2_, 21 °C; (k) 500 psi H_2_, Pd/C, CF_3_CO_2_H, MeOH, 21 °C (71%, 2 steps).

For the synthesis of *Lycopodium* alkaloids bearing substituents at C2 (i.e., **8**–**9**), we envisioned a cross-coupling approach in which the key β-obscurine intermediate **24** would be elaborated to a selectively C2-functionalized lycodine derivative to serve as a common coupling partner. Accordingly, protected β-obscurine **24** was deoxygenated in the presence of a palladium catalyst and ammonium formate as reductant to deliver *N*-Boc lycodine (**30**). Subsequent iridium-catalyzed *meta*-selective C–H borylation^29,62^ of the pyridine A-ring and bromodeborylation^63^ furnished 2-bromolycodine (**31**) (Scheme 3a).

Lycoplatyrine A (**8**) features a C2 piperidine substituent as an epimeric mixture of undetermined absolute configuration,^26^ which we anticipated could be installed through the coupling of **31** with an α-functionalized piperidine derivative (Scheme 3b). We specifically envisioned the application of a method recently disclosed by our laboratory in which α-hydroxy-β-lactams such as **32** serve as surrogates for α-metalated *N*-heterocycles in a palladium-catalyzed coupling with aryl halides.^20^ This method was particularly attractive due to the mild and stereospecific nature of the cross-coupling, although the use of pyridyl bromides had not been previously demonstrated. As proposed, the coupling of **31** with racemic lactam **32**, prepared from the corresponding piperidine-derived 2-oxophenylacetamide through a Norrish-Yang reaction,^20^ delivered **33** as a mixture of epimers at C2′. Cleavage of the 2-oxophenylacetamide and Boc-protecting groups under sequential basic and acidic conditions yielded lycoplatyrine A (**8**) as a 1:1.5 mixture of the anticipated C2′ epimers.

According to the previously proposed mechanism for this coupling, the hydroxy group of the lactam coordinates to the palladium center before irreversible C–C bond cleavage (β-carbon elimination) driven by the release of ring strain in **32** delivers a C2′-palladated species in a stereoretentive manner (Scheme 3b, gray box).^20,64^ We therefore anticipated that the use of enantiomerically pure lactams (2′*S*)- and (2′*R*)-**32** would enable the stereospecific piperidinylation of the lycodine scaffold at C2, and thus allow the assignment of absolute configurations at C2′ in naturally occurring alkaloid **8**.

To obtain α-hydroxy-β-lactam **32** in enantioenriched form, we first investigated enzymatic resolution methods. Despite extensive reaction engineering, selectivity for an enzymatic hydrolytic kinetic resolution^65,66^ of acetylated tertiary alcohol **32** with pig liver esterase (PLE) and lipase A from *C. antarctica* (CalA) was poor and therefore not synthetically useful (*E* ≤ 7) (see SI Section S3.5). Alternatively, enantiomerically resolved lactams (2′*S*)- and (2′*R*)-**32** were obtained from preparative chiral supercritical fluid chromatography (SFC).^20^ Coupling of lactams (2′*S*)- and (2′*R*)-**32** with lycodine bromide **31** gave single epimers of **33** in 65% yield, which were deprotected to provide single epimers of lycoplatyrine A (**8**) in 4.7% overall yield over 16 steps from (+)-pulegone (LLS). Comparison of the spectral data of single epimers of synthetic **8** with data for naturally derived **8** revealed a slight excess of the (2′*S*)-**8** epimer in material isolated from natural sources (*d.r*. 1.3:1).^26^ The cross-coupling product obtained using racemic **32** was also enriched in the same epimer (*d.r*. 1.5:1, vide supra), suggesting that the chiral lycodine scaffold exerts a low level of enantiodiscrimination and enantiotopic face discrimination in both the synthetic and natural coupling processes.

Indeed, our success in preparing single epimers of lycoplatyrine A (**8**) rested on the stereospecific coupling of α-hydroxy-β-lactams as surrogates for α-metalated piperidines, which otherwise typically suffer from low yields and poor stereoselectivities in the metalation step.^67,68^ Although an analogous β-lactam-based cross-coupling for five-membered nitrogen heterocycles is precluded due to the inaccessibility of the five-membered analogues of **32** with established photochemical methods,^69^ α-metalated pyrrolidines are excellent stereoselective coupling partners. These reagents set the stage for the preparation of the pyrrolidine analog of lycoplatyrine A (“pyrrolo-lycoplatyrine A”, **14**), which is hypothesized to be an intermediate in the biosynthesis of other lycodine-derived congeners including lycopladine F (**9**).^27^ For the synthesis of *N*-Boc pyrrolo-lycoplatyrine A (**36**), we turned to a method by Campos and co-workers^70^ for the stereoselective α-arylation of *N*-Boc-pyrrolidine (**34**) (Scheme 3c). Enantioselective *ortho*-lithiation of **34** in the presence of either (+)- or (−)-sparteine,^71^ transmetalation to form the corresponding organozinc species (**35**), and subsequent palladium-catalyzed coupling to lycodine bromide (**31**) delivered single C2′-epimers of the desired product (**36**) in high yield (88%). Subsequent deprotection provided each of the two C2′-epimers of pyrrolo-lycoplatyrine A (**14**) in 15 steps from (+)-pulegone (7% overall, LLS).

We sought to similarly access lycopladine F (**9**) via a direct coupling approach where the necessary amino acid moiety is appended at C2 of lycodine bromide (**31**, Scheme 3d). To this end, iridium-catalyzed photoredox conditions effected activation of bis-protected glutamic acid **37** through single-electron oxidation of the cesium carboxylate, followed by decarboxylative C–C bond scission and nickel-catalyzed C(*sp*^3^)–C(*sp*^2^) coupling with aryl bromide **31** to deliver protected lycopladine F (**38**) in 84% yield.^72^ A low nickel loading (1 mol %) was necessary to attenuate consumption of bromide **31** in a nonproductive protodehalogenation pathway and achieve good yields of **38**. Removal of both Boc protecting groups followed by hydrogenolytic cleavage of the benzyl ester in the presence of trifluoroacetic acid yielded lycopladine F (**9**) in 71% yield as a 1:1 mixture of epimers (4.8% yield over 16 steps LLS). The analytical data obtained for the synthetic material matched those reported for the natural material, which was isolated from *Lycopodium complanatum* as a 3.5:1 mixture of (2′*S*):(2′*R*)-epimers.^27^ We expect access to pyrrolo-lycoplatyrine A (**14**) and lycopladine F (**9**) to set the stage for studies into the biosynthesis of the latter natural product.^27^

## Conclusions

In summary, we have developed the first total syntheses of lycodine alkaloids casuarinine H (**2**), lycoplatyrine B (**4**), lycoplatyrine A (**8**), and lycopladine F (**9**) and a total synthesis of 8,15-dihydrohuperzine A (**3**) employing the readily accessible tetracycle *N*-desmethyl-β-obscurine (**6**) as a common intermediate. A series of bioinspired modifications of the piperidine C-ring in **6**, including oxidative ring cleavage, C–C bond scission with carbon atom excision, and olefin isomerization delivered tricyclic congeners **2**–**4**. Conversion of the pyridone A-ring in **6** to the corresponding pyridine (**7**) and site-selective C–H functionalization to ultimately afford bromopyridine **31** enabled direct cross-couplings with saturated azacycles or an amino acid to complete the syntheses of C2-derivatized lycodine alkaloids lycoplatyrine A (**8**) and lycopladine F (**9**). The general late-stage peripheral derivatization and C–C functionalization strategies outlined herein may provide a basis for synthetic access to an even wider range of *Lycopodium* alkaloids. Our synthetic studies toward these compounds should also set the stage for a broader, more systematic assessment of their biosynthesis and bioactivity.^25,26,61^ Biological activities exerted by these natural products may include a range of neuroprotective effects such as those observed for huperzine A,^4,5^ for example the attenuation of both glutamate-induced neurotoxicity and free radical-mediated oxidative stress.

## Supporting Information Available

Experimental procedures, additional experimental results, and compound characterization (PDF)
