# Supplementary material for: Bioinspired Diversification Approach Toward the Total Synthesis of Lycodine-Type Alkaloids
Source: J Am Chem Soc. 2021 Mar 17;143(12):4732–40. doi: 10.1021/jacs.1c00457 (PMC8017526; doi:10.1021/jacs.1c00457)
Supplement: Supplementary file 1 — ja1c00457_si_001.pdf [file ja1c00457_si_001.pdf]

Supporting Information:

A Bioinspired Diversification Approach Toward the Total Synthesis of Lycodine-Type Alkaloids

Hannah M. S. Haley, Stefan E. Payer, Sven M. Papidocha, Simon Clemens, Jonathan Nyenhuis and Richmond Sarpong*

Department of Chemistry, University of California, Berkeley, CA 94720, United States

Corresponding Author

*rsarpong@berkeley.edu (R.S.)

Table of Contents

1. General Information 4

2. Preparative Procedures 5

2.1. Synthesis of 6-methyl-3,4-dihydropyridin-2(1*H*)-one (**17**) 5

2.2. Synthesis of (+)-pulegone epoxide (**19**) 5

2.3. Synthesis of (5*R*)-5-methyl-2-(phenylthio)cyclohexan-1-one (**S1**) 6

2.4. Synthesis of (5*R*)-5-methyl-2-(phenylsulfinyl)cyclohexan-1-one (**20**) 7

2.5. Synthesis of (*R*)-3-(4-methyl-6-oxocyclohex-1-en-1-yl)propanenitrile (**21**) 8

2.6. Synthesis of (*R*)-3-(9-methyl-1,4-dioxaspiro[4.5]dec-6-en-6-yl)propanenitrile (**S2**) 9

2.7. Synthesis of (*R*)-3-(9-methyl-1,4-dioxaspiro[4.5]dec-6-en-6-yl)propan-1-amine (**22**) 10

2.8. Synthesis of *N*-desmethyl-*α*-obscurine (**5**) 11

2.9. Synthesis of *N*-Boc-*α*-obscurine (**S3**) 11

2.10. Synthesis of *N*-Boc-*β*-obscurine (**23**) 11

2.11. Synthesis of 1-triflyl-*N*-Boc-lycodine (**24**) 11

2.12. Synthesis of 9-oxo-1-triflyl-*N*-Boc-lycodine (**25**) 12

2.13. Synthesis of 9-oxo-*N*-Boc-*β*-obscurine (**S4**) 13

2.14. Synthesis of 1-methoxy-9-oxo-*N*-Boc-lycodine (**S5**) 14

2.15. Synthesis of aminoacid **26** 15

2.16. Synthesis of 1'-methyl-*N*-Boc-casuarinine H (**27**) 16

2.17. Synthesis of (–)-casuarinine H (**2**) 17

2.18. Synthesis of (+)-lycoplatyrine B (**4**) 18

2.19. Synthesis of 1'-methyl-*N*-Boc-8,15-dihydrohuperzine A (**S6**) 19

2.20. Synthesis of (–)-8,15-dihydrohuperzine A (**3**) 20

2.21. Synthesis of *N*-Boc-lycodine (**30**) 21

2.22. Synthesis of *N*-Boc lycodine boronic ester (**S7**) 21

2.23. Synthesis of *N*-Boc-2-bromolycodine (**31**) 21

2.24. Synthesis of protected piperidine adduct **33** 22

2.25. Synthesis of lycoplatyrine A (**8**) 24

2.26. Synthesis of *N*-Boc pyrrolidine adduct **36** 26

2.27. Synthesis of deprotected pyrrolidine adduct **14** 28

2.28. Synthesis of protected amino ester adduct **38** 29

2.29. Synthesis of lycopladine F (**9**) 30

3. Supplementary Results 32

3.1. Photocatalytic dehydrogenation of *N*-Boc-α-obscurine (**S3**) 32

3.2. Biocatalytic oxidation methods 33

3.3. Synthesis of *trans*-8-oxo-7-phenyl-1-azabicyclo[4.2.0]octan-7-yl acetate (**S13**) 34

3.4. Synthesis of *trans*-8-oxo-7-phenyl-1-azabicyclo[4.2.0]octan-7-yl butyrate (**S14**) 35

3.5. Enzymatic kinetic resolution of the β-lactam coupling partner 36

4. Comparison of NMR spectroscopic data for synthetic and isolated alkaloids 39

5. References 46

6. NMR Spectra 48

1. General Information

Reactions were performed under inert nitrogen atmosphere in oven-dried glassware unless otherwise noted. Room temperature (r.t.) refers to a standard 21 °C. All reactions were stirred with Teflon®-coated magnetic stir bars and were monitored by thin layer chromatography (TLC) performed on Macherey-Nagel TLC Silica gel 60 F254 precoated glass plates (0.25 mm thickness). Substances were visualized under UV light (λ = 254 nm) and/or staining with basic $KMnO_4$ or *p*-anisaldehyde solutions. Chromatographic purification was performed on silica gel (SiliCycle, 40–63 µm mesh).

All chemicals were purchased from commercial suppliers and were used without further purification unless otherwise stated. THF, toluene, Et_2O and Et_3N were dried by passage over a column of activated alumina (JC Meyer Phoenix SDS Solvent System). CH_2Cl_2 and diisopropylamine (DIPA) were distilled over anhydrous CaH_2. Anhydrous EtOH, $CHCl_3$ and pyridine were obtained in sealed bottles from commercial suppliers.

^1H and ^{13}C-NMR spectra were recorded on Bruker spectrometers operating at 300, 400, 500, 600 MHz for ^1H and 125, 151 MHz for ^{13}C experiments. Chemical shifts are reported relative to TMS as parts per million (ppm) and are referenced to the solvent signals [$\delta(^1H)$ = 7.26 ppm and $\delta(^{13}C)$ = 77.16 ppm for $CDCl_3$; $\delta(^1H)$ = 3.31 ppm and $\delta(^{13}C)$ = 49.00 ppm for methanol-d4; $\delta(^1H)$ = 7.16 ppm and $\delta(^{13}C)$ = 128.06 ppm for C_6D_6]. Multiplets are reported as follows: s (singlet), d (doublet), t (triplet), q (quartet), dd (doublet of doublet), dt (doublet of triplet), m (multiplet), br s (broad singlet).

Infrared spectra (IR) were recorded on a Bruker ALPHA FT-IR spectrometer as thin films and are reported in [cm^{-1}]. Specific optical rotations were measured on a Perkin-Elmer 241 polarimeter in a cuvette of 10 cm optical pathlength. Concentrations (c) are given in grams of solute per 100 ml of solution.

LC-MS measurements were performed on a Shimadzu 2020 LC-MS system equipped with a Hypersil Gold column (50 mm × 4.6 mm, 3 µm particle size) using the following elution method: solvent A: H_2O (+ 0.1% v/v HCO_2H), B: MeCN (+ 0.1% v/v HCO_2H); 30% to 95% B in 6 min, 1 mL min^{-1}. The quadrupole MS was operated in ESI (+) mode (–3.5 kV cone voltage).

GC-MS with volatile compounds was performed on a Shimadzu QP2010 SE system equipped with a SHRXI-5MS capillary column (30 m × 0.25 mm × 0.25 µm film) operated in positive ESI mode (–1.5 kV cone voltage) and with He as carrier gas. The default method consisted of a linear temperature gradient (10 °C min^{-1}) between 80 and 250 °C (250 °C injector temp.)

HR-MS spectra were recorded at the QB3/Chemistry Mass Spectrometry Facility at the University of California, Berkeley, on a Finnigan/Thermo LTQ-FT instrument (ESI), and the data acquired was processed using the XcaliburTM software.

2. Preparative Procedures

2.1. Synthesis of 6-methyl-3,4-dihydropyridin-2(1*H*)-one (17)

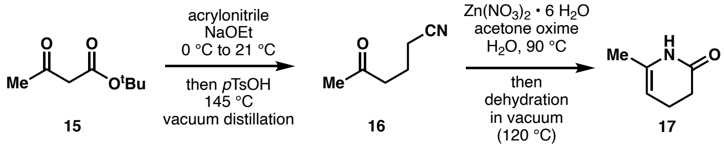

The title compound was prepared according to a literature procedure in 18% overall yield (lit. 19%).[1]

2.2. Synthesis of (+)-pulegone epoxide (19)

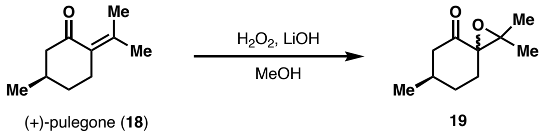

Adapted from literature.[2] A solution of lithium hydroxide monohydrate (1.07 g, 25.6 mmol, 0.13 equiv) in water (10 mL) was added dropwise to a mixture of (+)-pulegone (30.0 g, 197 mmol, 1.0 equiv) and hydrogen peroxide (35% aq. solution, 21.6 mL, 296 mmol, 1.5 equiv) in methanol (150 mL) at 21 °C over the course of 5 min. The mixture was stirred at 21 °C for 6 h, after which TLC (hexanes/EtOAc = 2:1) showed full conversion of the starting material. Brine (100 mL) was added, followed by extraction with EtOAc (3 × 150 mL). The combined organic extracts were washed with brine (3 × 150 mL), dried over $MgSO_4$ and the solvent was removed under vacuum (40 °C) to give epoxide **19** as colorless oil, which slowly crystallizes at room temperature (30.0 g, 90%, 2:1 mixture of diastereomers).

^{1}H-NMR ($CDCl_3$, 500 MHz) δ_H [ppm] 2.60 (d, *J* = 13.5 Hz, 0.3H, β-isomer), 2.41 (s, 2H), 2.19 (td, *J* = 13.1, 4.2 Hz, 0.7H, α-isomer), 2.06–1.70 (m, 4H), 1.42 (s, 3H), 1.22 (s, 2H, α-isomer), 1.20 (s, 1H, β-isomer), 1.12–0.99 (m, 3H).

LC-MS (ESI^+) *m/z* = 169 ($M+H^+$), 210 ($M+MeCN+H^+$).

Spectral data in agreement with literature.[3]

2.3. Synthesis of (5*R*)-5-methyl-2-(phenylthio)cyclohexan-1-one (S1)

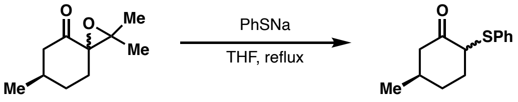

19 **S1**

Adapted from literature.[4] Thiophenol (23.8 mL, 25.5 g, 232 mmol, 2.05 equiv) was dissolved in anhydrous THF (500 mL) in a 1.0 L round-bottom flask with stir bar. Pieces of flattened sodium metal (5.17 g, 226 mmol, 2.0 equiv) are added and the developing colorless suspension was stirred at room temperature overnight. Pulegone epoxide **19** (2:1 mixture of α- and β-isomers, 19.0 g, 113 mmol, 1.0 equiv) was dissolved in anhydrous THF (25 mL) in a 100 mL round bottom flask and was added dropwise via cannula to the PhSNa suspension over the course of 30 min. The mixture was heated to 85 °C and kept at reflux for 7 h after which TLC (hex/EtOAc 2:1, UV/*p*-anisaldehyde, $R_{f,prod}$ = 0.71, $R_{f,sm}$ = 0.46; diluted sample in a few µL MeOH) confirmed full conversion of the epoxide. The reaction was cooled to room temperature and water (200 mL) was added, followed by extraction with ethyl acetate (3 × 150 mL). The combined organic extracts were washed with sat. aq. K_2CO_3 (3 × 150 mL) and brine (200 mL), dried over $MgSO_4$ and the solvent was evaporated under reduced pressure (40 °C) to give 25.8 g of **S1** as a yellow-brownish oil, which slowly turns into to a waxy solid upon storage at 4 °C (104 % crude yield). The mixture of diastereomers was used in the next step without further purification.

^{1}H-NMR (400 MHz, $CDCl_3$) δ_H [ppm] 7.53–7.47 (m, 0.6H), 7.42–7.34 (m, 2H), 7.34–7.20 (m, ~3.5H, superimposed on solvent), 3.87 (ddd, *J* = 11.3, 5.8, 1.2 Hz, 0.4H), 3.73 (ddd, *J* = 4.6, 2.9, 1.5 Hz, 0.5H), 2.79 (dd, *J* = 13.7, 12.2 Hz, 0.5H), 2.68 (ddd, *J* = 12.9, 3.8, 2.1 Hz, 0.6H), 2.38–1.59 (m, 8H), 1.45–1.32 (m, 1.2H), 1.24–1.13 (m, 0.8H), 1.10–0.96 (m, 3.9H).

^{13}C-NMR (100 MHz, $CDCl_3$) δ_C [ppm] 132.5, 131.6, 129.2, 129.1, 127.6, 127.4, 57.5, 54.5, 49.2, 45.5, 34.8, 33.4, 33.0, 31.7, 29.4, 22.2, 21.7 (C=O signal was not observed in spectrum)

LC-MS (ESI^+) *m/z* = 340 (100), 262 (66, M+ MeCN+H^+), 381 (65), 194 (53), 142 (49), 295 (45), 358 (38), 408 (31), 278 (21), 221 (11, M+H^+).

IR ν [cm^{-1}] 2953, 2926, 1710 (C=O), 1438.

HR-MS (ESI^+) calculated mass for $C_{13}H_{17}OS$ [(M+H^+)]: *m/z* = 221.0995; found: 221.0998.

2.4. Synthesis of (5*R*)-5-methyl-2-(phenylsulfinyl)cyclohexan-1-one (20)

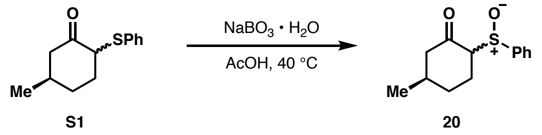

Crude thioether **S1** (25.0 g) was dissolved in acetic acid (glacial, 225 mL, 9 mL/g crude) in a 500 mL round bottom flask and heated to 40 °C. Sodium perborate monohydrate (12.4 g, 125 mmol, 1.1 equiv) was ground to a fine powder with a mortar before adding it to the dissolved thioether in ca. 2 g portions over the course of 5 min. The suspension was stirred at 40 °C for 45 min, after which the mixture clarified and TLC (hexanes/EtOAc 2:1, UV/p-anisaldehyde, $R_{f,prod}$ = 0.25, brown) confirmed full conversion of the starting material. The reaction was cooled to 0 °C in an ice bath, and NH_4OH (14.8 M, 265 mL) was added slowly (!) over 1.5 h with a dropping funnel whilst stirring to quench the acetic acid. The resulting colorless suspension was diluted with water (100 mL) and the pH adjusted to 7–8 by dropwise addition of aqueous ammonia solution (14.8 M). The aqueous mixture was extracted with CH_2Cl_2 (3 × 150 mL), the combined organic extracts washed with brine (150 mL), and dried over $MgSO_4$. Evaporation of the solvent under reduced pressure (20 °C) gave 26.5 g of a thick, brownish oil (99% crude over 2 steps), which was purified by column chromatography using a gradient of hexanes/EtOAc 2:1–1:1–1:2. The title compound **20** was obtained as colorless oil, which solidifies upon standing at room temperature (17.3 g, 65% over two steps, mixture of diastereomers).

^{1}H-NMR (400 MHz, $CDCl_3$) δ_H [ppm] 7.68–7.61 (m, 1H), 7.58–7.54 (m, 1H), 7.50–7.46 (m, 3H), 3.66 (dd, *J* = 10.9, 5.9 Hz, 0.17H), 3.37–3.30 (m, 0.82H), 2.62–2.47 (m, 1.4 H), 2.38–2.20 (m, 0.7H), 2.15–1.90 (m, 3.5H), 1.81 (q, *J* = 6.9 Hz, 1H), 1.40–1.29 (m, 0.7 H), 1.06 (d, *J* = 6.6 Hz, 1.2H), 1.01–0.96 (m, 1.8 H).

^{13}C-NMR (100 MHz, $CDCl_3$) δ_C [ppm] 131.7, 131.5, 131.0, 129.4, 129.3, 129.2, 129.0, 126.0, 124.8, 124.7, 124.6, 75.3, 73.4, 73.1, 71.7, 51.4, 50.5, 50.4, 50.1, 34.1, 33.6, 33.57, 32.3, 32.2, 29.7, 29.6, 27.6, 25.1, 24.0, 22.9, 21.9, 21.8, 21.6, 21.3.

LC-MS (ESI^+) *m/z* = 278 (100, M+MeCN+H^+), 300 (52), 495 (29), 473 (27), 237 (25, M+H^+), 279 (23), 341 (13), 301 (12).

IR ν [cm^{-1}]3058, 2953, 1710 (C=O), 1442, 1145–1022 (sulfoxide), 747, 689.

HR-MS (ESI^+) calculated mass for $C_{13}H_{17}O_2S$ [(M+H^+)]: *m/z* = 237.0944; found: 237.0950.

2.5. Synthesis of (*R*)-3-(4-methyl-6-oxocyclohex-1-en-1-yl)propanenitrile (21)

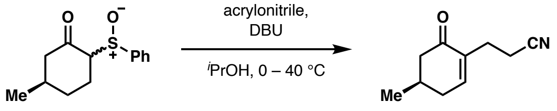

20 **21**

Sulfoxide **20** (18.4 g, 77.9 mmol, 1.0 equiv) was dissolved in in isopropanol (430 mL) and cooled to 0 °C. 1,8-Diazabicyclo[5.4.0]undec-7-ene (DBU, 23.2 mL, 156 mmol, 2.0 equiv) was added dropwise over 10 minutes. The yellowish solution was stirred for 15 min at 0 °C before acrylonitrile (6.67 mL, 101 mmol, 1.3 equiv) was added with a syringe pump (0.25 mL min^{-1}) at 0 °C. The reaction was allowed to warm to room temperature and stirred for 2 h before heating to 40 °C for another 2 h, upon which TLC (*n*-hexane/EtOAc 2:1, UV/*p*-anisaldehyde, $R_{f,prod}$ = 0.33, purple to green to yellow) confirmed full consumption of starting material. Volatile components were removed under vacuum (40 °C) and the black-brown oily residue was taken up in brine (200 mL). The aq. phase was extracted with EtOAc (3 × 200 mL) and the combined extracts were washed with 1 M aq. HCl (2 × 150 mL) and brine (150 mL). After drying over $MgSO_4$, the solvent was removed under reduced pressure (40 °C) and the brown-red oil was purified by silica gel chromatography (*n*-hexane/EtOAc 2:1) to afford nitrile **21** (7.68 g, 60%) as yellow oil. A yellow impurity is frequently observed to coelute with the product and can be removed by passing the oily product through a plug of neutral Al_2O_3 (*n*-hexane/EtOAc 2:1).

^{1}H-NMR (400 MHz, $CDCl_3$) δ_H [ppm] 6.90 (dd, *J* = 5.6, 2.7 Hz, 1 H), 2.62 – 2.36 (m, 6 H), 2.32 – 2.00 (m, 3 H), 1.06 (d, *J* = 6.3 Hz, 3 H).

^{13}C-NMR (100 MHz, $CDCl_3$) δ_C [ppm] 199.2, 148.1, 135.7, 119.4, 46.4, 34.4, 30.6, 26.5, 21.2, 17.0.

GC-MS (ESI^+) *m/z* = 163 (30, M^+), 148 (52, [M–"CH_3"]$^+$), 121 (53), 53 (58), 81 (100).

Spectral data is in agreement with literature.[5]

2.6. Synthesis of (*R*)-3-(9-methyl-1,4-dioxaspiro[4.5]dec-6-en-6-yl)propanenitrile (S2)

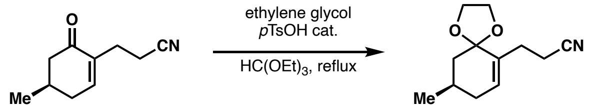

21 **S2**

Adapted from literature[6]: *Only a modest conversion of 21% (GC-MS, ^{1}H-NMR) was observed under the described conditions and an increase of ethylene glycol equivalents at lower temperature was necessary.*

To nitrile **21** (7.68 g, 47.0 mmol, 1.0 equiv) was added ethylene glycol (anhydrous, 34.1 mL, 612 mmol, 13.0 equiv), triethyl orthoformate (anhydrous, 47.0 mL, 282 mmol, 6.0 equiv), and *p*-toluene sulfonic acid monohydrate (44.5 mg, 0.23 mmol, 0.5 mol%) and the reaction mixture was heated to 75 °C. After stirring for 2 hours, TLC (*n*-hexane/EtOAc 2:1, *p*-anisaldehyde) confirmed full conversion of the starting material to a product with slightly higher R_f, which was invisible under UV-light. The reaction was cooled to approx. 40 °C and anhydrous K_2CO_3 (ca. 2 g) was added. The yellow mixture was concentrated under reduced pressure (40–45 °C) and the oily residue was purified by silica gel chromatography (*n*-hexane/EtOAc 2:1) to afford ketal **S2** (9.4 g, 97%) as pale-yellow oil.

^{1}H-NMR (400 MHz, $CDCl_3$) δ_H [ppm] 5.84 (d, *J* = 4.4 Hz, 1H), 4.07–3.91 (m, 4H), 2.54–2.48 (m, 2H), 2.36 (t, *J* = 7.4 Hz, 2H), 2.15 (dt, *J* = 17.7, 4.9 Hz, 1H), 1.95–1.82 (m, 2H), 1.71–1.63 (m, 1H), 1.31 (t, *J* = 13.0 Hz, 1H), 0.96 (d, *J* = 6.5 Hz, 3H).

^{13}C-NMR (100 MHz, $CDCl_3$) δ_C [ppm] 134.4, 132.0, 120.1, 108.3, 65.4, 64.3, 41.8, 34.0, 27.6, 26.3, 21.5, 17.8.

GC-MS (ESI^+) *m/z* = 207 (0.5, M^+), 165 (47, $[M–CH_2CN–H]^+$), 125 (100).

HR-MS (ESI^+) calculated mass for $C_{12}H_{18}NO_2$ [$(M+H^+)$]: *m/z* = 208.1332; found: 208.1359.

The number of protons in literature[6] does not match the title compound and the here acquired NMR data.

2.7. Synthesis of (*R*)-3-(9-methyl-1,4-dioxaspiro[4.5]dec-6-en-6-yl)propan-1-amine (22)

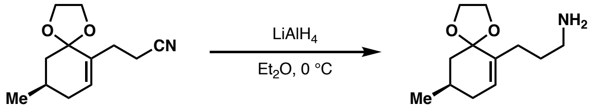

S2 22

Adapted from literature.[1] Ketal **S2** (2.85 g, 13.8 mmol, 1.0 equiv) was dissolved in anhydrous Et_2O (57.0 mL) and cooled to 0 °C. $LiAlH_4$ (2.11 g, 55.6 mmol, 4.04 equiv) was added portion-wise over 12 minutes and the grey suspension was stirred for 4 h at 0 °C. Upon full consumption of starting material (TLC) the reaction was worked up according to the Fieser & Fieser protocol: The reaction mixture was diluted with Et_2O (57 mL) and water (2.15 mL) was added dropwise (violent gas evolution) at 0 °C. 15% aq. NaOH solution (2.15 mL) was added and the mixture was stirred for 20 minutes at 0 °C. Water (6.30 mL) was added and the mixture was stirred for 20 minutes at room temperature. $MgSO_4$ (5.7 g) was added and the mixture was stirred for another 20 minutes. The white suspension was filtered through a pad of celite and the filter was thoroughly washed with Et_2O. Concentration under reduced pressure gave a slightly yellowish oil of amino ketal **22** (2.42 g, 11.51 mmol, 84%) which was used without further purification.

^{1}H-NMR (400 MHz, $CDCl_3$) δ_H [ppm] 5.68 (d, J = 1.6 Hz, 1 H), 4.09–4.00 (m, 2 H), 4.00–3.91 (m, 2 H), 2.70 (t, J = 7.2 Hz, 2 H), 2.15–1.70 (m, 5 H), 1.70–1.55 (m, 5 H), 1.33 (t, J = 12.8 Hz, 1 H), 0.94 (d, J = 4.4 Hz, 3 H).

^{13}C-NMR (125 MHz, $CDCl_3$) δ_C [ppm] 137.0, 127.8, 108.2, 65.3, 64.2, 42.2, 41.9, 33.8, 32.3, 27.4, 26.0, 21.4.

LC-MS (ESI^+) m/z = 212 (100, $M+H^+$), 253 (56, M+ $MeCN+H^+$), 421 (52, $2M+H^+$), 628 (49), 150 (27).

Spectral data is in agreement with literature.[1]

2.8. Synthesis of *N*-desmethyl-*α*-obscurine (5)

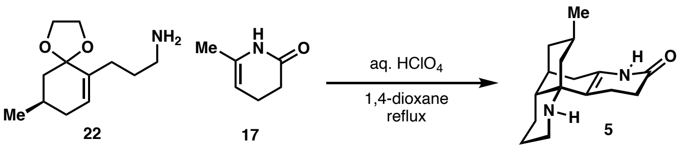

The crude material obtained by following a literature procedure[1] may be purified by a phase-transfer protocol if needed: The yellow residue was taken up in 1 M aq. HCl and washed with CH_2Cl_2 (2 × 50 mL). After adjusting the pH to 6 with sat. aq. Na_2CO_3, another three washes with CH_2Cl_2 followed. Further basification with sat. aq. Na_2CO_3 led to formation of a white precipitate. At pH 13–14, the mixture was extracted again with CH_2Cl_2 (3 × 50 mL). The combined organic extracts from the last step were dried over $MgSO_4$ and concentrated under reduced pressure to afford *N*-desmethyl-*α*-obscurine as yellowish/off-white solid.

^{1}H-NMR (500 MHz, $CDCl_3$) δ_H [ppm] 7.45 (bs, 1H), 2.82 (d, J = 12.5 Hz, 1H), 2.50–2.15 (m, 6H), 1.87 (bs, 1H), 1.71–1.55 (m, 5H), 1.55–1.34 (m, 5H), 1.21 (t, J = 13.5 Hz, 1H), 0.85 (d, J = 6.0 Hz, 3H).

LC-MS (ESI$^+$) *m/z* = 261.2 [$(M+H)^+$].

Spectral data is in agreement with literature.[1]

2.9. Synthesis of *N*-Boc-*α*-obscurine (S3)

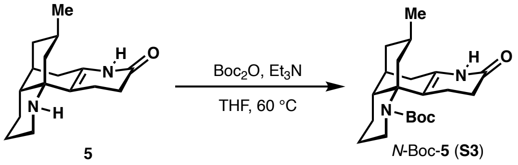

The title compound was prepared from crude **5** according to a literature procedure in 54% yield (over two steps) (lit. 65%).[1]

2.10. Synthesis of *N*-Boc-*β*-obscurine (23)

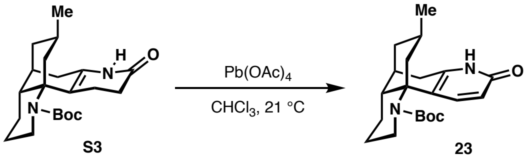

The title compound was prepared according to a literature procedure in 90% yield (lit. 84%).[1]

2.11. Synthesis of 1-triflyl-*N*-Boc-lycodine (24)

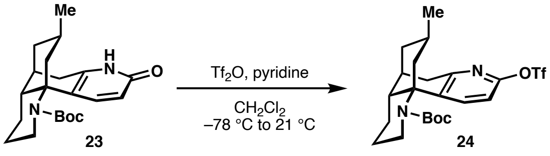

The title compound was prepared according to a literature procedure in 78% yield (lit. 72%).[1]

2.12. Synthesis of 9-oxo-1-triflyl-*N*-Boc-lycodine (25)

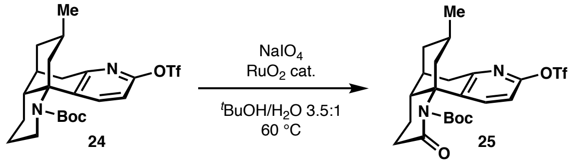

In a 100 mL flask $RuO_2 \cdot H_2O$ (2.2 mg, 16.6 µmol, 1.0 mol%) was suspended in water (23 mL) and $NaIO_4$ (2.41 g, 112.9 mmol, 6.8 equiv) was added to form a clear yellow solution of RuO_4. Triflate **24** (814 mg, 1.66 mmol, 1.0 equiv) dissolved in ^{t}BuOH (3.6 mL) was added to the solution of RuO_4. The source vessel was washed with another 3.6 mL ^{t}BuOH, which was also transferred to the reaction. During the addition, precipitation of black $RuO_2 \cdot H_2O$ was observed, which partially re-dissolved with stirring. The mixture was heated to 60 °C and stirred for 3 h upon which TLC (*n*-hexane/EtOAc 2:1, spot hot reaction mixture) confirmed full conversion of the starting material. After cooling to room temperature, the mixture was diluted with water (50 mL) and extracted with CH_2Cl_2 (3 × 50 mL). The combined organic extracts were dried over $MgSO_4$, concentrated under reduced pressure and the brown residue was passed through a pad of silica (*n*-hexane/EtOAc 2:1 as solvent) to afford triflyl imide **25** (721 mg, 86%) as white solid.

^{1}H-NMR (500 MHz, $CDCl_3$) δ_H [ppm] 8.67 (d, *J* = 8.6 Hz, 1 H), 6.99 (d, *J* = 8.5 Hz, 1 H), 3.14 (dd, *J* = 19.6, 7.5 Hz, 1 H), 2.76 (d, *J* = 19.7, 1 H), 2.53 (ddd, *J* = 18.1, 9.9, 8.3 Hz, 1 H), 2.34 (m, 1 H), 2.24 (ddd, *J* = 17.9, 7.7, 3.0 Hz, 1 H), 2.11 (ddd, *J* = 12.5, 4.7, 3.0 Hz, 1 H), 2.06 (dd, *J* = 12.3, 2.5 Hz, 1 H), 1.84 (d, *J* = 6.2 Hz, 1 H), 1.81 – 1.74 (m, 1 H), 1.56 (s, 9 H), 1.52 – 1.44 (m, 1 H), 1.44 (t, *J* = 12.0 Hz, 1 H), 1.42 – 1.36 (m, 1 H), 1.30 (m, 1 H), 0.88 (d, *J* = 6.2 Hz, 3 H).

^{13}C-NMR (125 MHz, $CDCl_3$) δ_C [ppm] 171.2, 156.9, 154.2, 154.1, 142.2, 136.6, 118.8 (q, *J* (C–F) = 320.4 Hz), 112.8, 84.9, 63.0, 44.8, 42.6, 41.6, 34.6, 32.6, 31.2, 27.7 (2C), 24.8, 22.5, 21.7.

LC-MS (ESI$^+$) *m/z* = 490 (100), 546 (39), 446 (28), 491 (26), 547 (22), 505 (22, M+H$^+$).

IR ν [cm^{-1}] 2927, 1739, 1677, 1419, 1252, 1212, 1140, 955, 932, 881, 848, 600.

$[\alpha]_D^{22}$ = +101.5° (c = 0.93, $CHCl_3$).

HR-MS (ESI+) exact mass calculated for $C_{22}H_{28}N_2O_6F_3S$ [$(M+H)^+$]: *m/z* = 505.1615; found: 505.1620.

2.13. Synthesis of 9-oxo-*N*-Boc-*β*-obscurine (S4)

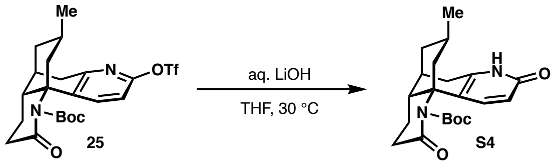

1 M aq. LiOH (3.14 mL, 3.14 mmol, 2.2 equiv) was added to a solution of triflyl imide **25** (720 mg, 1.43 mmol, 1.0 equiv) in THF (7.0 mL). The reaction was heated to 30 °C and stirred for 35 min after which a second portion of 1 M aq. LiOH (2.2 equiv, 4.4 equiv total) was added to the clarified mixture. Stirring was continued for 25 min at 30 °C upon which TLC (hexanes/EtOAc 1:1) showed full conversion of **25**. Water (5 mL) was added and the pH of the aq. phase was carefully adjusted to pH 7.0 with 0.5 M aq. HCl (9.5 mL). The aq. phase was extracted with CH_2Cl_2 (3 × 50 mL), the combined organic extracts were dried over $MgSO_4$ and evaporated under reduced pressure afforded crude pyridonyl imide **S4** as colorless amorphous solid (530 mg). The crude material was taken directly into the pyridone methylation.

^{1}H-NMR (500 MHz, $CDCl_3$) δ_H [ppm] 13.33 (bs, 1H), 8.21 (d, *J* = 9.5 Hz, 1H), 6.45 (d, *J* = 9.5 Hz, 1H), 3.01 (dd, *J* = 19.1, 7.2 Hz, 1H), 2.51 (d, *J* = 19.0 Hz, 1H), 2.54–2.45 (m, 1H), 2.30–2.22 (m, 2H), 2.07 (dd, *J* = 12.7, 3.5 Hz, 1H), 2.05–2.00 (m, 1H), 1.81–1.72 (m, 2H), 1.55 (s, 9H), 1.48–1.39 (m, 1H), 1.37–1.29 (m, 2H), 0.88 (d, *J* = 6.2 Hz, 3H).

^{13}C-NMR (125 MHz, $CDCl_3$) δ_C [ppm] 171.5, 165.1, 154.1, 143.5, 142.7, 118.5, 117.8, 84.6, 61.7, 44.1, 42.2, 41.7, 32.2, 31.0, 29.4, 27.7 (3C), 24.9, 22.2, 21.6.

LC-MS (ESI^+) *m/z* = 414 (100, $M+MeCN+H^+$), 415 (28), 341 (20).

IR ν [cm^{-1}] 2950, 2924, 2870, 1739, 1655, 1612, 1558, 1458, 1368, 1249, 1154, 849.

HR-MS (ESI^+) exact mass calculated for $C_{21}H_{29}N_2O_4$ [$(M+H)^+$]: *m/z* = 373.2122; found 373.2125.

2.14. Synthesis of 1-methoxy-9-oxo-*N*-Boc-lycodine (S5)

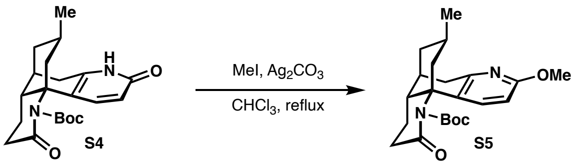

Ag_2CO_3 (749 mg, 2.72 mmol, 1.9 equiv) was added to a solution of crude pyridonyl imide **S4** (530 mg, 1.43 mmol assumed from previous step, 1.0 equiv) in $CHCl_3$ (10 mL) followed by a solution of MeI (111 µL, 1.78 mmol, 2.6 equiv) in $CHCl_3$ (2.0 mL). The grey suspension was heated to reflux (75 °C) in the dark (wrap with tin foil) for 13 hours, after which TLC (*n*-hexane/EtOAc 1:1) showed full conversion of the starting material. After cooling to room temperature, the yellow-brown suspension was filtered through a pad of celite and the yellow oily residue obtained after evaporation was purified by silica gel chromatography (hexanes/EtOAc 2:1) to afford the title compound **S5** as colorless foam (475 mg, 86% over two steps).

^{1}H-NMR (500 MHz, $CDCl_3$) δ_H [ppm] 8.24 (d, *J* = 8.6 Hz, 1H), 6.57 (d, *J* = 8.6 Hz, 1H), 3.88 (s, 3H), 3.07 (dd, *J* = 19.1, 7.5 Hz, 1H), 2.62 (d, *J* = 19.1 Hz, 1H), 2.45 (dt, *J* = 17.3, 8.5 Hz, 1H), 2.30–2.27 (m, 1H), 2.21–2.13 (m, 1H), 2.08–2.01 (m, 2H), 1.83–1.73 (m, 2H), 1.62–1.54 (m, 1H) superimposed on 1.55 (s, 9H), 1.41–1.34 (m, 3H), 0.85 (d, *J* = 4.8 Hz, 3H).

^{13}C-NMR (125 MHz, $CDCl_3$) δ_C [ppm] 171.7, 162.6, 154.0 (d), 139.2, 127.9, 108.7, 84.3, 63.0, 53.5, 45.1, 42.9, 41.8, 34.9, 32.9, 31.0, 27.7 (3C), 24.9, 22.3, 21.7.

LC-MS (ESI$^+$) *m/z* = 387 (100, M+H$^+$), 428 388 (26), (11, M+MeCN+H$^+$).

IR ν [cm^{-1}] 2923, 1740, 1674, 1594, 1476, 1421, 1367, 1312, 1249, 1152, 1031, 850, 731.

HR-MS (ESI) exact mass calculated for $C_{22}H_{31}N_2O_4$ [(M+H)$^+$]: *m/z* = 387.2278; found: 387.2278.

2.15. Synthesis of aminoacid 26

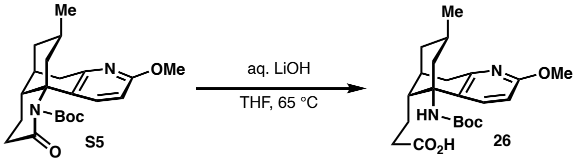

1 M aq. LiOH (5.90 mL, 5.90 mmol, 4.8 equiv) was added to a solution of methoxy imide **S5** (475 mg, 1.23 mmol, 1.0 equiv) in THF (4.5 mL). The mixture was stirred at 65 °C, with an additional portion of 1 M aq. LiOH (1.6 eq, 5.4 equiv total) being added after 6 h. After 22 h, full conversion of the starting material was determined by TLC (*n*-hexane/EtOAc 1:1) and the clear mixture was cooled to room temperature, followed by addition of water (60 mL) and careful acidification with 1 M aq. HCl (ca. 15 mL) to pH 1–2. The aqueous phase was extracted with CH_2Cl_2 (5 × 100 mL) and the combined organic extracts were dried over $MgSO_4$. Removal of solvent under reduced pressure afforded methoxy acid **26** (465 mg, 97%) as a colorless foam without further purification.

^{1}H-NMR (500 MHz, $CDCl_3$) δ_H [ppm] 7.51 (d, *J* = 8.6 Hz, 1H), 6.55 (d, *J* = 8.5 Hz, 1 H), 4.92 (bs, 1H), 3.88 (s, 3H), 3.06 (dd, *J* = 19.0, 7.0 Hz, 1H), 2.52 (d, *J* = 19.0 Hz, 1H), 2.49–2.40 (m, 2H), 2.38–2.29 (m, 3H), 1.83–1.76 (m, 1H), 1.71 (d, *J* = 12.8 Hz, 1H), 1.59–1.55 (m, 1H), 1.45 (s, 9H), 1.39–1.13 (m, 4H), 0.78 (d, *J* = 6.3 Hz, 3H).

^{13}C-NMR (125 MHz, $CDCl_3$) δ_C [ppm] 178.8, 162.3, 154.9 (2C), 135.4, 127.5, 108.6, 79.6, 59.0, 53.5, 47.0, 42.8, 41.1, 34.2, 32.2, 30.8, 28.5 (3C), 26.4, 23.2, 21.9.

LC-MS (ESI^+) *m/z* = 405 (100, $M+H^+$), 406 (29).

IR ν [cm^{-1}] 3500–3000, 2950, 2918, 2868, 1712, 1598, 1578, 1476, 1422, 1312, 1259, 1163, 1035.

HR-MS (ESI+) exact mass calculated for $C_{22}H_{33}N_2O_5$ [$(M+H)^+$]: *m/z* = 405.2384; found 405.2383.

2.16. Synthesis of 1′-methyl-*N*-Boc-casuarinine H (27)

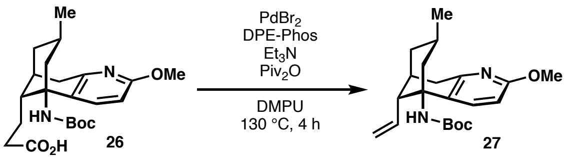

An oven-dried, tall tube with stir-bar and septum cap was loaded with methoxy acid **27** (100 mg, 247 µmol, 1.0 equiv), $PdBr_2$ (2.0 mg, 7.4 µmol, 3 mol%) and DPE-Phos (12.0 mg, 22.3 µmol, 9 mol%) and subjected to three cycles of evacuation and backfilling with nitrogen. Anhydrous DMPU (1.2 mL) was added, followed by pivalic anhydride (105 µL, 519 µmol, 2.1 equiv) and anhydrous triethylamine (4.0 µL, 29.7 µmol, 12 mol%). The clear yellow solution was stirred at 130 °C for 4 h upon which the color changes to orange/red and then dark purple and TLC (*n*-hexane/EtOAc 2:1, *p*-anisaldehyde) confirmed full conversion of the starting material. The solution was cooled to room temperature, then cooled further to 0 °C, and 1 mL saturated aqueous $NaHCO_3$ was added. The solution was allowed to warm to room temperature and stirred for 20 minutes. The mixture was diluted with EtOAc (30 mL) and washed with sat. aq. NH_4Cl (30 mL), water (2 × 30 mL) and brine (30 mL). The organic phase was dried over $MgSO_4$, filtered, and concentrated under reduced pressure to give a purple oil, which was purified by silica gel chromatography (*n*-hexane/EtOAc 9:1) to afford olefin **27** as a colorless oil (57.6 mg, 65%).

^{1}H-NMR (500 MHz, $CDCl_3$) δ_H [ppm] 7.61 (d, *J* = 8.6 Hz, 1 H), 6.57 (d, *J* = 8.6 Hz, 1 H), 5.64 (dt, *J* = 16.9, 10.0 Hz, 1 H), 5.24 (dd, *J* = 16.9, 2.2 Hz, 1 H), 5.07 (dd, *J* = 10.2, 2.2 Hz, 1 H), 4.83 (s, 1 H), 3.90 (s, 3 H), 3.16 (dd, *J* = 18.9, 7.1 Hz, 1 H), 2.96 (d, *J* = 9.7 Hz, 1 H), 2.58 (d, *J* = 18.9 Hz, 1 H), 2.30 (dd, *J* = 7.1, 3.6 Hz, 1 H), 2.16 (d, *J* = 12.2 Hz, 1 H), 1.89 (d, *J* = 12.7 Hz, 1 H), 1.71 (d, *J* = 13.2 Hz, 1 H), 1.44 (s, 9 H), 1.28 (m, 1 H), 1.38 (td, *J* = 13.0, 4.2 Hz, 1 H), 0.82 (d, *J* = 6.4 Hz, 3 H).

^{13}C-NMR (125 MHz, $CDCl_3$) δ_C [ppm] 162.4, 155.4, 154.8, 138.2, 136.2, 128.2, 118.3, 108.6, 79.2, 57.9, 53.5, 49.2, 46.3, 42.7, 35.1, 34.7, 28.6 (3C), 26.4, 22.0.

LC-MS (ESI^+) *m/z* = 359 (100, $M+H^+$), 360 (29), 400 (15, $M+MeCN+H^+$).

IR ν [cm^{-1}] 3500 – 3300, 3071, 2922, 2854, 1724, 1597, 1577, 1476, 1421, 1365, 1314, 1264, 1164, 1034.

HR-MS (ESI^+) exact mass calculated for $C_{21}H_{31}N_2O_3$ [$(M+H)^+$]: *m/z* = 359.2329; found: 359.2329.

2.17. Synthesis of (–)-casuarinine H (2)

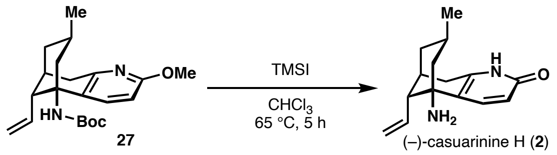

Methoxy olefin **27** (10 mg, 0.028 mmol, 1.0 equiv) was concentrated in a 1 dram vial and introduced to a glovebox. In the glovebox, **27** was dissolved in anhydrous $CHCl_3$ (0.5 mL) and trimethylsilyl iodide (TMSI) (40 µL, 0.28 mmol, 10 equiv) was then added dropwise. The vial was sealed, removed from the glovebox, and heated at 65 °C for 5 hours, resulting in a dark yellow solution. After cooling to room temperature, the reaction was transferred to a 2 dram vial with CH_2Cl_2 (2 × 1.0 mL). The solution was cooled to 0 °C and H_2O, sat. aq. K_2CO_3, and sat. aq. $Na_2S_2O_3$ (0.5 mL of each) were added while stirring. The pH of the resulting solution was confirmed to be >10 and the aqueous phase was extracted with CH_2Cl_2 (3 × 1 mL). The combined organic extracts were dried over $MgSO_4$ and filtered. Concentration under reduced pressure afforded (–)-casuarinine H (**2**) as an off-white solid/foam (6.1 mg, 89%).

^{1}H-NMR (500 MHz, $CDCl_3$) δ_H [ppm] 13.25 (br s, 1H, NH), 7.76 (d, *J* = 9.4 Hz, 1H), 6.43 (d, *J* = 9.3 Hz, 1H), 5.62 (ddd, *J* = 16.9, 9.8, 9.8 Hz, 1H), 5.23 (dd, *J* = 17.0, 2.1 Hz, 1H), 5.13 (dd, *J* = 10.2, 2.1 Hz, 1H), 3.06 (dd, *J* = 18.9, 7.1 Hz, 1H), 2.51 (d, *J* = 18.8 Hz, 1H), 2.29 (m, 1H), 2.15 (dd, *J* = 9.0, 2.5 Hz, 1H), 1.78-1.60 (br s, 2H, NH_2), 1.74 (d, *J* = 13.3 Hz, 1H), 1.65 (dd, *J* = 11.7, 3.6 Hz, 1H), 1.41 (m, 1H), 1.29 (ddd, *J* = 12.8, 12.8, 4.1 Hz, 1H), 1.10 (dd, *J* = 12.1, 12.1 Hz, 1H), 0.85 (d, *J* = 6.4 Hz, 3H).

^{13}C-NMR (151 MHz, $CDCl_3$) δ_C [ppm] 165.0, 143.9, 140.4, 137.4, 120.5, 119.2, 117.3, 54.8, 52.4, 50.0, 42.6, 34.3, 30.0, 26.5, 21.9.

LC-MS (ESI$^+$) *m/z* = 286 (100, M+MeCN+H$^+$), 228 (73, $[M-NH_2]^+$), 287 (23), 245 (22, M+H$^+$), 269 (18), 229 (13).

$[\alpha]_D^{22}$ = –7.1° (c = 0.22, $CHCl_3$).

IR ν [cm^{-1}] 3600–3200, 2924, 2853, 1654, 1609, 1559, 1457, 1178, 1128, 666.

HR-MS (ESI+) exact mass calculated for $C_{15}H_{21}N_2O$ $[(M+H)^+]$: *m/z* = 245.1648; found: 245.1648.

Characterization data in agreement with material isolated from natural sources.[7]

2.18. Synthesis of (+)-lycoplatyrine B (4)

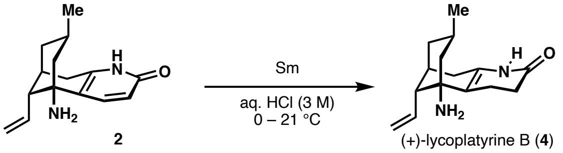

Casuarinine H (**2**, 5.2 mg, 21.3 μmol, 1.0 equiv) was concentrated in a 1 dram vial, cooled to 0 °C, and subsequently dissolved in aq. HCl (3 M, 285 μL). To the clear solution stirring at 0 °C was piecewise added grains of samarium metal (26 mg, 170.3 μmol, 8.0 equiv, 6–7 grains). Complete dissolution of each grain of Sm was observed before the addition of the next grain. The addition of larger grains and/or several of the last grains resulted in a deep purple solution. In these instances, the solution was stirred until the purple color disappeared to yield a clear or very light yellow solution before the addition of the next grain of Sm. *Note: Slow addition of the Sm metal in this manner is critical to avoid an exotherm and to obtain clean product.* Upon complete addition of the Sm metal, full dissolution of the last grain, and decolorization, the solution was stirred for 2 minutes at 0 °C. The ice bath was removed and the solution was stirred for 10 minutes at 21 °C. To the solution was added H_2O (750 μL) and the aqueous layer was washed with MTBE (3 × 750 μL). These organic washes were discarded. The aqueous layer was transferred to a 2 dram vial with H_2O (2 × 500 μL). This solution was cooled to 0 °C and basified via the slow dropwise addition of sat. aq. K_2CO_3 (1.6 mL) with stirring (the solution bubbles vigorously!). The pH was confirmed to be >10. The resulting solution was extracted with MTBE (3 × 1 mL), the extracts dried over $MgSO_4$ and filtered, and the solvent removed under reduced pressure to yield the title compound lycoplatyrine B (**4**) as a white solid (4.4 mg, 84% yield). $CDCl_3$ was deacidified by passing through a plug of basic alumina (Brockmann I) before dissolving the product for NMR analysis.

^{1}H-NMR (600 MHz, $CDCl_3$) δ_H [ppm] 7.17 (br s, 1H, NH), 5.81 (dt, *J* = 17.1, 9.8 Hz, 1H), 5.21 (dd, *J* = 17.1, 2.1 Hz, 1H), 5.13 (dd, *J* = 10.2, 2.2 Hz, 1H), 2.48–2.40 (m, 4H), 2.27 (m, 1H), 2.13 (m, 1H), 2.04 (dd, 9.4, 3.0 Hz, 1H), 1.71 (d, *J* = 18.1 Hz, 1H), 1.68–1.64 (m, 3H), 1.36–1.19 (br s, 2H, NH_2), 1.25–1.20 (m, 1H), 0.89 (d, *J* = 6.1 Hz, 3H), 0.89 (m, 1H).

^{13}C-NMR (151 MHz, $CDCl_3$) δ_C [ppm] 171.2, 138.4, 129.6, 118.3, 114.2, 55.0, 53.0, 47.1, 43.0, 34.6, 31.2, 30.2, 27.0, 22.0, 19.9.

LC-MS (ESI^+) *m/z* = 288 (43, M+MeCN+H^+), 247 (8, M+H^+).

$[\alpha]_D^{22}$ = +79.2° (c = 0.22, CH_2Cl_2).

IR ν [cm^{-1}] 3217-3073, 2948, 2908, 2840, 1666, 1381, 1212, 915, 828.

HR-MS (ESI+) exact mass calculated for $C_{15}H_{23}N_2O$ [$(M+H)^+$]: *m/z* = 247.1805; found: 247.1805. Characterization data in agreement with material isolated from natural sources.[8]

2.19. Synthesis of 1′-methyl-*N*-Boc-8,15-dihydrohuperzine A (S6)

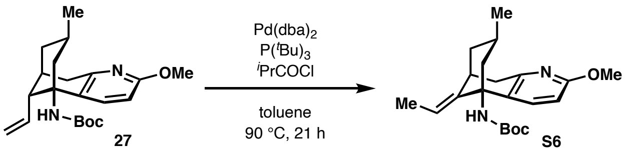

Methoxy terminal olefin **27** (20.5 mg, 57.2 µmol, 1.0 equiv) was concentrated in a 1 dram vial and taken into a glovebox. To the vial was added Pd(dba)$_2$ (6.6 mg, 11.4 µmol, 0.2 equiv) and P(^{t}Bu)$_3$ (2.3 mg, 11.4 µmol, 0.2 equiv), followed by anhydrous toluene (250 µL). In the glovebox in a separate vial, a stock solution of ^{i}PrCOCl (12 µL) in anhydrous toluene (500 µL) was prepared. A 50 µL aliquot of the ^{i}PrCOCl stock solution (containing 1.2 µL, 11.4 µmol, 0.2 equiv ^{i}PrCOCl) was added to the other reagents. The vial was capped with a solid cap, removed from the glovebox, and heated at 90 °C for 21 hours, yielding an orange solution. After cooling to 21 °C, the solution was filtered through a plug of Celite with CH_2Cl_2 and concentrated in vacuo. The crude oil was purified via silica gel chromatography (9:1 *n*-hexane/EtOAc) to yield methoxy internal olefin **S6** (TLC: R_f = 0.39 in 4:1 hexanes/EtOAc, visualized by UV/*p*-anisaldehyde) as a pale-yellow foam (16.5 mg, 81% yield). The product was isolated as an approximate 7:3 mixture of rotamers.

^{1}H-NMR (600 MHz, $CDCl_3$) δ_H [ppm] 7.51 (d, *J* =8.8 Hz, 0.3H, minor rotamer), 7.47 (d, *J* = 8.5 Hz, 0.7H, major rotamer), 6.53 (d, *J* = 8.5 Hz, 1H), 5.38 (m, 1H), 4.74 (s, 0.7H major rotamer), 4.58 (s, 0.3H, minor rotamer), 3.87 (s, 3H), 3.34 (br s, 1H), 3.20 (dd, *J* = 18.1, 7.0 Hz, 0.7H, major rotamer), 3.06 (dd, *J* = 18.5, 6.3 Hz, 0.3H, minor rotamer), 2.78 (d, *J* = 18.1 Hz, 1H), 1.82 (m, 1H), 1.70 (d, *J* = 6.8 Hz, 3H), 1.56 (d, *J* = 14.0 Hz, 1H), 1.52 (m, 1H), 1.47 (d, *J* = 14.4 Hz, 1H), 1.42 (s, 6H, major rotamer), 1.25 (m, 1H), 1.05 (s, 3H, minor rotamer), 0.78 (d, *J* = 6.2 Hz, 3H).

^{13}C-NMR (151 MHz, $CDCl_3$, peaks for major rotamer tabulated) δ_C [ppm] 162.2, 154.5, 153.7, 138.8, 135.2, 129.0, 112.3, 108.2, 79.3, 59.8, 53.5, 52.1, 43.5, 39.4, 31.6, 28.5, 26.1, 21.5, 12.6.

LC-MS (ESI$^+$) *m/z* = 359 (100, M+H$^+$), 400 (6, M+MeCN+H$^+$).

$[\alpha]_D^{22}$ = +15.2° (c = 0.21, CH_2Cl_2).

IR ν [cm^{-1}] 3350-3270, 2921, 2951, 1694, 1598, 1475, 1365, 1311, 1265, 1250, 1168, 1039, 1027, 825.

HR-MS (ESI+) exact mass calculated for $C_{21}H_{31}N_2O_3$ [(M+H)$^+$]: *m/z* = 359.2329; found: 359.2336.

2.20. Synthesis of (–)-8,15-dihydrohuperzine A (3)

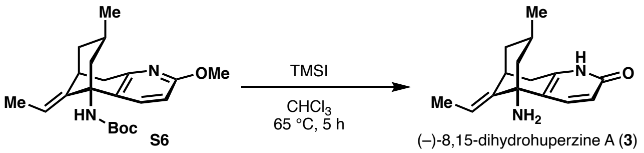

Methoxy internal olefin **S6** (10.4 mg, 0.029 mmol, 1.0 equiv) was concentrated in a 1 dram vial and introduced to a glovebox. In the glovebox, **S6** was dissolved in anhydrous $CHCl_3$ (0.5 mL) and TMSI (42 µL, 0.29 mmol, 10.0 equiv) was then added dropwise. The vial was sealed, removed from the glovebox, and heated at 65 °C for 5 hours, resulting in a dark red/brown solution. After cooling to room temperature, the reaction was transferred to a 2 dram vial with CH_2Cl_2 (2 x 1.0 mL). The solution was cooled to 0 °C and H_2O (1 mL) was added dropwise while stirring. The aqueous phase was washed with MTBE (3 × 2 mL) and the organic washes were discarded. The aqueous layer was cooled to 0 °C and basified via the dropwise addition of sat. aq. K_2CO_3 (800 µL) with stirring. The pH was confirmed to be >10. The resulting solution was extracted with MTBE (3 × 1 mL), the extracts dried over $MgSO_4$, and the solvent removed under reduced pressure to yield the crude product as a colorless oil. The crude product was purified by silica gel chromatography (CH_2Cl_2/MeOH 10:1) to afford 8,15-dihydrohuperzine A (**3**) (TLC: R_f = 0.43 in 9:1 CH_2Cl_2/MeOH, visualized by UV/$KMnO_4$) as a colorless oil (2.9 mg, 41% yield). $CDCl_3$ was deacidified by passing through a plug of basic alumina (Brockmann I) before dissolving the product for NMR analysis.

^{1}H-NMR (600 MHz, $CDCl_3$) δ_H [ppm] 12.83 (br s, 1H, NH), 7.82 (d, *J* = 9.4 Hz, 1H), 6.41 (d, *J* = 9.4 Hz, 1H), 5.50 (q, *J* = 6.7 Hz, 1H), 3.33 (m, 1H), 3.01 (dd, *J* = 18.2, 7.3, Hz, 1H), 2.69 (d, *J* = 18.1 Hz, 1H), 1.80 (d, *J* = 13.2 Hz, 1H), 1.72 (br d, *J* = 12.2 Hz, 1H), 1.67 (d, *J* = 6.7 Hz, 3H), 1.64-1.53 (br s, 2H, NH_2), 1.63–1.56 (m, 1H), 1.19 (dt, *J* = 12.8, 4.4 Hz, 1H), 1.05 (t, *J* = 11.9 Hz, 1H), 0.82 (d, *J* = 6.5 Hz, 3H).

^{13}C-NMR (151 MHz, $CDCl_3$) δ_C [ppm] 164.9, 144.6, 144.4, 139.6, 122.3, 117.1, 111.4, 55.1, 51.3, 42.8, 34.6, 30.7, 26.6, 21.5, 12.4.

LC-MS (ESI^+) *m/z* = 286 (100, M+MeCN+H^+), 245 (5, M+H^+).

$[\alpha]_D^{22}$ = –56.6° (c = 0.15, CH_2Cl_2).

IR ν [cm^{-1}] 3400-3250, 2913, 1654, 1615, 1458, 833, 660.

HR-MS (ESI+) exact mass calculated for $C_{15}H_{21}N_2O$ [$(M+H)^+$]: *m/z* = 245.1648; found: 245.1647. Characterization data in agreement with material isolated from natural sources.[9, 10]

2.21. Synthesis of *N*-Boc-lycodine (30)

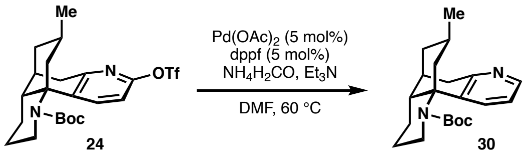

The title compound was prepared from **24** according to a literature procedure in 99% yield (lit. 90%).[1]

2.22. Synthesis of *N*-Boc lycodine boronic ester (S7)

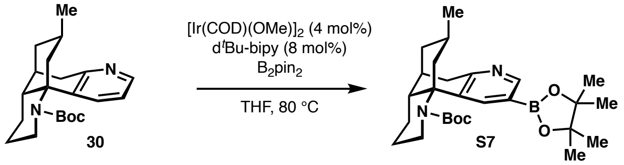

The title compound was prepared from **30** according to a literature procedure[1] and was used without purification in the next step.

2.23. Synthesis of *N*-Boc-2-bromolycodine (31)

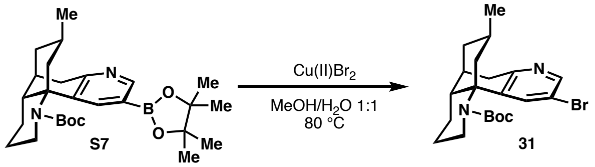

Adapted from a literature procedure.[11] To a solution of crude boronic ester **S7** (0.512 mmol assumed from previous step, 1.0 equiv) in MeOH (25 mL), a solution of copper(II)bromide (241 mg, 1.79 mmol, 3.5 equiv) in H_2O (25 mL) was added. The reaction mixture was heated to reflux (80 °C) for 3 h. Then, the mixture was allowed to cool to r.t. and was quenched with 10% NH_4OH solution (150 mL), followed by extraction with Et_2O (3 × 50 mL). The combined organic layers were washed with H_2O and brine (50 mL), dried over $MgSO_4$ and the solvent was removed under reduced pressure. The resulting black oil was purified by flash column chromatography (*n*-hexane/EtOAc 9:1–4:1–2:1) to afford the title compound **31** as slightly yellow foam (159 mg, 377 µmol, 74% over two steps).

^{1}H NMR (500 MHz, $CDCl_3$) δ_H [ppm] 8.44 (d, *J* = 2.3 Hz, 1H), 7.66 (d, *J* = 2.3 Hz, 1H), 4.11 (dq, *J* = 13.7, 3.1 Hz, 1H), 3.13 (dd, *J* = 19.0, 7.4 Hz, 1H), 2.73 (ddd, *J* = 13.2, 3.7, 1.5 Hz, 1H), 2.63 (d, *J* = 19.0 Hz, 1H), 2.43–2.36 (m, 1H), 2.15–2.11 (m, 1H), 1.89–1.81 (m, 2H), 1.75–1.68 (m, 1H), 1.63–1.55 (m, 2H), 1.55–1.48 (m, 1H) superimposed on 1.51 (s, 9H), 1.42–1.20 (m, 2H), 1.21–1.11 (m, 1H), 0.84 (d, *J* = 6.4 Hz, 3H).

^{13}C-NMR (125 MHz, $CDCl_3$) δ_H [ppm] 156.7, 156.2, 148.6, 137.8, 136.4, 118.5, 80.2, 64.0, 48.4, 44.4, 43.5, 43.0, 34.8, 34.3, 28.7 (3C), 27.8, 26.7, 25.7, 22.5.

LC-MS (ESI^+) *m/z* = 464 (100, $M+MeCN+H^+$), 462 (93), 423 (86, $M+H^+$), 421 (76, $M+H^+$), 465 (23), 424 (19).

$[\alpha]_D^{22}$ = +126.7° (c = 0.255, CH_2Cl_2).

IR ν [cm^{-1}] 2925, 2869, 1700, 1456, 1365, 1268, 1156, 965.

HR-MS (ESI+) exact mass calculated for $C_{21}H_{30}BrN_2O_2$ [$(M+H)^+$]: *m/z* = 421.1485; found: 421.1493.

Spectral data in agreement with those reported for a *N*-Cbz-protected derivative.[11]

2.24. Synthesis of protected piperidine adduct 33

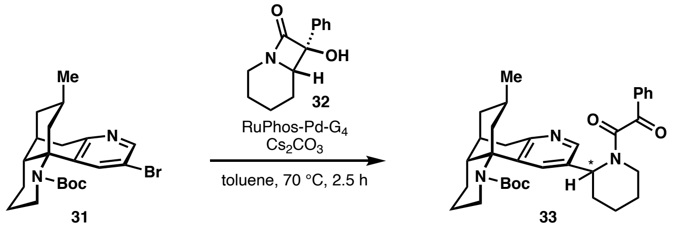

Lactam **32** was prepared according to literature procedures.[12]

In a Schlenk-flask equipped with septum and stir bar, *N*-Boc-2-bromolycodine **31** (19.8 mg, 47 μmol, 1.0 equiv) and enantiomerically pure *trans*-α-hydroxy-β-lactam (2'*R*)-**32** (9.7 mg, 44.6 μmol, 0.95 equiv) were subjected to three cycles of evacuation and backfilling with N_2 before introducing the evacuated vessel to a glove box. Cs_2CO_3 (30.6 mg, 94 μmol, 2.0 equiv), RuPhos Pd G4 (4.0 mg, 4.7 μmol, 10 mol%) and toluene (degassed by three cycles of freeze-pump-thaw, 306 μL, 0.2 M) were added to the flask inside the box. The flask with the light brown suspension was sealed and placed in a preheated oil bath at 70 °C. After 2.5 h, LC-MS and TLC (*n*-hexane/EtOAc 1:1, UV/$KMnO_4$ stain) confirmed full consumption of the bromide and the formation of the desired coupled product. The crude reaction mixture was directly applied to preparative TLC for purification (*n*-hexane/EtOAc, 1:2, product $R_{f,product}$ = 0.5, 3 × 1.0 mL of $CHCl_3$/MeOH 20:1 for elution of material from SiO_2) to yield 17.0 mg (65%) of (2'*R*)-**33** as a colorless foam.

Notes: The β-lactam coupling partner is set as limiting component since unreacted material co-elutes with the coupling product. When racemic trans-*α-hydroxy-β-lactam is used, no separation of product epimers on TLC was observed.*

^{1}H NMR (500 MHz, $CDCl_3$, 21 °C) δ_H [ppm] 8.41 (d, *J* = 2.0 Hz, 0.35H, major rotamer), 8.40 (d, *J* = 2.0 Hz, 0.1H, minor rotamer 1), 8.32 (d, *J* = 2.0 Hz, 0.25H, minor rotamer 2), 8.03–7.92 (m, 1.6H), 7.68–759 (m, 1H), 7.59–7.44 (m, 2.7H), 6.18 (d, *J* = 5.4 Hz, 0.1H minor rotamer 1), 6.04 (d, *J* = 4.6 Hz, 0.4H, major rotamer), 4.88 (d, *J* = 3.3 Hz, 0.3H, minor rotamer 2), 4.63 (d, *J* = 13.4 Hz, 0.3H), 4.21–4.05 (m, 1H), 3.63–3.57 (m, 0.3H), 3.42 (d, *J* = 13.5 Hz, 0.6H), 3.27–3.11 (m, 1H), 3.06 (ddd, *J* = 14.2, 113, 4.0 Hz, 0.5H), 2.90–2.74 (m, 1.3H), 2.69 (t, *J* = 18.7 Hz, 1H), 2.49–2.24 (m, 2H), 2.17–2.09 (m, 1H), 2.20–2.10 (m, 1H), 2.10–1.99 (m, 1H), 1.99–1.67 (m, 6H), 1.67–1.51 (m, 5H), 1.49–1.48 (2 × s, 9H), 1.40–1.11 (m, 4H), 0.88–0.78 (2 × d, *J* = 6.4 Hz, 3H).

^{13}C NMR (125 MHz, $CDCl_3$, 21 °C) δ_C [ppm] 191.50, 191.40, 166.75, 166.50, 157.13, 156.87, 156.47, 156.41, 146.83, 135.52, 135.36, 134.92, 134.89, 133.29, 133.25, 132.64, 132.56, 132.09, 131.15, 129.77, 129.32, 129.23, 79.99, 79.87, 64.07, 63.87, 54.75, 48.96, 48.33, 48.15, 44.34, 44.32, 43.91, 43.81, 43.17, 43.13, 37.82, 37.81, 34.85, 34.81, 34.44, 34.42, 31.06, 28.67 (3C, minor rotamer 2), 28.64 (3C, major rotamer), 27.76, 27.74, 26.69, 26.56, 25.99, 25.60, 25.80, 22.52, 19.78, 19.62.

LC-MS see 2'*S* epimer.

$[\alpha]_D^{22}$ = +115° (c = 1.13, $CHCl_3$).

IR see 2'*S* epimer.

HR-MS (ESI+) exact mass calculated for $C_{34}H_{44}N_3O_4$ [(M+H)$^+$]: *m/z* = 558.3326; found: 558.3379.

(2'*S*)-**33** (13.3 mg, 65% yield) was obtained from a coupling of (2'*S*)-**32** and **31** (15.5 mg, 37 μmol) with adjusted reagent/catalyst amounts.

^{1}H-NMR (500 MHz, $CDCl_3$) δ_H [ppm] 8.42 (d, *J* = 1.8 Hz, 0.4H, major rotamer), 8.40 (d, *J* = 2.0 Hz, 0.1H, minor rotamer 1), 8.32 (d, *J* = 1.8 Hz, 0.25H, minor rotamer 2), 7.98 (d, ^3J = 7.5 Hz, 1.4H), 7.95 (d, *J* = 7.6 Hz, 0.25H), 7.65 (t, *J* = 7.4 Hz, 0.5H), 7.62–7.44 (m, 3H), 6.14 (d, *J* = 5.4 Hz, 0.1H minor rotamer 1), 6.04 (d, *J* = 4.9 Hz, 0.4H, major rotamer), 4.90 (d, *J* = 3.6 Hz, 0.25H, minor rotamer 2), 4.63 (d, ^3J = 13.4 Hz, 0.4H), 4.17–4.06 (m, 1H), 3.69–3.55 (m, 0.4H), 3.41 (d, *J* = 14.0 Hz, 0.6H), 3.26–3.10 (m, 1H), 3.08–3.00 (m, 0.5H), 2.83 (dd, *J* = 13.1, 2.8 Hz, 0.7H), 2.79–2.59 (m, 1.4H), 2.46–2.24 (m, 2H), 2.17–2.09 (m, 1H), 2.09–1.93 (m, 1H), 1.92–1.66 (m, 5H), 1.65–1.50 (m, 5H), 1.52–1.46 (2 × s, 9H), 1.41–1.07 (m, 5H), 0.88–0.78 (2 × d, *J* = 6.4 Hz, 3H).

^{13}C-NMR (125 MHz, $CDCl_3$) δ_C [ppm] 191.58, 191.48, 166.78, 166.70, 157.14, 156.94, 156.45, 156.27, 146.59, 146.21, 135.65, 135.58, 134.92, 134.86, 133.28, 133.13, 132.74, 132.48, 132.17, 131.38, 129.92, 129.75, 129.34, 129.25, 79.97, 79.95, 64.03, 63.91, 54.75, 48.97, 48.49, 48.20, 44.32, 44.30, 43.87, 43.71, 43.17, 43.12, 43.07, 37.79, 34.89, 34.77, 34.43, 34.36, 31.06, 28.70 (3C, minor rotamer 2), 28.64 (3C, major rotamer), 27.79, 27.72, 27.17, 26.67, 26.61, 25.99, 25.78, 25.55, 22.52, 19.81, 19.67.

LC-MS (ESI$^+$) *m/z* = 558.4 [100, $(M+H)^+$], 599.3 [60, $(M+MeCN+H)^+$].

$[\alpha]_D^{22}$ = + 16° (c = 1.33, $CHCl_3$).

IR ν [cm^{-1}] 2924, 1676, 1643, 1444, 1364, 1270, 1222, 1154, 969, 724 (sample consisted of a 1:1 mixture of epimers).

HR-MS (ESI+) exact mass calculated for $C_{34}H_{44}N_3O_4$ [$(M+H)^+$]: *m/z* = 558.3326; found: 558.3368.

Coupling of **31** (68.3 mg, 162 μmol) and *rac*-**32** with adjusted reagent/catalyst amounts yielded **33** as a mixture of epimers at C2' (64.7 mg, 72% yield).

2.25. Synthesis of lycoplatyrine A (8)

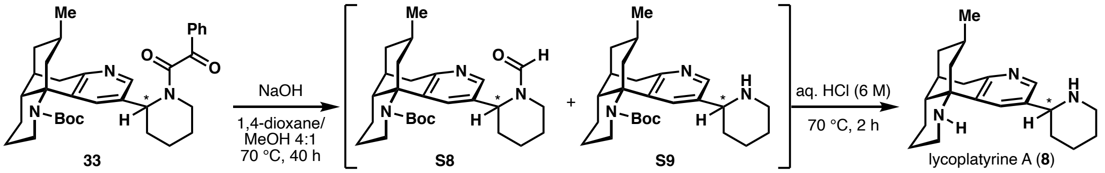

Coupling product **33** (1.0 equiv., 9–13 μmol) and powdered anhydrous NaOH (5.0 equiv) were provided in a 4 mL threaded glass vial equipped with a stir bar. MeOH (600 μL) and 1,4-dioxane (2.4 mL) were added in this order before the vial was sealed with a Teflon-lined cap and placed in a preheated aluminum heat block at 70 °C. After 18 h, another portion of NaOH was added to the now grey suspension (1 equiv) and stirring at 70 °C was continued for 2 h upon which TLC (Et_2O/MeOH 9:1, UV/$KMnO_4$) and LC-MS confirmed virtually full conversion of the starting material. The reaction mixture was cooled to 21 °C and transferred dropwise to a separation funnel with aq. HCl (0.2 M, 5 mL). The acidic colorless, clear solution was washed with Et_2O (2 × 5 mL) before it was basified to a pH > 10 with aq NaOH (15%). The emulsion was extracted with Et_2O (3 × 5 mL), the combined extracts dried over $MgSO_4$ and the solvent removed under reduced pressure to give the free piperidine **S9** along with a minor amount of the piperidine-*N*-formamide **S8** as a colorless foam.

Characterization for C2' epimeric mixture:

^{1}H NMR (500 MHz, $CDCl_3$, 21 °C) δ_H [ppm] (peaks that could be unambiguously assigned to one of the compounds through comparison with a spectrum of a purified sample of **S8** are indicated) 8.38 (dd, *J* = 13.6, 2.0 Hz, 0.6H, **S9**), 8.29 (dd, *J* = 15.7, 2.0 Hz, 0.2H, **S8**), 8.26 (bs, 0.2H, **S8**), 8.21 (s, 0.1H, 8.20, 0.10H, **S8**), 8.15 (s, 0.1H, **S8**), 7.47 (dd, *J* = 21.7, 2.0 Hz, 0.5H, **S9**), 7.45 (dd, *J* = 19.8, 2.0 Hz, 0.2H, **S8**), 7.37 (dd, *J* = 12.4, 1.6 Hz, 0.3H, **S8**), 5.77–5.72 (m, 0.3H, **S8**), 4.78–4.74 (m, 0.2H, **S8**), 4.15–4.07 (m, **S8**) superimposed on 4.07 (d, *J* = 13 Hz, **S9**, 1.2H total), 3.58–3.53 (m, 0.6H, **S9**), 3.50–3.44 (m, 0.3H, **S8**), 3.21–3.12 (m, 1.6H), 3.11–3.02 (m, 0.3H, **S8**), 2.96–2.81 (m, 0.4H, **S8**), 2.81–2.61 (m, 2.4H), 2.41–2.26 (m, 1.4H), 2.15–2.07 (m, 1.1H), 1.95–1.78 (m, 3.4H), 1.78–1.66 (m, 2.8H), 1.66–1.43 (m) superimposed on 1.51 (s, **S9**) and 1.48 (s, **S8**, 15.3H total), 1.36–1.21 (m, 2.2H), 1.21–1.11 (m, 1.0H), 0.84 (dt, *J* = 6.3, 1.8 Hz, **S8**) superimposed on 0.80 (dd, *J* = 6.4 Hz, 2.7 Hz, **S9**, 3.0H total).

^{13}C NMR (125 MHz, $CDCl_3$, 21 °C) δ_C [ppm] (multiplicity of signals within 0.1 ppm are given in parentheses) 161.9, 161.7 (2), 157.5 (2), 156.6 (5), 156.5 (2), 156.4, 146.6 (2), 146.4 (2), 146.3, 139.1 (2), 135.7, 135.5, 135.3 (2), 135.1 (2), 132.7, 132.5, 132.3 (2), 132.2 (4), 132.0, 131.8, 80.0 (3), 79.8 (2), 79.7 (2), 64.3 (2), 64.0, 63.9 (3), 60.0, 59.8, 55.5 (2), 48.5 (3), 48.3, 48.1 (3), 47.8 (2), 44.3 (5), 44.1, 43.8 (3), 43.2 (3), 37.9 (2), 34.9 (5), 34.5 (5), 29.8, 29.6, 28.7 (3), 27.7 (3), 27.4 (2), 26.7 (3), 26.5 (3), 26.0 (2), 25.8 (4), 25.4 (2), 25.1 (2), 22.5 (2), 20.8 (2), 20.1 (2).

The mixture of starting materials (**S8**, **S9**) from the previous step was taken up in aq. HCl (6 M, 300 μL) and stirred for 2 h at 70 °C in a preheated oil-bath upon which LC-MS confirmed full conversion to the starting materials. The reaction was cooled to 21 °C and washed with CH_2Cl_2 (2 × 500 μL). The vial with the aqueous phase was then placed in a water bath and NH_4OH (35%, 1.0 mL) was added dropwise whilst stirring until the pH was >10. The basified aqueous layer was extracted with CH_2Cl_2 (3 × 500 μL), the organic extracts were dried over $MgSO_4$ and the solvent removed under reduced pressure to give lycoplatyrine A (**8**) as colorless oil.

Deprotection of (2'S)-**33** (12.7 µmol) yielded (2'S)-**8** (3.7 mg, 11.4 µmol, 90% yield over two steps) in >95:5 *d.r.* (^{1}H-NMR).

^{1}H NMR (500 MHz, $CDCl_3$) δ_H [ppm] 8.32 (d, ^{4}J = 2.1 Hz, 1H), 7.74 (d, ^{4}J = 2.1 Hz, 1H), 3.61 (d, ^{3}J = 10.5 Hz, 1H); 3.20 (d, J = 11.6 Hz, 1H), 3.13 (dd, ^{2}J = 18.6, ^{3}J = 7.1 Hz, 1H), 2.79 (m, 2H), 2.69 (d, ^{2}J = 18.6 Hz, 1H), 2.42 (m, 1H), 2.08 (m, 1H), 1.90 (m, 1H), 1.82–1.64 (m, 6H), 1.60 (dt, J = 12.4, 2.9 Hz, 1H), 1.56–1.47 (m, 6H), 1.44 (d, J = 10.3 Hz, 1H), 1.33 (td, J = 12.3, 3.8 Hz, 1H), 1.27–1.12 (m, 3H), 0.76 (d, ^{3}J = 6.0 Hz, 3H).

^{13}C NMR (125 MHz, $CDCl_3$) δ_C [ppm] 157.7, 145.9, 138.8, 135.9, 131.1, 60.0, 56.4, 51.5, 47.9, 44.7, 44.0, 41.5, 35.3, 35.1, 33.9, 27.9, 26.3, 25.9 (2C), 25.5, 22.2.

LC-MS (ESI+): *m/z* = 326 [$(M+H)^+$], 367 [$(M+MeCN+H)^+$], 225, 246.

$[\alpha]_D^{20}$= –52.4° ($CHCl_3$, c = 0.37).

IR ν [cm^{-1}] 2924, 1692, 1643, 1444, 1364, 1270, 1222, 1154, 969, 724.

HR-MS (ESI^+) exact mass calculated for $C_{21}H_{32}N_3$ [$(M+H)^+$]: *m/z* = 326.2591; found: 326.2588.

Deprotection of (2'R)-**33** (14.3 µmol) yielded (2'R)-**8** (3.2 mg, 9.8 µmol, 68% yield over two steps) in >95:5 *d.r.* (^{1}H-NMR).

^{1}H-NMR (500 MHz, $CDCl_3$) δ_H [ppm] 8.37 (d, ^{4}J = 2.0 Hz, 1H), 7.72 (d, ^{4}J = 2.0 Hz, 1H), 3.62 (d, ^{3}J = 10.0 Hz, 1H); 3.20 (d, J = 11.5 Hz, 1H), 3.13 (dd, ^{2}J = 18.1, ^{3}J = 7.1 Hz, 1H), 2.83-2.74 (m, 2H), 2.69 (d, ^{2}J = 18.6 Hz, 1H), 2.41 (m, 1H), 2.08 (m, 1H), 1.90 (m, 1H), 1.84–1.64 (m, 6H), 1.61–1.47 (m, 7H), 1.44 (d, J = 10.4 Hz, 1H), 1.33 (td, J = 12.4, 3.8 Hz, 1H), 1.28–1.12 (m, 3H), 0.77 (d, ^{3}J = 6.1 Hz, 3H); 1,4-dioxane (3.70, s) as solvent impurity.

^{13}C-NMR (125 MHz, $CDCl_3$, 21 °C) δ_C [ppm] 157.6, 145.7, 138.8, 135.8, 131.4, 60.1, 56.3, 51.6, 47.9, 44.8, 44.0, 41.6, 35.3, 35.2, 33.9, 28.0, 26.3, 26.0 (2C), 25.5, 22.2.

$[\alpha]_D^{20}$= +17.4° ($CHCl_3$, c = 0.32).

IR ν [cm^{-1}] see 2'S epimer.

LC-MS (ESI+): *m/z* = see 2'S epimer.

HR-MS (ESI^+) exact mass calculated for $C_{21}H_{32}N_3$ [$(M+H)^+$]: *m/z* = 326.2591; found: 326.2576.

Spectral data of both isomers is in agreement with literature.[8]

The natural product derived from deprotection of the coupling product with racemic **32** was obtained as a 1.5:1 mixture of (2'S) and (2'R) epimers, respectively.

2.26. Synthesis of *N*-Boc pyrrolidine adduct 36

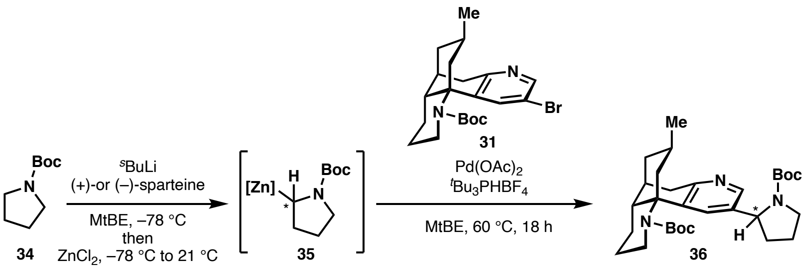

N-Boc-pyrrolidine (**34**, 12.5 µL, 71.2 µmol, 1.2 equiv.) and (–)-sparteine (16.4 µL, 71.2 µmol, 1.2 equiv.) were provided in a threaded tall test tube (l = 15 cm, V = 10 mL) equipped with a stir bar and septum screw cap. Anhydrous MTBE (200 µL) was added and the colorless to slightly yellow solution was cooled to –78 °C (acetone/dry ice bath). ^{s}BuLi (51 µL of a 1.4 M solution in hexanes, 71.2 µmol, 1.2 equiv.) was added dropwise directly (!) into the rapidly stirred solution (*the ^{s}BuLi solution will solidify on the cooled tube wall and upon removal of the ice bath rapid thawing will lead to uncontrolled addition of the reagent*). The first slightly turbid mixture was stirred at –78 °C for 3 h, after which a solution of $ZnCl_2$ (85.4 µL of a 0.5 M solution in THF, 42.7 µmol, 0.72 equiv.) was added dropwise to the now clear, rapidly (!) stirred solution (>1000 rpm). Upon complete addition the mixture was rapidly stirred at –78°C for 30 min, then the ice bath was removed and the mixture was allowed to warm to room temperature in ambient air before it was placed in a water bath at 21 °C and rapid stirring was continued for another 30 min. **31** (23.8 mg, 56.5 µmol, 0.95 equiv.) was provided in an oven-dried glass vial and dissolved in anhydrous MTBE (100 µL). The solution was transferred dropwise to the reaction using a cannula and the source vial is washed with another 100 µL MTBE. Then $Pd(OAc)_2$ (0.7 mg, 3.0 µmol, 5 mol%) and $^tBu_3PHBF_4$ (1.1 mg, 3.6 µmol, 6 mol%) were added together as solids in a N_2-stream. The resulting yellow, clear solution was placed in a preheated oil bath at 60 °C and the precipitation of Zn-salts was observed within the first 30 to 60 min. After 18 h, TLC (*n*-hexane/EtOAc 1:1, UV/$KMnO_4$) and LC-MS confirmed full consumption of **31** and the beige suspension was cooled to room temperature. NH_4OH (35%, 11.5 µL) was added and the beige suspension was stirred for 30 min at 21 °C before filtering through a short pad of celite. The reaction vial and plug were washed with MTBE (3 × 1.0 mL) and the filtrate was evaporated in vacuo to give a yellow oil, which was purified by column chromatography (EtOAc/*n*-hexane 3:1) or preparative TLC (EtOAc/*n*-hexane 1:1) to afford the coupling product (2'*R*)-**36** as colorless foam (25.4 mg, 88%).

^{1}H NMR (500 MHz, $CDCl_3$, 21 °C) δ_H [ppm] 8.24 (s, 1H), 7.35–7.26 (m, 1H), 4.92 (bs, minor rotamer, 0.4H), 4.70 (bs, major rotamer, 0.5H); 4.16–3.98 (m, 1H), 3.66–3.45 (m, 2H), 3.26–3.09 (m, 1H), 2.78 (d, *J* = 13.0 Hz, 1H), 2.67 (d, *J* = 18.6 Hz, 1H), 2.39–2.23 (m, 1H), 2.35 (t, *J* = 12.92, 1H), 2.11 (bs, 1H), 1.97–1.67 (m, 6H), 1.62–1.40 (m, 16H), 1.36–1.13 (m, 8H), 0.82 (d, ^3J = 5.7 Hz, 3H).

^{13}C NMR (125 MHz, $CDCl_3$, 21 °C) δ_C [ppm] (multiplicity of signals within 0.1 ppm are given in parentheses) 156.3 (3), 154.4, 145.6, 138.9, 135.0 (2), 131.2, 130.2, 79.8, 79.6, 64.1, 59.4, 58.7, 48.4, 47.4, 47.2, 44.4, 44.0 (3), 43.2, 36.1, 35.1, 34.9, 34.5, 28.7, 28.6, 28.4, 27.7 (2), 26.7 (2), 25.8, 23.5 (2), 22.5 (2).

LC-MS (ESI$^+$): see 2'*S* epimer. **IR** ν [cm^{-1}] see 2'*S* epimer.

$[\alpha]_D^{22}$ = +82.2° (c = 0.65, $CHCl_3$).

HR-MS (ESI$^+$) exact mass calculated for $C_{30}H_{46}N_3O_4$ [(M+H)$^+$]: *m/z* = 512.3483; found: 512.3516.

(2'*S*)-**36** (16.7 mg, 55% yield) was obtained from *ortho*-lithiation of **34** in the presence of (+)-sparteine and coupling with **31** (24.0 mg, 57 µmol) using adjusted reagent/catalyst amounts.

^{1}H NMR (500 MHz, $CDCl_3$, 21 °C) δ_H [ppm] 8.22 (d, *J* = 2.1 Hz, 1H), 7.29 (d, *J* = 2.2 Hz, 1H), 4.95 (bs, minor rotamer, 0.3 H), 4.71 (bs, major rotamer, 0.6H), 4.15–4.03 (m, 1.3H), 3.67–3.41 (m, 2H), 3.23–3.08 (m, 1H), 2.75–2.59 (m, 2H), 2.41–2.20 (m, 2H), 2.11 (bs, 1H), 1.95–1.65 (m, 6H), 1.62–1.36 (m, 16H), 1.50 (s superimposed on m, N^5-Boc), 1.43 (s superimposed on m, N'-Boc minor rotamer), 1.36–1.08 (m, 10H), 1.17 (s superimposed on m, N'-Boc major rotamer), 0.80 (d, ^3J = 6.4 Hz, 3H).

^{13}C NMR (125 MHz, $CDCl_3$, 21 °C) δ_C [ppm] 156.4 (2), 154.2, 145.9, 139.0, 135.6, 130.3, 80.2, 79.4, 64.2, 60.5, 59.1, 48.7, 47.2, 44.0, 43.7, 43.1, 36.3, 34.9, 34.4, 28.7, 28.6, 28.4, 27.8, 26.8, 25.8, 23.4, 22.4, 21.2.

LC-MS (ESI$^+$): *m/z* = 512 [$(M+H)^+$], 553 [$(M+MeCN+H)^+$].

$[\alpha]_D^{22}$ = +18.3° (c = 0.64, $CHCl_3$).

IR ν [cm^{-1}] 3000–2900, 1697, 1390, 1365, 1157.

HR-MS (ESI$^+$) exact mass calculated for $C_{30}H_{46}N_3O_4$ [$(M+H)^+$]: *m/z* = 512.3483; found: 512.3454

2.27. Synthesis of deprotected pyrrolidine adduct 14

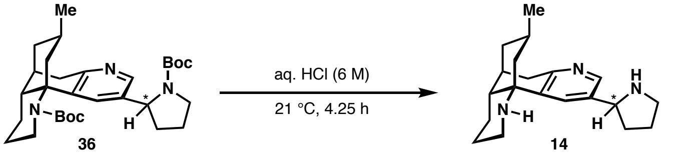

36 14

Coupling product (2'*S*)-**36** (16.7 mg, 32.6 μmol, 1.0 equiv) was taken up in aq. HCl (6 M, 500 μL) and stirred for 4.25 h at 21 °C upon which LC-MS confirmed full consumption of the starting material. The aqueous reaction mix was washed with MTBE (2 × 1 mL) followed by careful adjustment of the pH with NH_4OH (35%) to ≥ 9. The basic aqueous phase was again extracted with MTBE (2 × 1 mL), the extracts were dried over $MgSO_4$ and the solvent evaporated under reduced pressure (40 °C) to yield the free amine (2'*S*)-**14** as colorless oil (10.2 mg, quantitative) in >95:5 *d.r.* (^{1}H-NMR).

^{1}H NMR (500 MHz, $CDCl_3$, 21 °C) δ_H [ppm] 8.34 (d, *J* = 2.2 Hz, 1H), 7.75 (d, *J* = 2.1 Hz, 1H), 4.10 (t, *J* = 7.7 Hz, 1H), 4.24–3.16 (m, 1H), 3.12 (dd, *J* = 18.6, 7.2 Hz, 1H), 3.01 (ddd, *J* = 10.2, 8.4, 6.8 Hz, 1H), 2.78–2.73 (m, 1H), 2.69 (d, *J* = 18.6 Hz, 1H), 2.46–2.37 (m, 1H), 2.23–2.15 (m, 1H), 2.07 (dq, *J* = 6.1, 2.8 Hz, 1H), 1.98–1.80 (m, 3H), 1.77 (d, *J* = 12.7 Hz, 2H), 1.71–1.61 (m, 1H), 1.61–1.41 (m, 5H), 1.33 (td, *J* = 12.3, 3.4 Hz, 1H), 1.28–1.10 (m, 4H), 0.76 (d, *J* = 6.2 Hz, 3H).

^{13}C NMR (125 MHz, $CDCl_3$, 21 °C) δ_C [ppm] 157.4, 145.9, 137.9, 135.8, 131.2, 60.5, 56.3, 51.5, 47.1, 44.8, 44.0, 41.5, 35.2, 34.6, 33.9, 28.0, 26.3, 25.9, 25.6, 22.2.

LC-MS (ESI$^+$): *m/z* = 218 (100), 312 (87, M+H$^+$), 353 (91, M+MeCN+H$^+$), 239 (86).

$[\alpha]_D^{22}$ = −34.08 ± 0.17° (c = 3.3, $CHCl_3$).

IR ν [cm^{-1}] 3278, 2914, 1454, 750.

HR-MS (ESI$^+$) exact mass calculated for $C_{20}H_{30}N_3$ [(M+H)$^+$]: *m/z* = 312.2434; found: 312.2480

Deprotection of (2'*R*)-**36** (15.0 mg, 29.3 μmol) under identical conditions yielded (2'*R*)-**14** (6.4 mg, 70%) in >95:5 *d.r.* (^{1}H-NMR).

^{1}H NMR (500 MHz, $CDCl_3$, 21 °C) δ_H [ppm] 8.36 (d, *J* = 1.7 Hz, 1H), 7.74 (d, 1.4 Hz, 1H), 4.10 (t, 7.8 Hz, 1H), 3.24–3.17 (m, 1H), 3.13 (dd, *J* = 18.6, 7.2 Hz, 1H), 3.05–2.97 (m, 1H), 2.77 (bd, *J* = 13.8 Hz, 1H), 2.69 (d, *J* = 18.6 Hz, 1H), 2.42 (td, *J* = 13.3, 12.7, 3.8 Hz, 1H), 2.26–2.16 (m, 1H), 2.11–2.05 (m, 1H), 1.98–1.82 (m, 2H), 1.77 (bd, *J* = 11.7 Hz, 3H), 1.71–1.62 (m, 1H), 1.61–1.48 (m, 4H), 1.45 (bd, *J* = 11.7 Hz, 1H), 1.33 (td, *J* = 12.4, 3.7 Hz, 1H), 1.29–1.12 (m, 5H), 0.77 (d, *J* = 6.1 Hz, 3H).

^{13}C NMR (125 MHz, $CDCl_3$, 21 °C) δ_C [ppm] 157.3, 145.8, 137.9, 135.7, 131.3, 60.6, 56.3, 51.6, 47.1, 44.9, 44.0, 41.6, 35.2, 34.3, 33.9, 28.1, 26.3, 26.0, 25.6, 22.2.

LC-MS (ESI$^+$): see 2'*S* epimer.

$[\alpha]_D^{22}$ = +14.25° (c = 3.2, $CHCl_3$).

IR ν [cm^{-1}] see 2'*S* epimer.

HR-MS (ESI$^+$) exact mass calculated for $C_{20}H_{30}N_3$ [(M+H)$^+$]: *m/z* = 312.2434; found: 312.2483.

2.28. Synthesis of protected amino ester adduct 38

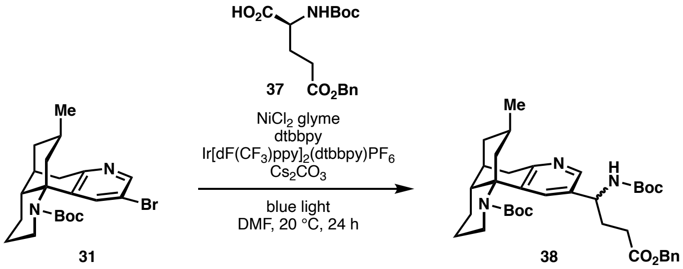

To a 1 dram vial was added *N*-Boc-2-bromolycodine (**31**, 15.0 mg, 35.6 μmol, 1.0 equiv), *N*-Boc-*O*-Bn-glutamic acid (**37**, 18.0 mg, 53.4 μmol, 1.5 equiv), Ir[dF(CF_3)ppy]$_2$(dtbbpy)PF_6 (0.4 mg, 0.4 μmol, 1 mol%) and Cs_2CO_3 (17.4 mg, 53.4 μmol, 1.5 equiv). The vial was evacuated and back-filled with N_2, and anhydrous DMF (1 mL) was added. A stock solution $NiCl_2$ glyme (1 mg, 3.7 μmol) and dtbbpy (2 mg, 7.1 μmol) in anhydrous DMF (950 μL) was prepared. The light blue cloudy solution was sonicated until it became completely clear. An aliquot of the Ni/dtbbpy stock solution (95 μL) was then added to the vial containing the other reactants (delivering 1 mol% $NiCl_2$ glyme and 2 mol% dtbbpy to the reaction). The resulting mixture was sparged with N_2 for 20 min, the septa cap was taped, and the vial was placed in a Merck photoreactor equipped with a 450 nm module (100% intensity, 1000 rpm stir rate, 21 °C set temperature) for 19.5 hours. The resulting brown suspension was diluted with EtOAc (15 mL) and water (15 mL), phases were separated, and the aqueous phase was extracted with EtOAc (2 × 15 mL). The combined organic extracts were washed with brine (20 mL), dried over $MgSO_4$, filtered, and concentrated *in vacuo* to provide a yellow oil. The crude product was purified by column chromatography (0.7 × 10 cm SiO_2, eluted with 40 mL 4:1 hexanes/EtOAc, followed by 50 mL 1:1 hexanes/EtOAc) to yield the product **38** as a yellow oil (15.3 mg, 84% yield). The product is a mixture of epimers as determined by NMR spectroscopy. The epimers can be separated via SiO_2 column if desired.

^{1}H NMR (600 MHz, $CDCl_3$, 21 °C) δ_H [ppm] 8.34 (br s, 1H), 7.53–7.49 (m, 1H), 7.37–7.31 (m, 5H), 5.12–5.11 (m, 2H), 5.03 (br s, 0.5H), 4.97 (br s, 0.5H), 4.71 (br s, 1H, NH), 4.09–4.05 (m, 1H), 3.21 (dd, *J* = 18.9, 7.3 Hz, 1H), 2.75–2.71 (m, 2H), 2.48–2.40 (m, 2H), 2.36–2.28 (m, 1H), 2.14–2.12 (m, 1H), 2.09–2.04 (m, 2H), 1.88–1.82 (m, 2H), 1.73–1.70 (m, 1H), 1.59–1.56 (m, 1H), 1.52–1.51 (m, 9H), 1.48–1.44 (m, 1H), 1.40 (br s, 9H), 1.35–1.30 (m, 2H), 1.26–1.13 (m, 2H), 0.83 (d, *J* = 6.4 Hz, 1.5H), 0.82 (d, *J* = 6.4 Hz, 1.5H).

^{13}C NMR (151 MHz, $CDCl_3$, 21 °C, for peaks where epimers resolve, the chemical shift for the second carbon peak is indicated in parentheses) δ_C [ppm] 172.89 (172.84), 156.60, 156.38 (156.34), 155.18, 144.90, 136.75, 136.37, 135.85 (135.83), 132.50, 128.73 (2C), 128.47 (128.46), 128.44 (128.41, 2C), 80.00, 79.97, 66.73 (66.69), 64.06 (64.02), 52.26 (52.24), 48.34, 44.39 (44.30), 43.67, 43.03 (43.01), 34.30, 31.59, 31.41, 31.22 (31.19), 28.71 (28.70, 3C), 28.45 (3C), 27.69 (27.66), 26.68, 25.71, 22.47.

LC-MS (ESI$^+$): *m/z* = 634 (100, M+H$^+$).

$[\alpha]_D^{22}$ = +63.8° (c = 0.63, CH_2Cl_2).

IR ν [cm^{-1}] 3400, 2974, 2926, 2868, 1701, 1515, 1455, 1389, 1365, 1271, 1251, 1157, 980, 741, 699.

HR-MS (ESI$^+$) exact mass calculated for $C_{37}H_{52}N_3O_6$ [(M+H)$^+$]: *m/z* =634.3851; found: 634.3853.

2.29. Synthesis of lycopladine F (9)

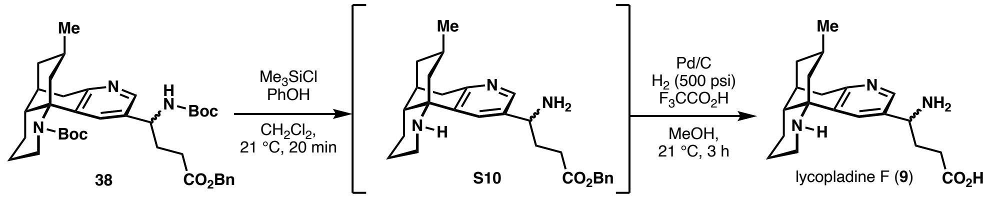

Coupling product **38** (mixture of epimers, 15.3 mg, 24 µmol, 1.0 equiv) was concentrated in a 1 dram vial. Stock solutions of phenol (4 M, 455 µL, 1.8 mmol) and Me_3SiCl (4 M, 153 µL, 0.61 mmol) in anhydrous CH_2Cl_2 were added in this order and the sealed vial was stirred at 21 °C for 20 min. The solution was cooled to 0 °C, aq. HCl (0.5 M, 750 µL) was added and the mixture was washed with MTBE (6 × 1 mL). The pH of the aqueous phase was adjusted to >10 with sat. aq. K_2CO_3 and extracted with MTBE (3 × 1 mL). The organic extracts were dried over $MgSO_4$, filtered, and concentrated in vacuo to afford the crude product **S10** as a clear oil, which was analyzed by NMR spectroscopy and telescoped to the debenzylation without further purification.

^{1}H NMR (600 MHz, $CDCl_3$, 21 °C) δ_H [ppm] 8.32 (d, *J* = 2.2 Hz, 0.5H), 8.28 (d, *J* = 2.3 Hz, 0.5H), 7.74 (d, *J* = 2.3 Hz, 0.5H), 7.70 (d, *J* = 2.3 Hz, 0.5H), 7.37–7.26 (m, 5H), 5.11–5.10 (m, 2H), 3.96–3.92 (m, 1H), 3.13 (dd, *J* = 18.6, 7.3 Hz, 1H), 2.78–2.74 (m, 1H), 2.70 (d, *J* = 18.6 Hz, 1H), 2.41–2.35 (m, 3H), 2.10–2.07 (m, 1H), 2.05–2.00 (m, 2H), 1.79–1.75 (m, 1H), 1.59–1.49 (m, 7H), 1.46–1.43 (m, 1H), 1.36–1.31 (m, 1H), 1.21–1.13 (m, 3H), 0.78 (d, *J* = 6.3 Hz, 1.5H), 0.77 (d, *J* = 6.3 Hz, 1.5H).

^{13}C NMR (151 MHz, $CDCl_3$, 21 °C, for peaks where epimers resolve, the chemical shift for the second carbon peak is indicated in parentheses) δ_C [ppm] 173.27, 157.97 (157.91), 145.70 (145.47), 138.88 (138.81), 136.20, 136.07, 131.37, 130.95, 128.73 (2C), 128.39 (2C), 66.46, 56.29 (56.25), 53.54 (53.35), 51.65 (51.61), 44.90, 43.99, 41.57 (41.54), 35.21, 34.54, 33.94, 31.44 (31.38), 28.13, 26.35, 26.00, 22.22.

In a 1 dram vial, crude benzyl ester **S10** (assuming 24 µmol) and palladium on activated charcoal (0.5 mg, 5% w/w) were taken up in anhydrous MeOH (500 µL), and trifluoroacetic acid (TFA) (2 µL, 24.4 µmol, 1.0 equiv) was added to the suspension. The vial, capped with a septa lid through which a large needle was inserted, was placed in a hydrogenation bomb at 500 psi hydrogen pressure. The suspension was stirred at 21 °C (900 rpm) for 3 h before the pressure was released and the reaction was filtered through a plug of Celite (moistened with MeOH). The plug was washed with 3 × 1.0 mL MeOH and the collected filtrate was evaporated under reduced pressure to yield a 1:1 epimeric mixture at C2' of lycopladine F (**9**) as a white solid (7.8 mg, 71% yield over two steps, calculated as the mono TFA salt) (1:1 mixture of 2'*S* and 2'*R* epimers). $CDCl_3$ was deacidified by passing through a plug of basic alumina (Brockmann I) before dissolving the product for NMR analysis. *Note: Omitting TFA from the debenzylation procedure yielded a product for which the NMR spectra did not match the spectra detailed in the isolation report.*[13]

^{1}H NMR (600 MHz, CD_3OD, 21 °C) δ_H [ppm] 8.61 (d, *J* = 2.0 Hz, 0.5H), 8.58 (d, *J* = 2.0 Hz, 0.5H), 8.25 (d, *J* = 2.1 Hz, 0.5H), 8.17 (d, *J* = 2.1 Hz, 0.5H), 4.50 (m, 1H), 3.29–3.19 (m, 2H), 2.99–2.90 (m, 1H), 2.84 (d, *J* = 19.3 Hz, 0.5H), 2.83 (d, *J* = 19.4 Hz, 0.5H), 2.43–2.29 (m, 5H), 2.09 (br d, *J* = 12.4 Hz, 1H), 1.94–1.84 (m, 4H), 1.74 (br d, *J* = 13.5 Hz, 1H), 1.62 (dd, *J* = 12.0, 12.0 Hz, 1H), 1.47 (ddd, *J* = 13.0, 12.9, 3.4 Hz, 1H), 1.38–1.22 (m, 2H), 0.89 (d, *J* = 6.6 Hz, 1.5H), 0.88 (d, *J* = 6.6 Hz, 1.5H).

^{13}**C NMR** (151 MHz, CD_3OD, 21 °C, for peaks where epimers resolve, the chemical shift for the second carbon peak is indicated in parentheses) δ_C [ppm] 175.24 (175.19), 160.81 (160.76), 149.38 (149.08), 134.19 (133.82), 132.30, 130.99 (130.90), 62.65 (62.64), 53.81 (53.78), 48.18 (48.15), 43.39 (43.35), 42.45, 41.93 (41.89), 35.13 (35.11), 33.87, 30.83 (30.78), 29.81 (29.79), 26.98 (26.93), 25.00, 23.84 (23.82), 21.71 (21.69).

^{19}**F NMR** (565 MHz, CD_3OD, 21 °C) δ_F [ppm] –77.02.

LC-MS (ESI^+): *m/z* = 344 (70, $M+H^+$), 385 (9, $M+MeCN+H^+$).

$[\boldsymbol{\alpha}]_{\boldsymbol{D}}^{\mathbf{22}}$ = +9.7° (c = 0.39, MeOH).

IR ν [cm^{-1}] 3400-3300, 2950-2650, 1671, 1429, 1201, 1135, 838, 800, 722.

HR-MS (ESI+) exact mass calculated for $C_{20}H_{30}N_3O_2$ [$(M+H)^+$]: *m/z* = 344.2333; found: 344.2331.

Characterization data in agreement with material isolated from natural sources.[13]

3. Supplementary Results

3.1. Photocatalytic dehydrogenation of *N*-Boc-α-obscurine (S3)

Table S1. Screening of conditions for the photocatalytic dehydrogenation of **S3**.[a]

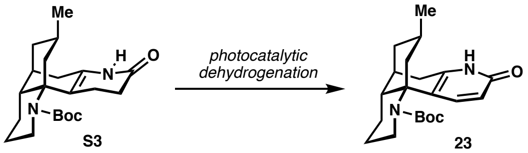

Entry	Redox mediator	Oxidant (eq)	Solvent	Conv. (%)	NMR-yield XX (%)
1	*Cu(MeCN)₄BF₄*[b,c]	$Na_2S_2O_8$ (5.5)	H_2O/acetone 1:1[d]	43[e]	<1
2	*Ag(NO)₃*[b,c]	$Na_2S_2O_8$ (5.5)	H_2O/acetone 1:1[d]	>99[e]	<1
3	*Riboflavin tetraacetate*	$(NH_4)_2S_2O_8$ (4.0)	H_2O/MeCN 1:1	>99[e]	11
4	Riboflavin tetraacetate	*$K_2S_2O_8$* (4.0)	H_2O/MeCN 1:1	>99	24
5	*Ir[dF(CF₃)ppy]₂(dtbpy)PF₆*	$(NH_4)_2S_2O_8$ (4.0)	H_2O/MeCN 1:1	>99[e]	51
6	Ir[dF(CF_3)ppy]$_2$(dtbpy)PF_6	$(NH_4)_2S_2O_8$ (*1.4*)	H_2O/MeCN 1:1	>99[e]	35
7	Ir[dF(CF_3)ppy]$_2$(dtbpy)PF_6	$(NH_4)_2S_2O_8$ (*2.7*)	H_2O/MeCN 1:1	>99[e]	40
8	Ir[dF(CF_3)ppy]$_2$(dtbpy)PF_6	$(NH_4)_2S_2O_8$ (*5.5*)	H_2O/MeCN 1:1	>99[e]	38
9	Ir[dF(CF_3)ppy]$_2$(dtbpy)PF_6	$(NH_4)_2S_2O_8$ (4.0)	H_2O/MeCN 1:1	>99	41
9	Ir[dF(CF_3)ppy]$_2$(dtbpy)PF_6	$(NH_4)_2S_2O_8$ (4.0)	H_2O/MeCN *2:1*	76	27
10	Ir[dF(CF_3)ppy]$_2$(dtbpy)PF_6	$(NH_4)_2S_2O_8$ (4.0)	H_2O/MeCN *1:2*	>99	23
11	Ir[dF(CF_3)ppy]$_2$(dtbpy)PF_6	*$Na_2S_2O_8$* (4.0)	H_2O/MeCN 1:1	>99	51
12	Ir[dF(CF_3)ppy]$_2$(dtbpy)PF_6	*$K_2S_2O_8$* (4.0)	H_2O/MeCN 1:1	>99	57
13	Ir[dF(CF_3)ppy]$_2$(dtbpy)PF_6 (*3.5 mol%*)	$K_2S_2O_8$ (4.0)	H_2O/MeCN 1:1	>99	14
14	Ir[dF(CF_3)ppy]$_2$(dtbpy)PF_6 (*14 mol%*)	$K_2S_2O_8$ (4.0)	H_2O/MeCN 1:1	>99	45
15	*none*	$(NH_4)_2S_2O_8$ (4.0)	H_2O/MeCN 1:1	<1	<1
16	Ir[dF(CF_3)ppy]$_2$(dtbpy)PF_6[f]	$(NH_4)_2S_2O_8$ (4.0)	H_2O/MeCN 1:1	6	<1
17	Ir[dF(CF_3)ppy]$_2$(dtbpy)PF_6	*Air (open vessel)*	H_2O/MeCN 1:1	>99[g]	<1

[a]General conditions: *N*-Boc-α-obscurine **S3** (10 mg, 27 µmol, 1.0 eq), oxidant (given eq), redox mediator (7 mol%) in the respective solvent (1.0 mL). Sparge with N_2 for 15 min. After irradiation with blue light (450 nm, 800 rpm, Merck photoreactor) at 21 °C for 20 min, the reaction mixture was extracted with CH_2Cl_2 (2 x 500 µL, phase separation through centrifugation), the extracts dried over $MgSO_4$ and evaporated under reduced pressure. Yields and conversion were measured by ^{1}H-NMR in $CDCl_3$ using ethylene carbonate (s, 4.52 ppm) as external standard. [b]40 °C for 1 h 45 min without light irradiation. [c]2.7 eq. of redox mediator. [d]500 µL solvent. [e]45 min reaction time. [f]In the dark. [g]Only decomposition of starting material was detected.

3.2. Biocatalytic oxidation methods

Table S2. Screening of biocatalytic conditions towards a chemoselective piperidine C–N bond oxidation of *N*-desmethyl-α-obscurine (**5**) and Boc-protected derivative (**S3**). Enzymes were selected based on their ability to oxidize C–heteroatom bonds in their substrate portfolio (references given).

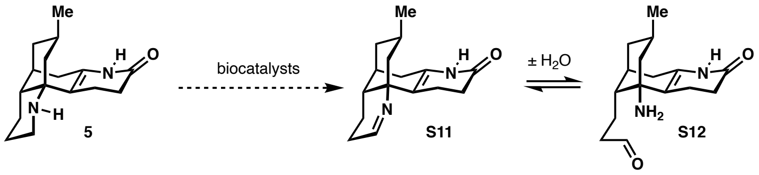

Entry	Substrate	Biocatalyst	Conditions[a]	Ref.	Result
1	**5**	Monoamine oxidase from *A. niger* (MAO-N); variants D5, D9 and D11	A	14	
2		Pyranose oxidase (PyrOx); 2 homologues	A	15	
3		Long-chain fatty alcohol oxidase (LCFAO)	A	15	
4		Hexose oxidase (HexOx)	A	15	
5		Galactose oxidase (GOx); variants M1, M3 and M3-5	A	16-18	
6		Gulose Oxidase (GulOx); 2 homologues	A	15	no reaction, recovery of starting material
7		Hydroxymethylfuran oxidase (HMFO)	A	19	
8		Berberine bridge enzyme from *E. californica* (*Ec*BBE)	B	20	
9		PQQ-dependent dehydrogenase from *D. mutans*	D	21	
10		Laccase / TEMPO	C	22	
11	*N*-Boc-**5** (**S3**)	Laccase / TEMPO	C	22	
12		Horseradish peroxidase (HRP)	E	23	

[a]Conditions: **A** Substrate (10 mM), KP_i-buffer (100 mM, pH 7.5, 0.5 mL) saturated with O_2, DMSO (10% v/v), lyophilized *E. coli* whole cells containing the het. expressed oxidase (20 mg/mL), catalase from bovine liver (1 mg/mL), 30 °C, 250 rpm (horizontal), 24 h; **B** Substrate (10 mM) , Tris-HCl buffer (50 mM, pH 9.0, 1.0 mL), $MgCl_2$ (10 mM), DMSO (10 %v/v), lyophilized purified BBE_*Ec* (3 mg/mL), catalase (1 mg/mL), 37 °C, 250 rpm (horizontal), 21 h; **C** Substrate (10 mM), citrate buffer (100 mM, pH 5.5, 1.0 mL), DMSO (10% v/v), TEMPO (2.5 mM), laccase *T. versicolor* (10 mg/mL), 37 °C, 250 rpm, 22 h; **D** Substrate (5 mM), Tris-HCl buffer (100 mM, pH 7.5, 1.0 mL) saturated with O_2, PQQ (0.1 mM), $K_3Fe(CN)_6$ (20 mM), DMSO (10 % v/v), lyophilized *E. coli* whole cells containing the het. expressed dehydrogenase from *D. mutans* (20 mg/mL), 30 °C, 250 rpm (horizontal), 24 h; **E** Substrate (10 mM), citrate buffer (100 mM, pH 5.5, 1.0 mL), glucose (50 mM), DMSO (10% v/v), HRP (30 U), glucose oxidase (30 U), 30–37 °C, 250 rpm, 25 h.

3.3. Synthesis of *trans*-8-oxo-7-phenyl-1-azabicyclo[4.2.0]octan-7-yl acetate (S13)

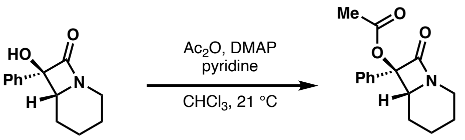

rac-**32** *rac*-**S13**

Racemic *trans*-α-hydroxy-β-lactam **32** (333 mg, 1.53 mmol, 1.0 equiv) was dissolved in anhydrous CH_2Cl_2 (2.5 mL) followed by addition of pyridine (612 µL, 7.65 mmol, 5.0 equiv) and *N*,*N*-dimethylaminopyridine (DMAP, 9.34 mg, 0.076 mmol, 0.05 equiv). The mixture was cooled to 0 °C and acetic anhydride (722 µL, 7.65 mmol, 5.0 equiv) was added dropwise over the course of 5 min. The reaction mix was allowed to warm to r.t. and was stirred over night, after which TLC (hexanes/EtOAc 1:2, $R_{f,prod}$ = 0.4, UV, $KMnO_4$) confirmed full consumption of **32**. Sat. aq. $NaHCO_3$ (5 mL) was added carefully, and the reaction was stirred at r.t. for another 30–60 min until gas evolution ceased. The mixture was extracted with CH_2Cl_2 (3 × 5 mL) and the combined organic extracts were washed with 1 M aq. HCl, sat. aq. $NaHCO_3$ and brine (5 mL each). The organic phases were dried over $MgSO_4$, and the oily residue was purified by silica gel chromatography (hexanes/EtOAc 1:1) to afford racemic ester **S13** as colorless oil (362 mg, 91% yield).

^{1}H NMR (300 MHz, $CDCl_3$) δ_H [ppm] 7.59–7.53 (m, 2H), 7.43–7.31 (m, 3H), 3.93 (ddd, *J* = 16.7, 12.4, 4.5 Hz, 2H), 2.88 (td, *J* = 12.7, 4.2 Hz, 1H), 2.09 (s, 3H), 1.84–1.69 (m, 2H), 1.67–1.56 (m, 1H), 1.51–1.11 (m, 2H), 0.91–0.73 (m, 1H).

^{13}C NMR (125 MHz, $CDCl_3$) δ_C [ppm] 169.6, 162.9, 133.5, 129.0, 128.5 (2C), 128.4 (2C), 92.7, 60.5, 39.0, 27.1, 24.2, 22.0, 21.5.

HR-MS (ESI^+) exact mass calculated for $C_{15}H_{18}NO_3$ [$(M+H)^+$]: *m/z* = 260.1281; found: 260.1274.

3.4. Synthesis of *trans*-8-oxo-7-phenyl-1-azabicyclo[4.2.0]octan-7-yl butyrate (S14)

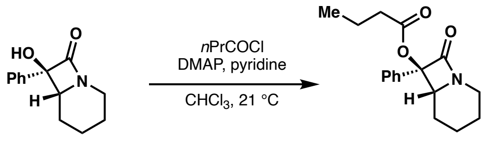

rac-**32** *rac*-**S14**

Racemic *trans*-*α*-hydroxy-*β*-lactam **32** (25.0 mg, 115 µmol, 1.0 equiv), DMAP (0.7 mg, 5.7 µmol, 5.0 mol%) and pyridine (30.0 µL, 575 µmol, 5.0 equiv) were dissolved in anhydrous CH_2Cl_2 (120 µL) and cooled to 0 °C. Butyrylchloride (60.0 µL, 575 µmol, 5.0 equiv) was added dropwise over the course of 5 min and the reaction mix was allowed to warm to r.t. After stirring overnight, TLC (hexanes/EtOAc 1:2, $R_{f,prod}$ = 0.4, UV, $KMnO_4$) confirmed full consumption of **32**. Sat. aq. $NaHCO_3$ (500 µL) was added carefully, and the reaction was stirred at r.t. for another 15 min until gas evolution ceases. The mixture was extracted with CH_2Cl_2 (3 × 750 µL) and the combined organic extracts were washed with 1 M aq. HCl, sat. aq. $NaHCO_3$ and brine (750 µL each) (phases separated by centrifugation). The organic phases were dried over $MgSO_4$, and the yellow oily residue was purified by silica gel chromatography (hexanes/EtOAc 1:1) to afford racemic ester **S14** as colorless oil (30.1 mg, 91% yield).

^{1}H NMR (300 MHz, $CDCl_3$) δ_H [ppm] 7.60–7.53 (m, 2H), 7.40–7.31 (m, 3H), 3.93 (td, *J* = 14.5, 13.0, 4.5 Hz, 2H), 2.88 (td, *J* = 12.7, 4.2 Hz, 1H), 2.33 (t, *J* = 7.4 Hz, 2H), 1.84–1.68 (m, 2H), 1.63 (q, *J* = 7.4 Hz, 3H), 1.49 – 1.16 (m, 2H), 0.99–0.79 (m) superimposed on 0.90 (t, *J* = 7.4 Hz, 5H total).

^{13}C NMR (125 MHz, $CDCl_3$) δ_C [ppm] 172.4, 163.0, 133.7, 129.0, 128.5 (2C), 128.4 (2C), 92.6, 60.6, 39.0, 36.4, 27.1, 24.2, 22.0, 18.3, 13.7.

HR-MS (ESI^+) exact mass calculated for $C_{17}H_{22}NO_3$ [$(M+H)^+$]: *m/z* = 288.1594; found: 288.1588.

3.5. Enzymatic kinetic resolution of the *β*-lactam coupling partner

All lipase- and esterase preparations used in kinetic resolution experiments were obtained from Sigma Aldrich and used as received:

- Esterase from porcine liver (PLE, 20 U/mg and 69.3 U/mg, respectively)
- Lipase A from *Candida antarctica* immobilized on immobead 150 (CalA, 1.59 U/mg, recombinant from *A. oryzae*)
- Lipase acrylic resin from *Candida antarctica* (CalB, ≥ 5 U/mg, recombinant from *A. niger*)
- Amano lipase PS-IM immobilized on diatomaceous earth (no activity given)
- Lipase from Candida rugosa (CRL Type VII, 1.117 U/mg)
- Pankreatin from porcine pancreas (4 x USP specifications, no activity given).

Table S3. Screening of hydrolases for the hydrolytic kinetic resolution of esters **S13**–**S14**.[a]

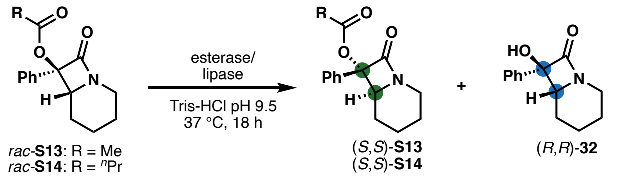

Entry	Enzyme	R	*e.e.* of **S13/S14** (%)	*e.e.* of **32** (%)	Conv. (%)	Selectivity E[24]
1	Lipase A from *Candida antarctica* (CalA)	Me	1 (*S,S*)	3 (*R,R*)	22	1
2	Lipase A from *Candida antarctica* (CalA)	*n*-Pr	2 (*S,S*)	2 (*R,R*)	9	1
3	Esterase form porcine liver (PLE)	Me	94 (*S,S*)	8 (*R,R*)	90	3
4	Esterase form porcine liver (PLE)	*n*-Pr	1	1	93	1
5	Amano-Lipase PS	Me	<1	2	13	n.c.
6	Amano-Lipase PS	*n*-Pr	1	1	17	1
7	Lipase from *Candida rugosa* (CRL)	Me	<1	<1	13	n.c.
8	Pancreatin from hog pancreas	Me	<1	1	14	n.c.

[a]Conditions: Enzyme preparation (3.0 mg/mL) Tris-HCl buffer (100 mM, pH 9.5), substrate (50 mM), 37 °C, 24 h.

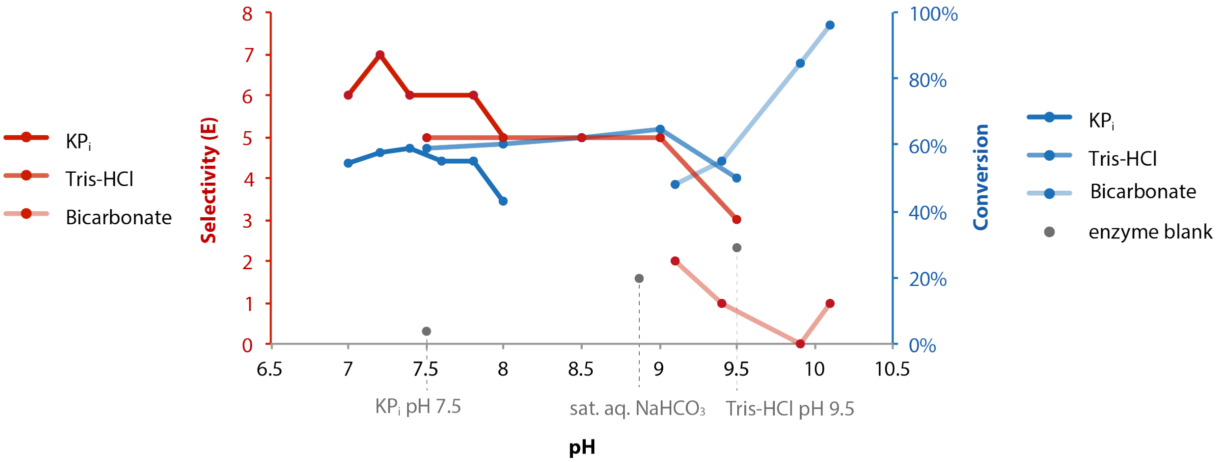

Figure S1. pH-Dependence of the PLE-catalyzed hydrolytic kinetic resolution of racemic acetate **S13**. Conditions: PLE (100 U), substrate **S13** (50 mM) in the respective buffer system with 10% v/v ^{t}BuOH. PLE was omitted in enzyme blanks. Pronounced background hydrolysis (grey points) leading to erosion of selectivity was observed with increasing pH. Highest selectivities were observed in a KP$_i$-buffer system at pH 7.3 (E = 7, conv. = 60%).

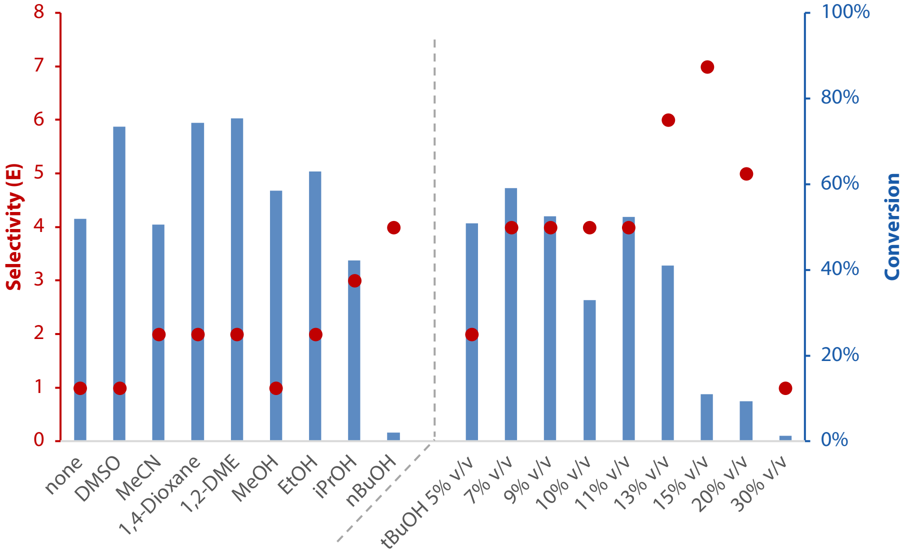

Figure S2. Effect of organic co-solvent on the PLE-catalyzed hydrolytic kinetic resolution of acetate **S13**. (●): Selectivity; (■): Conversion. Conditions: PLE (100 U), substrate **S13** (50 mM) in KP$_i$-buffer (50 mM, pH 7.3) with 10% v/v of the respective co-solvent (unless otherwise noted). Highest selectivities were observed with 15% v/v ^{t}BuOH (E = 7, conv. = 11%).

Table S4. Screening of hydrolases for the acylative kinetic resolution of racemic *trans*-α-hydroxy-β-lactam **32**.[a]

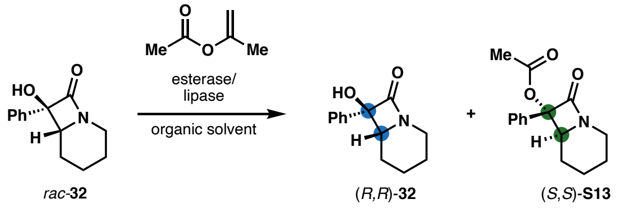

Entry	Enzyme	Solvent	*e.e.* of **32** (%)	*e.e.* of **S13** (%)	Conv. (%)	Selectivity E
1	Lipase B from *Candida antarctica* (CalB)	MTBE	<1	<1	<1	n.c.[b]
2	Amano-Lipase PS	MTBE	1	3 (*R*,*R*)	1	1
3	Lipase A from *Candida antarctica* (CalA)	MTBE	3 (*R*,*R*)	40 (*S*,*S*)	7	2
4	Lipase A from *Candida antarctica* (CalA)	1,4-dioxane	1	52 (*S*,*S*)	8	3
5	Lipase A from *Candida antarctica* (CalA)	*n*-hexane	2	4 (*S*,*S*)	19	1
6	Lipase A from *Candida antarctica* (CalA)	*i*-octane	1	16 (*S*,*S*)	22	1
7	Lipase A from *Candida antarctica* (CalA)	toluene	2	37 (*S*,*S*)	8	2

[a]Conditions: α-Hydroxy-β-lactam **32** (5.0 mg, 23 µmol), enzyme preparation (2.0 mg/mL) and isopropenyl acetate (12.5 µL, 115 µmol, 5.0 eq) in anhydrous solvent (1.0 mL); incubation for 24 h (250 rpm, 37 °C). Extraction with EtOAc (2 × 1 mL), dry over $MgSO_4$, evaporate solvent under red. pressure, re-dissolve in *n*-hexane/*i*PrOH 9:1 for HPLC analytics. [b]n.c. = not calculated.

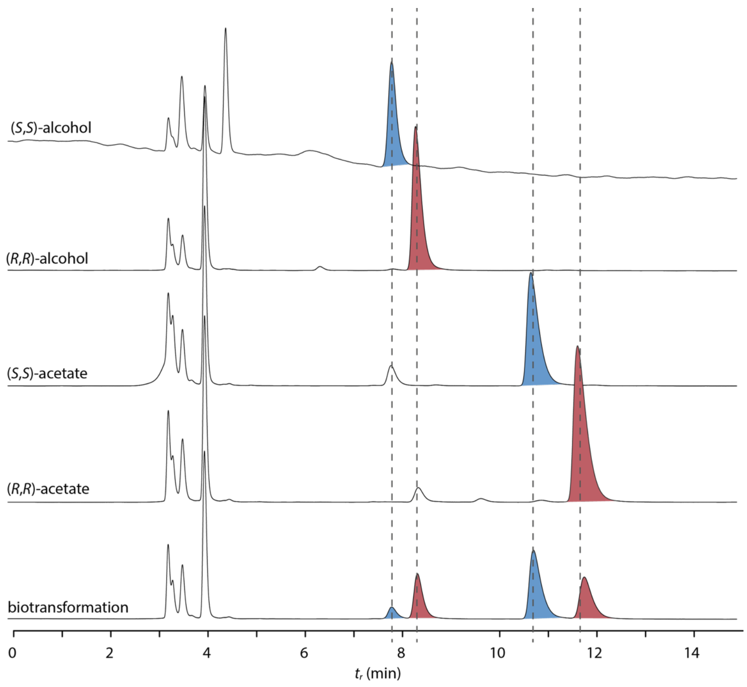

Figure S3. HPLC-analytics for ester **S13** and alcohol **32**. Separation of all product- and substrate isomers was performed on a Shimadzu VP series HPLC system equipped with a DAD using a Daicel Chiralcel IB column with an isocratic mixture of hexanes/*i*PrOH (9:1) as eluent. (*S*,*S*)-alcohol **32**: 7.9 min, (*R*,*R*)-alcohol **32**: 8.5 min, (*S*,*S*)-ester **S13**: 11.0 min, (*R*,*R*)-ester **S13**: 12.0 min.

4. Comparison of NMR spectroscopic data for synthetic and isolated alkaloids

Table S5. Comparison of ^{1}H- and ^{13}C NMR spectroscopic data for casuarinine H (**2**)[a]

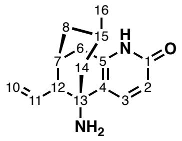

	δ_H (ppm)		δ_C (ppm)	
Position	Lit.[b]	Found[c]	Lit.[b]	Found[c]
1	–	–	164.9	165.0
2	6.44, d (9.2)	6.43, d (9.3)	118.9	119.2
3	7.76, d (9.2)	7.76, d (9.4)	140.3	140.4
4	–	–	120.5	120.5
5	–	–	143.7	143.9
6	3.06, dd (18.8, 7.1)	3.06, dd (18.9, 7.1)	29.9	30.0
	2.51, br d (18.8)	2.51, d (18.8)		
7	2.30, m	2.29, m	34.2	34.3
8	1.75, br d (12.8)	1.74, d (13.3)	42.5	42.6
	1.29, ddd (12.8, 12.7, 3.7)	1.29, ddd (12.8, 12.8, 4.1)		
10	5.23, dd (16.8, 1.8)	5.23, dd (17.0, 2.1)	117.2	117.3
	5.14, dd (10.4, 2.0)	5.13, dd (10.2, 2.1)		
11	5.62, ddd (16.8, 10.4, 9.6)	5.62, ddd (16.9, 9.8, 9.8)	137.3	137.4
12	2.14, dd (9.6, 2.9)	2.15, dd (9.0, 2.5)	49.9	50.0
13	–	–	54.8	54.8
14	1.64, dd (12.0, 3.9)	1.65, dd (11.7, 3.6)	52.2	52.4
	1.09, dd (12.0, 12.0)	1.10, dd (12.1, 12.1)		
15	1.40, m	1.41, m	26.3	26.5
16	0.85, d (6.4)	0.85, d (6.4)	21.7	21.9
N–H	13.32, br s	13.25, br s	–	–

[a]Coupling constants in parentheses (*J*) are given in Hz. [b]Isolated natural product measured in $CDCl_3$ at 400 MHz (^{1}H) and 100 MHz (^{13}C).[7]
[c]Synthetic sample measured in $CDCl_3$ at 500 MHz (^{1}H) and 151 MHz (^{13}C).

Table S6. Comparison of ^{1}H- and ^{13}C NMR spectroscopic data for 8,15-dihydrohuperzine A (**3**)[a]

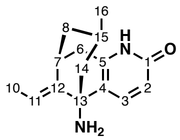

	δ_H (ppm)			δ_C (ppm)		
Position	Lit.[b]	Lit.[c]	Found[d]	Lit.[b]	Lit.[c]	Found[d]
1	–	–	–	165.0	164.2	164.9
2	6.40, d (9.4)	6.41, d (9.5)	6.41, d (9.4)	116.8	117.2	117.1
3	7.82, d (9.4)	7.82, d (9.5)	7.82, d (9.4)	139.5	139.4	139.6
4	–	–	–	122.2	122.1	122.3
5	–	–	–	144.5	143.6	144.4
6	2.71, d (18.3)	2.62, d (17.8)	2.69, d (18.1)	34.4	34.5	34.6
	3.02, dd (18.3, 7.1)	3.00, dd (17.8, 7.7)	3.01, dd (18.2, 7.3)			
7	3.32, br s	3.33, m	3.33, m	30.6	30.7	30.7
8	1.27–1.13, m	1.20, dt (12.6, 4.0)	1.19, dt (12.8, 4.4)	42.7	42.7	42.8
10	1.66, d (6.7)	1.67, d (6.7)	1.67, d (6.7)	12.3	12.2	12.4
11	Not reported	5.51, q (6.7)	5.50, q (6.7)	111.3	111.2	111.4
12	–	–	–	144.5	144.5	144.6
13	–	–	–	55.1	54.8	55.1
14	Not reported	1.05, t (12.0)	1.05, t (11.9)	51.2	51.2	51.3
	1.80–1.57, m	1.78, br t (10.2)	1.72, br d, (12.2)			
15	1.80–1.57, m	1.60, m	1.60 (m)	26.5	26.5	26.6
16	0.81, d (6.3)	0.83, d (6.5)	0.82, d (6.5)	21.4	21.3	21.5
13′ (NH_2)	Not reported	Not reported	1.64-1.53, br s	–	–	–
1′ (NH)	11.4–10.9, br s	Not reported	12.83, br s	–	–	–

[a]Coupling constants in parentheses (*J*) are given in Hz. [b]Synthetic sample measured in $CDCl_3$ at 300 MHz.[25] [c]Isolated natural product measured in $CDCl_3$ at 600 MHz (^{1}H) and 150 MHz (^{13}C).[10] [d]Synthetic sample measured in $CDCl_3$ at 600 MHz (^{1}H) and 151 MHz (^{13}C).

Table S7. Comparison of ^{1}H- and ^{13}C NMR spectroscopic data for lycoplatyrine B (**4**)[a]

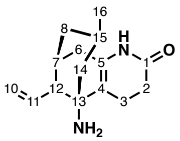

	δ_H (ppm)		δ_C (ppm)	
Position	Lit.[b]	Found[c]	Lit.[b]	Found[c]
1	–	–	171.5	171.2
2	2.43, m	2.48–2.40, m	31.1	31.2
3	2.26, m	2.27, m	19.8	19.9
	2.42, m	2.48–2.40, m		
4	–	–	113.9	114.2
5	–	–	129.8	129.6
6	1.72, d (18.0)	1.71, d (18.1)	30.1	30.2
	2.43, m	2.48–2.40, m		
7	2.11, m	2.13, m	34.4	34.6
8	1.21, br t (14.0[d]) [ax]	1.25–1.20, m	42.8	43.0
	1.68, m [eq]	1.68–1.64, m		
10	5.11, dd (10.0, 2.0)	5.13, dd (10.2, 2.2)	118.3	118.3
	5.19, dd (17.0, 2.0)	5.21, dd (17.1, 2.1)		
11	5.79, dt (17.0, 10.0)	5.81, dt (17.1, 9.8)	138.3	138.4
12	2.03, dd (10.0, 2.8)	2.04, dd (9.4, 3.0)	54.8	55.0
13	–	–	53.1	53.0
14	0.89, m [ax]	0.89, m	46.9	47.1
	1.68, m [eq]	1.68–1.64, m		
15	1.66, m	1.68–1.64, m	26.9	27.0
16	0.87, d (6.0)	0.89, d (6.1)	21.9	22.0
N–H	7.66, br s	7.17, br s	–	–

[a]Coupling constants in parentheses (*J*) are given in Hz. [b]Isolated natural product measured in $CDCl_3$ at 400 MHz (^{1}H) and 100 MHz (^{13}C).[8] [c]Synthetic sample measured in $CDCl_3$ at 600 MHz (^{1}H) and 151 MHz (^{13}C). [d]Coupling constant determined from homonuclear decoupling experiments.

Table S8. Comparison of ^{1}H NMR spectroscopic data for lycoplatyrine A (**8**).[a]

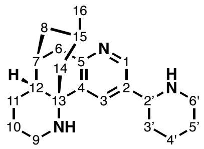

Position	Lit. (major isomer)[b]	Found (2′*S* epimer)[c]	Lit. (minor isomer)[b]	Found (2′*R* epimer)[c]
1	8.33, d (2.0)	8.32, d (2.1)	8.36, d (2.0)	8.37, d (2.0)
3	7.77, d (2.0)	7.74, d (2.0)	7.76, d (2.0)	7.72, d (2.0)
6	2.70, d (18.6)	2.69, d (18.6)	2.70, d (18.6)	2.69, d (18.6)
	3.14, dd (18.6, 7.2)	3.13, dd (18.6, 7.1)	3.14, dd (18.6, 7.2)	3.13, dd (18.1, 7.1)
7	2.09, m	2.08, m	2.09, m	2.08, m
8	1.34, td (12.4, 3.8) [ax]	1.33, td (12.3, 3.8)	1.34, td (12.4, 3.8) [ax]	1.33, td (12.4, 3.8)
	1.77, m [eq]	1.82–1.64, m	1.77, m [eq]	1.84–1.64, m
9	2.43, m [ax]	2.42, m	2.43, m [ax]	2.41, m
	2.79, m [eq]	2.79, m	2.79, m [eq]	2.83–2.74, m
10	1.56, m	1.56–1.47, m	1.56, m	1.61–1.47, m
	1.56, m	1.56–1.47, m	1.56, m	1.61–1.47, m
11	1.19, m [ax]	1.27–1.12, m	1.19, m [ax]	1.28–1.12, m
	1.53, m [eq]	1.56–1.47, m	1.53, m [eq]	1.61–1.47, m
12	1.61, m	1.60, dt (12.4, 2.9)	1.61, m	1.61–1.47, m
14	1.19, m [ax]	1.27–1.12, m	1.19, m [ax]	1.28–1.12, m
	1.46, d (10.2) [eq]	1.44, d (10.3)	1.46, d (10.2) [eq]	1.44, d (10.4)
15	1.22, m	1.27–1.12, m	1.22, m	1.28–1.12, m
16	0.77, d (5.9)	0.76, d (6.0)	0.78, d (5.9)	0.77, d (6.1)
2′	3.63, d (9.2)	3.61, d (10.5)	3.63, d (9.2)	3.62, d (10.0)
3′	1.53, m	1.56–1.47, m	1.53, m	1.61–1.47, m
	1.81, m	1.82–1.64, m	1.81, m	1.84–1.64, m
4′	1.53, m	1.56–1.47, m	1.53, m	1.61–1.47, m
	1.91, m	1.90, m	1.91, m	1.90, m
5′	1.55, m	1.56–1.47, m	1.55, m	1.61–1.47, m
	1.67, m	1.82–1.64, m	1.67, m	1.84–1.64, m
6′	2.80, m	2.79, m	2.80, m	2.83–2.74, m
	3.21, dd (11.6, 1.6)	3.20, d (11.6)	3.21, dd (11.6, 1.6)	3.20, d (11.5)

[a]Chemical shifts (δ_H) are given in ppm, coupling constants in parentheses (*J*) are given in Hz. [b]1.3:1 Mixture of epimers isolated from natural sources measured in $CDCl_3$ at 600 MHz.[8] [c]Synthetic samples of individual epimers (>95:5 *d.r.*) measured in $CDCl_3$ at 500 MHz.

Table S9. Comparison of ^{13}C NMR spectroscopic data for lycoplatyrine A (**8**).[a]

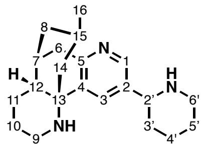

Position	Lit. (major isomer)[b]	Found (2′*S* epimer)[c]	Lit. (minor isomer)[b]	Found (2′*R* epimer)[c]
1	145.54[d]	145.89	145.72[d]	145.67
2	138.54[d]	138.76	138.59[d]	138.83
3	131.10[d]	131.15	131.33[d]	131.38
4	135.68[d]	135.93	135.56[d]	135.81
5	157.40[d]	157.73	157.55[d]	157.55
6	35.01[d]	35.15	35.05[d]	35.28
7	33.71[d]	33.88	33.74[d]	33.92
8	43.80[d]	44.00	43.81[d]	43.99
9	41.34[d]	41.50	41.44[d]	41.61
10	27.72[d]	27.92	27.67[d]	27.99
11	26.25	26.34	26.15	26.35
12	44.49[d]	44.69	44.55[d]	44.78
13	56.29[d]	56.36	56.33[d]	56.30
14	51.25[d]	51.50	51.37[d]	51.64
15	25.80[d]	25.93	25.81[d]	25.96
16	22.05[d]	22.22	22.07[d]	22.24
2′	59.82[d]	60.01	59.94[d]	60.14
3′	34.92	35.27	34.92	35.22
4′	25.27[d]	25.46	25.29[d]	25.49
5′	25.71[d]	25.93	25.74[d]	25.96
6′	47.73	47.92	47.73	47.92

[a]Chemical shifts (δ_C) are given in ppm. [b]1.3:1 Mixture of epimers isolated from natural sources measured in $CDCl_3$ at 150 MHz.[8] [c]Synthetic sample of individual epimers (>95:5 *d.r.*) measured in $CDCl_3$ at 125 MHz. [d]Signals annotated as interchangeable among epimers.

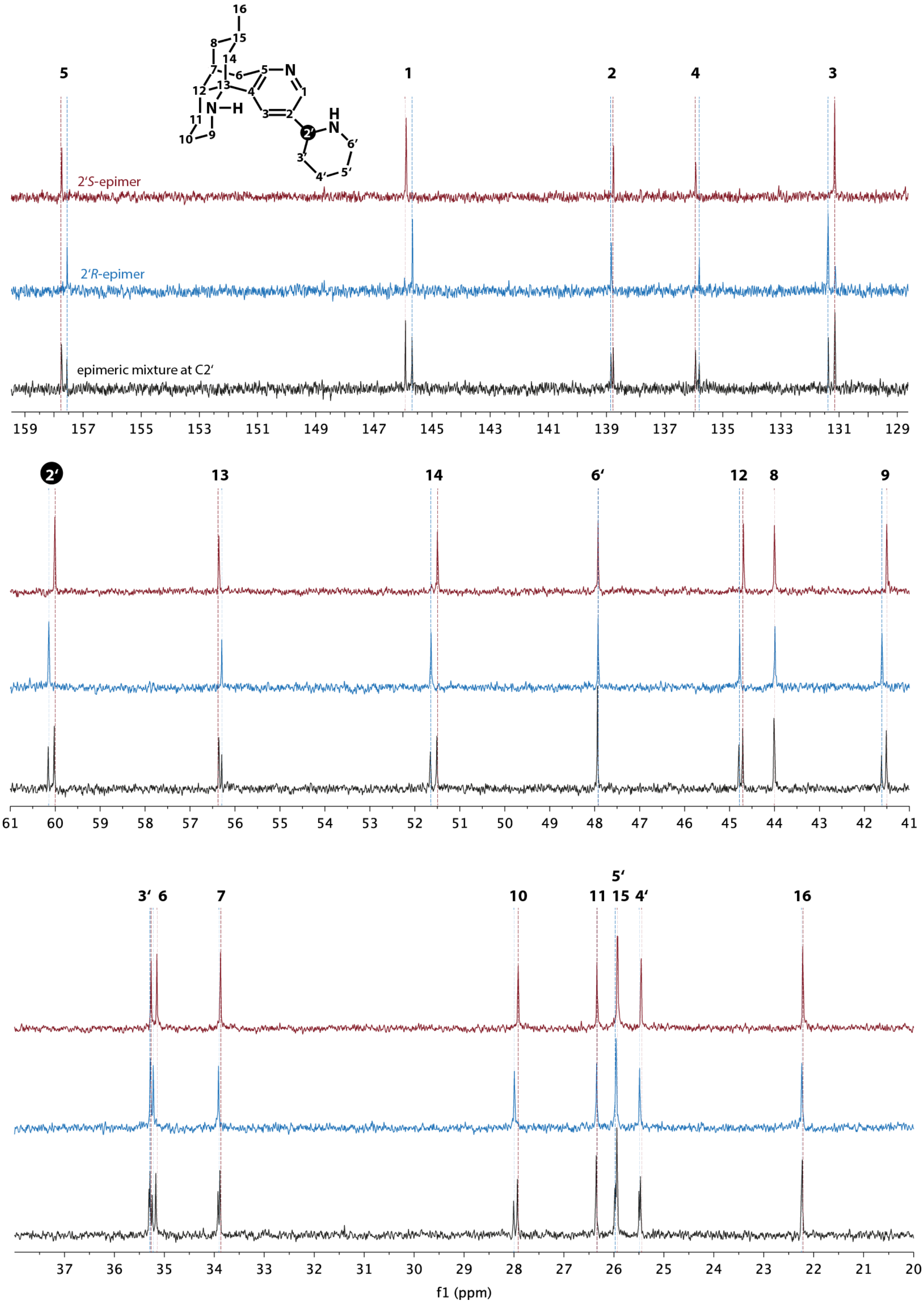

Figure S4. Overlay of ^{13}C-NMR spectra (125 MHz, $CDCl_3$) of pure C2′ epimers (>95:5 *d.r.*) and an epimeric mixture of **8** obtained from coupling *N*-Boc-2-bromolycodine (**31**) with racemic lactam **32**.

Table S10. Comparison of ^{1}H- and ^{13}C NMR spectroscopic data for lycopladine F (epimeric mixture at C2′) (**9**)[a]

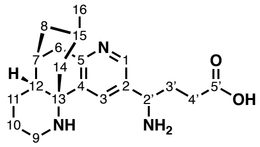

	δ_H (ppm)		δ_C (ppm)	
Position	Lit.[b]	Found[c]	Lit.[b]	Found[c]
1	8.59, s; 8.61, s	8.61, d (2.0); 8.58, d (2.0)	149.4	175.24; 175.19
2	–	–	132.3	132.30
3	8.24, s; 8.16, s	8.25, d (2.1); 8.17, d (2.1)	133.8	133.82; 134.19
4	–	–	131.0	130.99; 130.90
5	–	–	160.8	160.81; 160.76
6	3.28, m	3.29–3.19, m	35.2	35.13; 35.11
	2.82, d (19.2); 2.83, d (19.2)	2.83, d (19.4); 2.84, d (19.3)		
7	2.35, m	2.43–2.29, m	33.9	33.87
8	1.87, m	1.94–1.84, m	43.4	43.39; 43.35
	1.47, ddd (13.2, 12.6, 3.6)	1.47, ddd (13.0, 12.9, 3.4)		
9	3.28, m	3.29–3.19, m	41.9	41.93; 41.89
	2.94, ddd (13.2, 12.6, 3.6)	2.99–2.90, m		
10	1.88, m	1.94–1.84, m	23.8	23.84; 23.82
11	1.73, br d (13.2)	1.74, br d (13.5)	25.0	25.00
	1.34, m	1.38–1.22, m		
12	2.09, br d (12.6)	2.09, br d (12.4)	42.4	42.45
13	–	–	62.7	62.65; 62.64
14	1.89, m	1.94–1.84, m	48.2	48.18; 48.15
	1.63, dd (12.0, 12.0)	1.62, dd (12.0, 12.0)		
15	1.23, m	1.38–1.22, m	27.0	26.98; 26.93
16	0.87, d (6.6); 0.88, d (6.6)	0.88, d (6.6); 0.89, d (6.6)	21.7	21.71 (21.69)
2′	4.50, m; 4.51, m	4.50, m	53.8	53.81; 53.78
3′	2.38, m	2.43–2.29, m	30.4	30.83; 30.78
4′	2.42, m	2.43–2.29, m	29.8	29.81; 29.79
	2.36, m	2.43–2.29, m		
5′	–	–	175.8	175.24; 175.19

[a]Coupling constants in parentheses (*J*) are given in Hz. [b]1:3.5 mixture of 2′*R* and 2′*S* epimers isolated from natural sources measured in CD_3OD at 600 MHz. In cases where the diastereomers resolve, the signal for the minor diastereomer is listed second.[26] [c]Synthetic sample of epimeric mixture (1:1 *d.r.*) measured in CD_3OD at 600 MHz (^{1}H) and 151 MHz (^{13}C). In cases where the diastereomers resolve, the signal for the second diastereomer is also given.

5. References

1. Fischer, D. F.; Sarpong, R., Total synthesis of (+)-complanadine A using an iridium-catalyzed pyridine C–H functionalization. *J. Am. Chem. Soc.* **2010**, *132*, 5926–5927.
2. Katsuhara, J., Absolute configuration of pulegone oxide and piperitenone dioxide. *J. Org. Chem.* **1967**, *32*, 797–799.
3. Elgendy, E. M.; Khayyat, S. A., Oxidation studies on some natural monoterpenes: Citral, pulegone, and camphene. *Russian J. Org. Chem.* **2008**, *44*, 814–822.
4. Caine, D.; Procter, K.; Cassell, R. A., A facile synthesis of (–)-R-5-methyl-2-cyclohexen-1-one and related 2-substituted enones from (+)-pulegone. *J. Org. Chem.* **1984**, *49*, 2647–2648.
5. Liu, K. M.; Chau, C. M.; Sha, C. K., Intermolecular radical addition reactions of alpha-iodo cycloalkenones and a synthetic study of the formal synthesis of enantiopure fawcettimine. *Chem. Commun.* **2008**, 1, 91–93.
6. Schuster, E.; Jas, G.; Schumann, D., An improved synthesis of 2,3,4,6,7,8-hexahydroquinolines. *Org. Prep. Proced. Int.* **1992**, *24*, 670–672.
7. Tang, Y.; Fu, Y.; Xiong, J.; Li, M.; Ma, G. L.; Yang, G. X.; Wei, B. G.; Zhao, Y.; Zhang, H. Y.; Hu, J. F., Casuarinines A–J, lycodine-type alkaloids from *Lycopodiastrum casuarinoides*. *J. Nat. Prod.* **2013**, *76*, 1475–1484.
8. Yeap, J. S. Y.; Lim, K. H.; Yong, K. T.; Lim, S. H.; Kam, T. S.; Low, Y. Y., *Lycopodium* alkaloids: Lycoplatyrine A, an unusual lycodine-piperidine adduct from *Lycopodium platyrhizoma* and the absolute configurations of lycoplanine D and lycogladine H. *J. Nat. Prod.* **2019**, *82*, 324–329.
9. Kozikowski, A. P.; Miller, C. P.; Yamada, F.; Pang, Y. P.; Miller, J. H.; McKinney, M.; Ball, R. G., Delineating the pharmacophoric elements of huperzine A — The importance of the saturated 3-carbon bridge to its AChE inhibitory activity. *J. Med. Chem.* **1991**, *34*, 3399–3402.
10. Thorroad, S.; Worawittayanont, P.; Khunnawutmanotham, N.; Chimnoi, N.; Jumruksa, A.; Ruchirawat, S.; Thasana, N., Three new *Lycopodium* alkaloids from *Huperzia carinata* and *Huperzia squarrosa*. *Tetrahedron* **2014**, *70*, 8017–8022.
11. Zhao, L.; Tsukano, C.; Kwon, E.; Shirakawa, H.; Kaneko, S.; Takemoto, Y.; Hirama, M., Competent Route to Unsymmetric Dimer Architectures: Total Syntheses of (–)-Lycodine and (–)-Complanadines A and B, and Evaluation of Their Neurite Outgrowth Activities. *Chem. Eur. J.* **2017**, *23*, 802–812.
12. Roque, J. B.; Kuroda, Y.; Jurczyk, J.; Xu, L. P.; Ham, J. S.; Gottemann, L. T.; Roberts, C. A.; Adpressa, D.; Sauri, J.; Joyce, L. A.; Musaev, D. G.; Yeung, C. S.; Sarpong, R., C–C cleavage approach to C–H functionalization of saturated aza-cycles. *ACS Catal.* **2020**, *10*, 2929–2941.
13. Ishiuchi, K.; Kubota, T.; Hayashi, S.; Shibata, T.; Kobayashi, J., Lycopladines F and G, new C16N2-type alkaloids with an additional C4N unit from *Lycopodium complanatum*. *Tetrahedron Lett.* **2009**, *50*, 4221–4224.
14. Ghislieri, D.; Green, A. P.; Pontini, M.; Willies, S. C.; Rowles, I.; Frank, A.; Grogan, G.; Turner, N. J., Engineering an enantioselective amine oxidase for the synthesis of pharmaceutical building blocks and alkaloid natural products. *J. Am. Chem. Soc.* **2013**, *135*, 10863–10869.
15. Pickl, M.; Fuchs, M.; Glueck, S. M.; Faber, K., The substrate tolerance of alcohol oxidases. *Appl. Microbiol. Biotechnol.* **2015**, *99*, 6617–6642.
16. Sun, L. H.; Bulter, T.; Alcalde, M.; Petrounia, I. P.; Arnold, F. H., Modification of galactose oxidase to introduce glucose 6-oxidase activity. *ChemBioChem* **2002**, *3*, 781–783.
17. Vilim, J.; Knaus, T.; Mutti, F. G., Catalytic promiscuity of galactose oxidase: A mild synthesis of nitriles from alcohols, air, and ammonia. *Angew. Chem. Int. Ed.* **2018**, *57*, 14240–14244.
18. Escalettes, F.; Turner, N. J., Directed evolution of galactose oxidase: generation of enantioselective secondary alcohol oxidases. *ChemBioChem* **2008**, *9*, 857–860.
19. Gandomkar, S.; Jost, E.; Loidolt, D.; Swoboda, A.; Pickl, M.; Elaily, W.; Daniel, B.; Fraaije, M. W.; Macheroux, P.; Kroutil, W., Biocatalytic enantioselective oxidation of *sec*-allylic alcohols with flavin-dependent oxidases. *Adv. Synth. Catal.* **2019**, *361*, 5264–5271.
20. Schrittwieser, J. H.; Groenendaal, B.; Resch, V.; Ghislieri, D.; Wallner, S.; Fischereder, E. M.; Fuchs, E.; Grischek, B.; Sattler, J. H.; Macheroux, P.; Turner, N. J.; Kroutil, W., Deracemization by simultaneous bio-oxidative kinetic resolution and stereoinversion. *Angew. Chem. Int. Ed.* **2014**, *53*, 3731–3734.
21. Takeda, K.; Umezawa, K.; Varnai, A.; Eijsink, V. G. H.; Igarashi, K.; Yoshida, M.; Nakamura, N., Fungal PQQ-dependent dehydrogenases and their potential in biocatalysis. *Curr. Opin. Chem. Biol.* **2019**, *49*, 113–121.
22. Diaz-Rodriguez, A.; Martinez-Montero, L.; Lavandera, I.; Gotor, V.; Gotor-Fernandez, V., Laccase/2,2,6,6-tetramethylpiperidinoxyl radical (TEMPO): An efficient catalytic system for selective oxidations of primary hydroxy and amino groups in aqueous and biphasic media. *Adv. Synth. Catal.* **2014**, *356*, 2321–2329.
23. Thiel, D.; Doknic, D.; Deska, J., Enzymatic aerobic ring rearrangement of optically active furylcarbinols. *Nature Comm.* **2014**, *5*, 5278.
24. The selectivity for the kinetic resolution process was calculated from the *e.e.* values of remaining starting material and formed product using the "Enantio" online tool: http://biocatalysis.uni-graz.at/biocatalysis-tools/enantio. (accessed on June 20, 2020)
25. Synthetically prepared sample of **3**: Kozikowski, A. P.; Miller, C. P.; Yamada, F.; Pang, Y. P.; Miller, J. H.; McKinney, M.; Ball, R. G., Delineating the Pharmacophoric Elements of Huperzine A: Importance of the Unsaturated Three-Carbon Bridge to its AChE Inhibitory Activity. *J. Med. Chem.* **1991**, *34*, 3399–3402.

26. Sample of **3** isolated from natural sources: Ishiuchi, K.; Kubota, T.; Hayashi, S.; Shibata, T.; Kobayashi, J., Lycopladines F and G, new $C_{16}N_2$-type alkaloids with an additional C_4N unit from *Lycopodium complanatum. Tetrahedron Lett.* **2009**, *50*, 4221–4224.

6. NMR Spectra

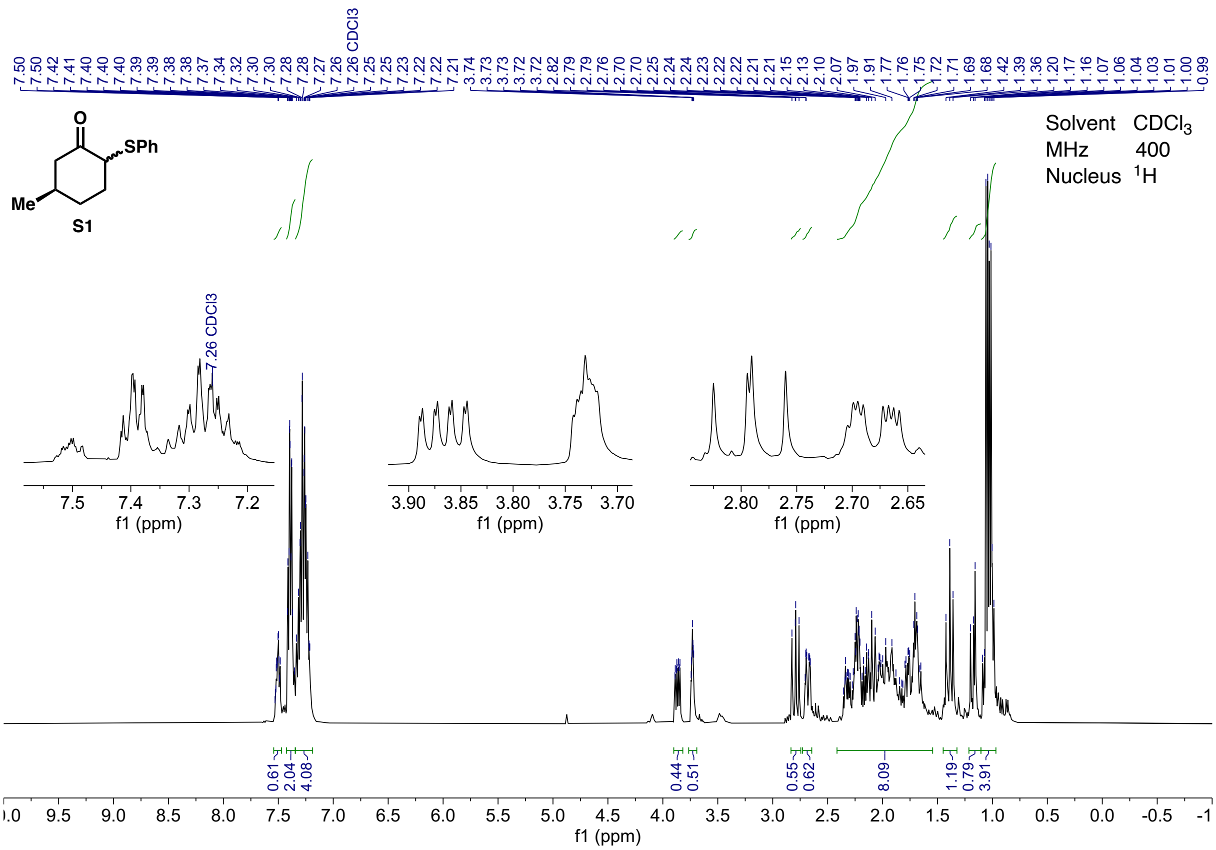
SPh
Me
S1
Solvent CDCl3
MHz 400
Nucleus 1H
7.50 7.50 7.42 7.41 7.40 7.40 7.40 7.39 7.39 7.38 7.37 7.34 7.32 7.30 7.30 7.28 7.28 7.27 7.26 CDCl3 7.25 7.25 7.23 7.22 7.22 7.21 3.74 3.73 3.72 3.72 2.82 2.79 2.76 2.70 2.70 2.25 2.24 2.24 2.23 2.22 2.22 2.22 2.21 2.21 2.15 2.13 2.10 2.07 1.97 1.91 1.77 1.76 1.75 1.72 1.71 1.69 1.68 1.42 1.39 1.36 1.20 1.17 1.16 1.07 1.06 1.04 1.03 1.01 1.00 0.99
0.61 2.04 4.08 0.44 0.51 0.55 0.62 8.09 1.19 0.79 3.91
f1 (ppm)

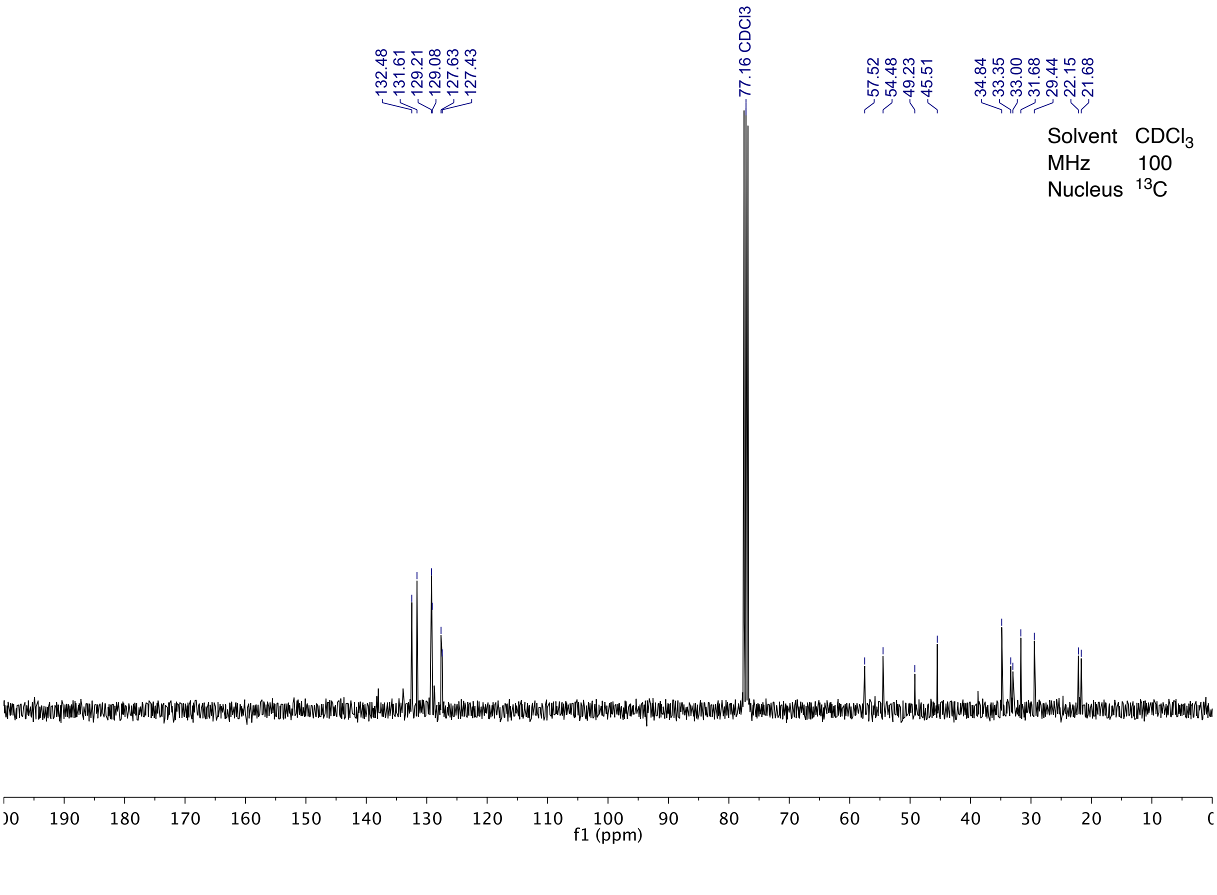
132.48 131.61 129.21 129.08 127.63 127.43
77.16 CDCl3
57.52 54.48 49.23 45.51
34.84 33.35 33.00 31.68 29.44 22.15 21.68
Solvent CDCl3
MHz 100
Nucleus 13C
f1 (ppm)

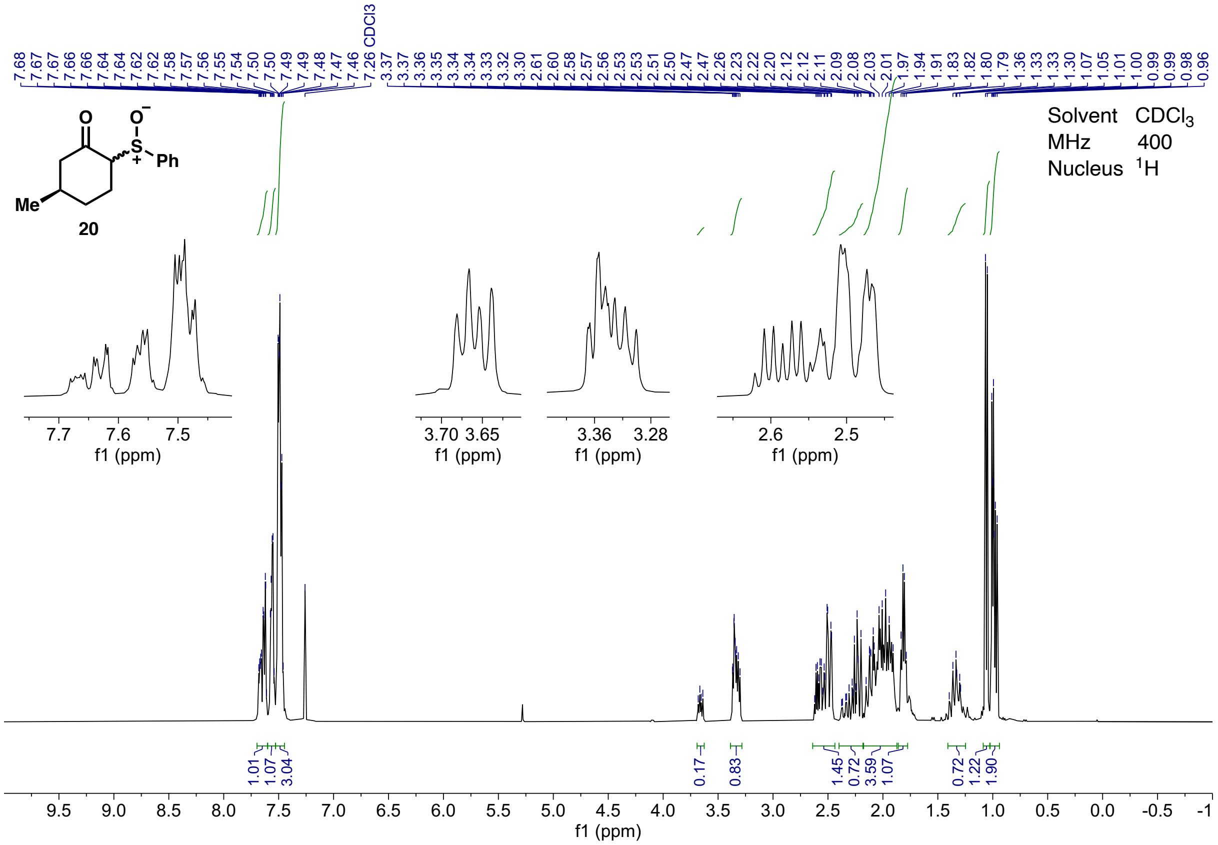
20
Solvent CDCl3
MHz 400
Nucleus 1H
f1 (ppm)

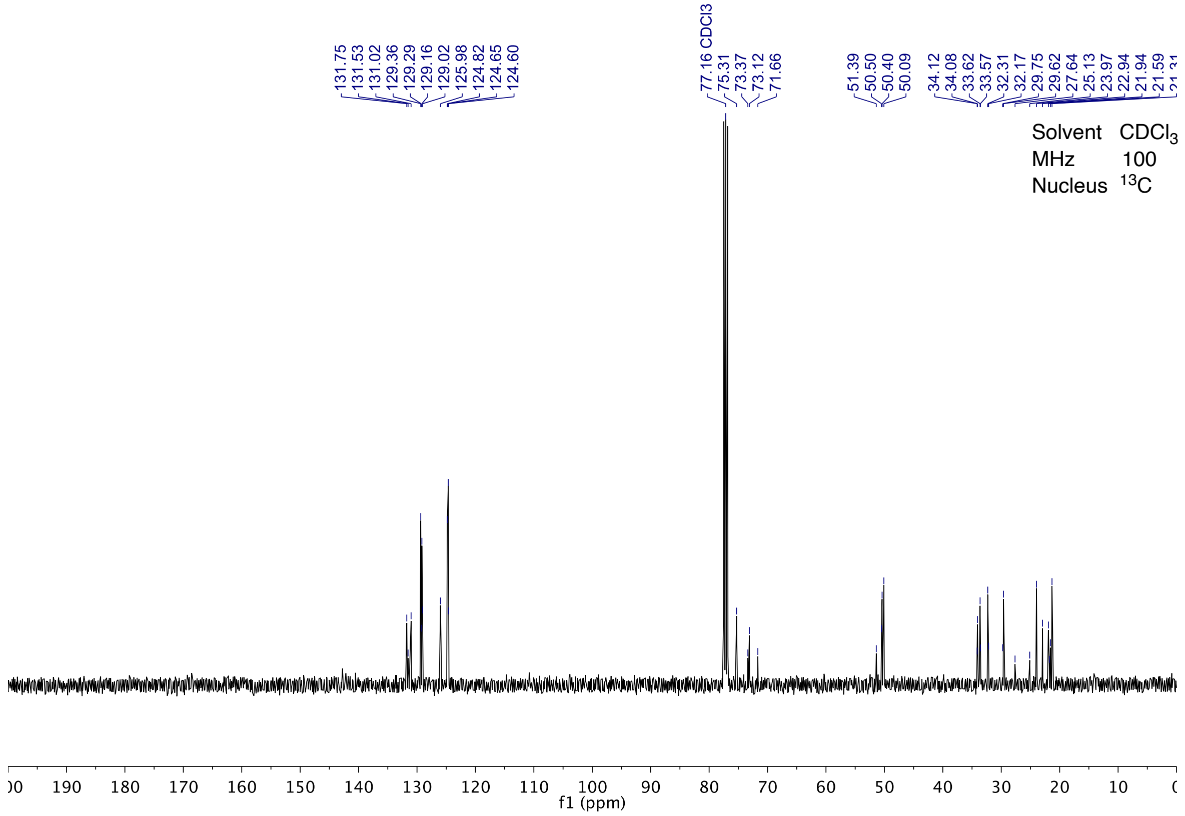
Solvent CDCl3
MHz 100
Nucleus 13C
f1 (ppm)

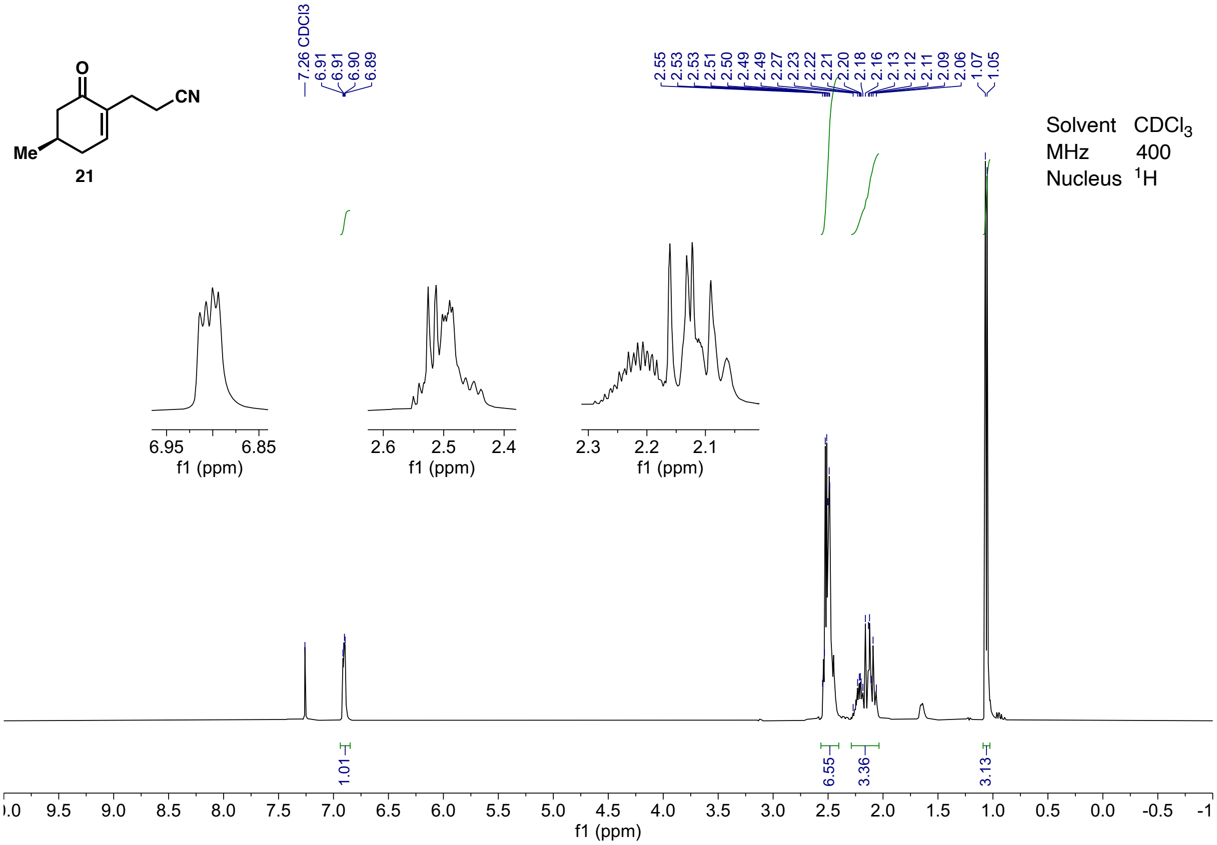
O
CN
Me
21
7.26 CDCl3
6.91
6.91
6.90
6.89
2.55
2.53
2.53
2.51
2.50
2.49
2.49
2.27
2.23
2.22
2.21
2.20
2.18
2.16
2.13
2.12
2.11
2.09
2.06
1.07
1.05
Solvent CDCl3
MHz 400
Nucleus 1H
6.95
6.85
f1 (ppm)
2.6
2.5
2.4
f1 (ppm)
2.3
2.2
2.1
f1 (ppm)
1.01
6.55
3.36
3.13
9.5
9.0
8.5
8.0
7.5
7.0
6.5
6.0
5.5
5.0
4.5
4.0
3.5
3.0
2.5
2.0
1.5
1.0
0.5
0.0
-0.5
f1 (ppm)

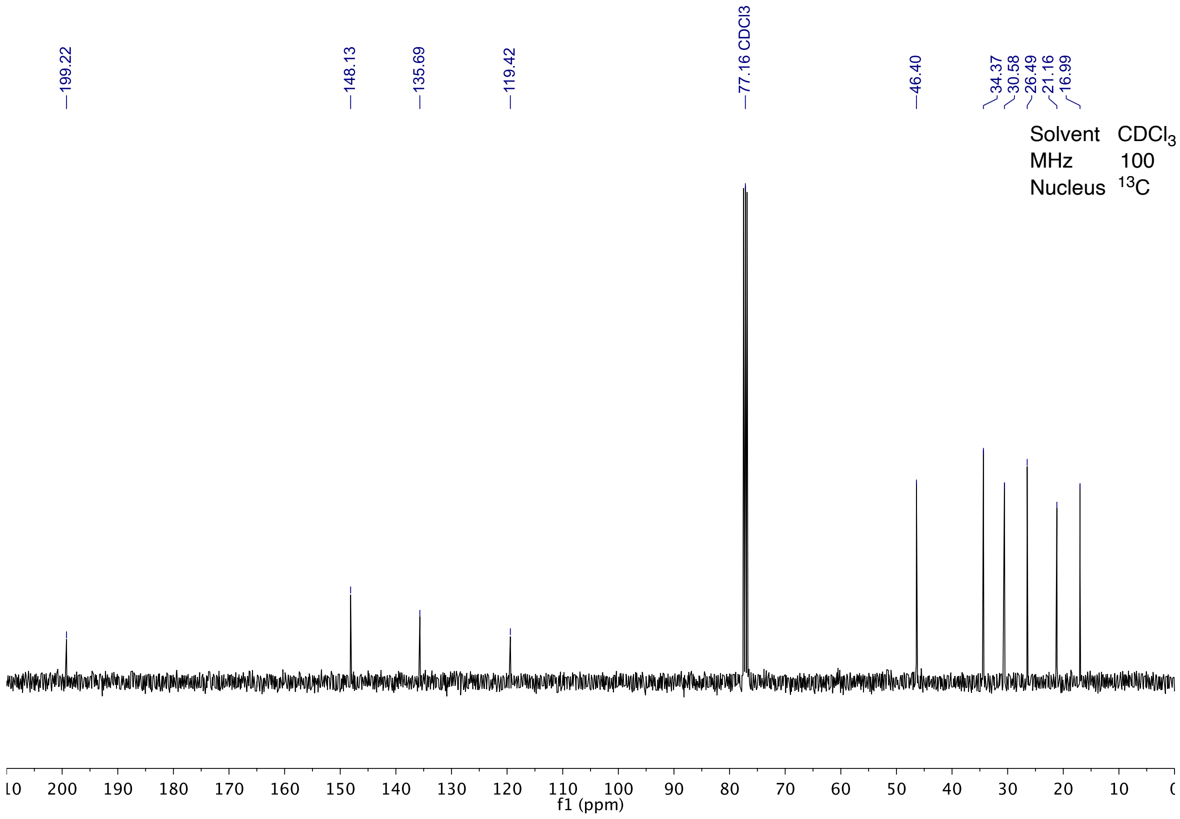
199.22
148.13
135.69
119.42
77.16 CDCl3
46.40
34.37
30.58
26.49
21.16
16.99
Solvent CDCl3
MHz 100
Nucleus 13C
200
190
180
170
160
150
140
130
120
110
100
90
80
70
60
50
40
30
20
10
f1 (ppm)

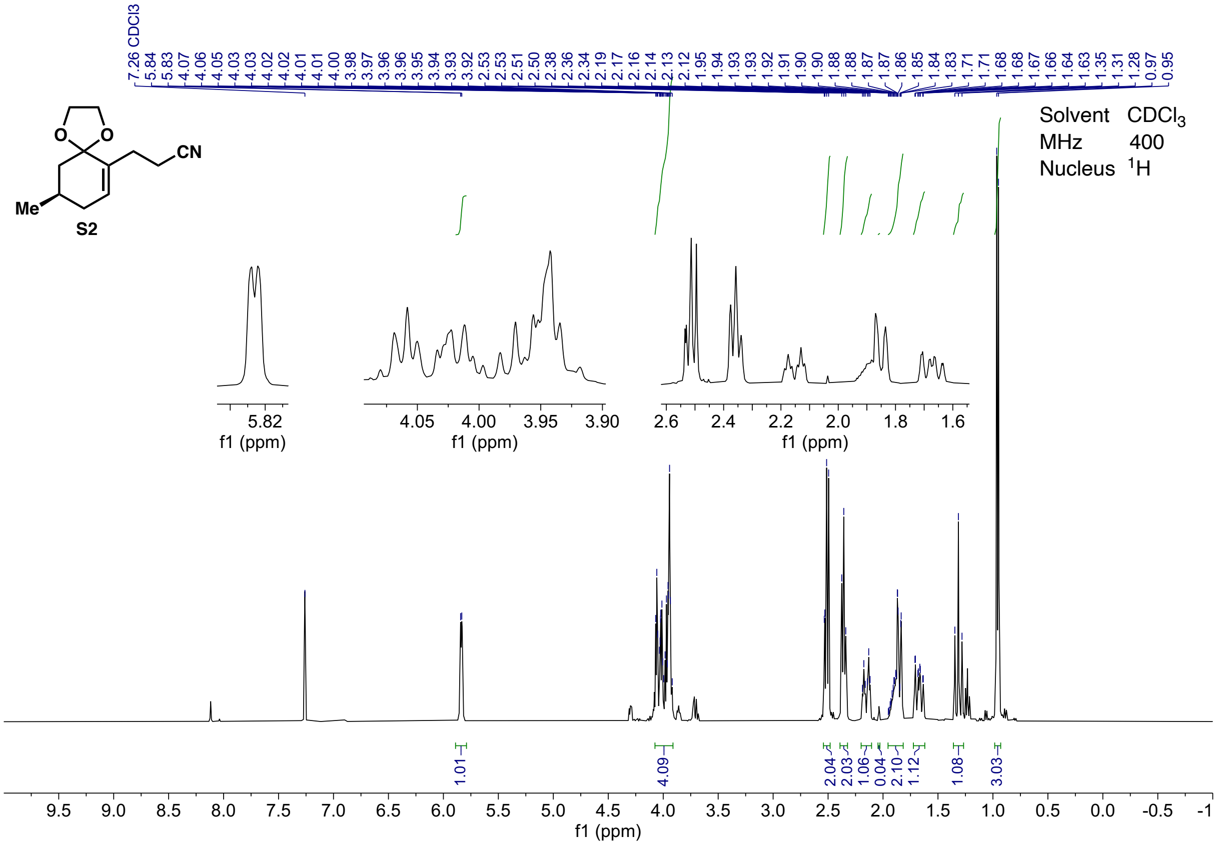

O
O
CN
Me
S2
7.26 CDCl3
5.84
5.83
4.07
4.06
4.05
4.03
4.03
4.02
4.02
4.01
4.01
4.00
3.98
3.97
3.96
3.96
3.95
3.94
3.93
3.93
3.92
2.53
2.53
2.51
2.50
2.38
2.36
2.34
2.19
2.17
2.16
2.14
2.13
2.12
1.95
1.94
1.93
1.93
1.92
1.91
1.90
1.90
1.88
1.88
1.87
1.87
1.86
1.85
1.84
1.83
1.71
1.68
1.68
1.67
1.66
1.64
1.63
1.35
1.31
1.28
0.97
0.95
Solvent CDCl3
MHz 400
Nucleus 1H
1.01
4.09
2.04
2.03
1.06
0.04
2.10
1.12
1.08
3.03
f1 (ppm)

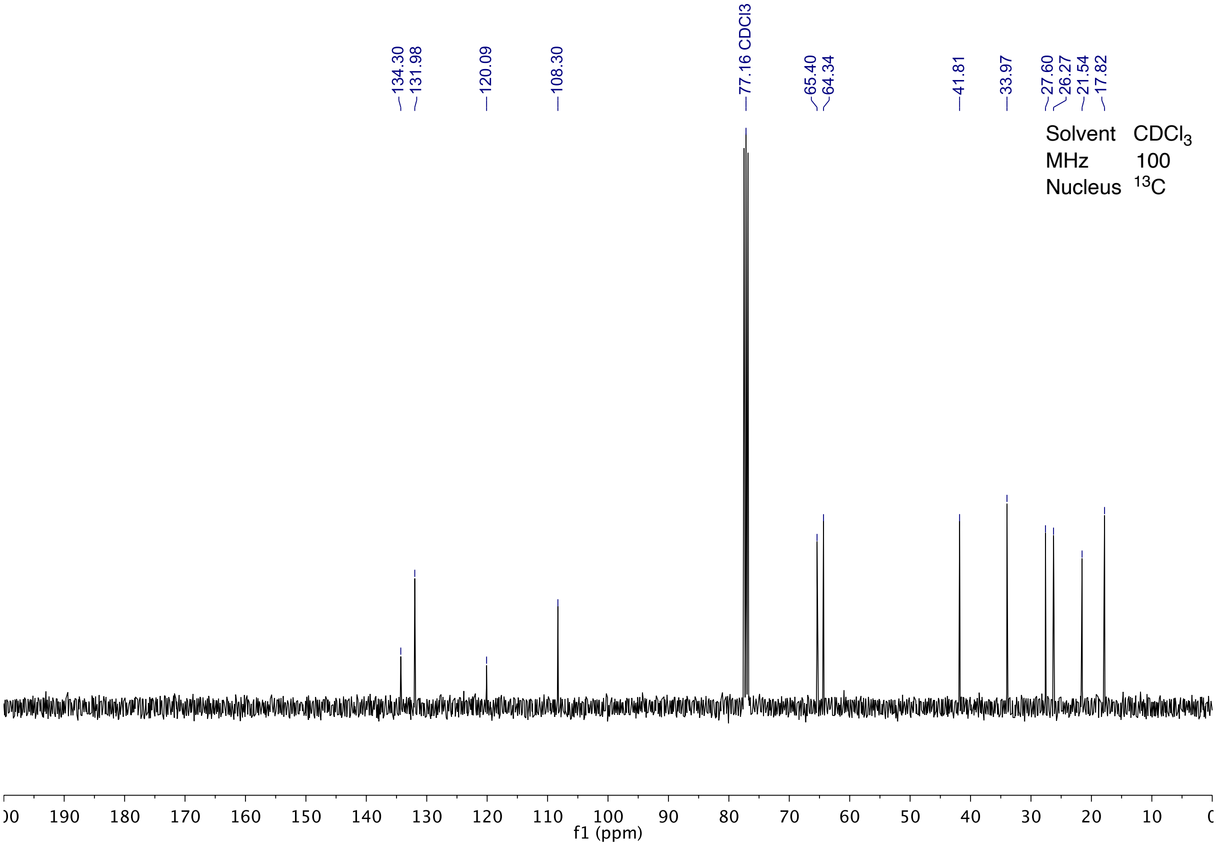

134.30
131.98
120.09
108.30
77.16 CDCl3
65.40
64.34
41.81
33.97
27.60
26.27
21.54
17.82
Solvent CDCl3
MHz 100
Nucleus 13C
f1 (ppm)

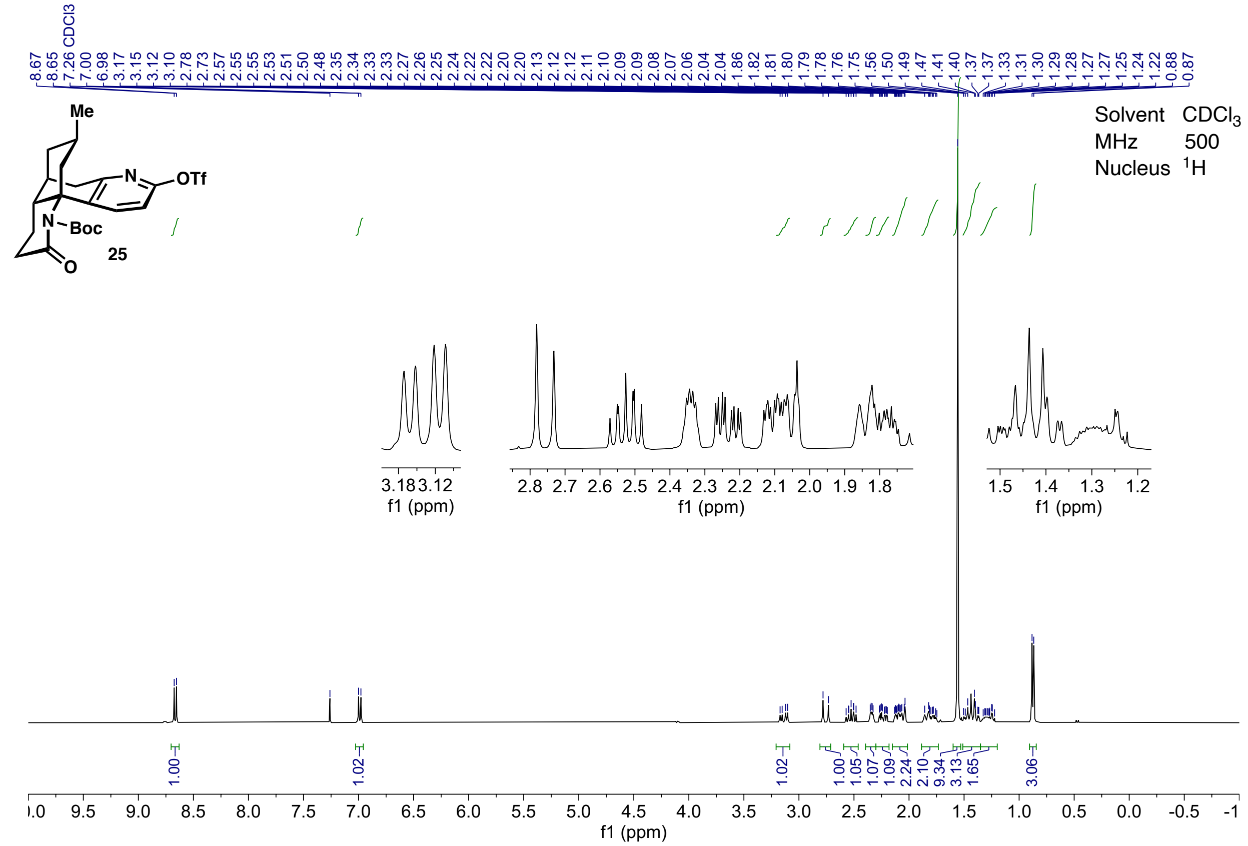
8.67 8.65 7.26 CDCl3 7.00 6.98 3.17 3.15 3.12 3.10 2.78 2.73 2.57 2.55 2.55 2.53 2.51 2.50 2.48 2.35 2.34 2.33 2.33 2.27 2.26 2.25 2.24 2.22 2.22 2.20 2.20 2.13 2.12 2.12 2.11 2.10 2.09 2.09 2.08 2.07 2.06 2.04 2.04 1.86 1.82 1.81 1.80 1.79 1.78 1.76 1.75 1.56 1.50 1.49 1.47 1.41 1.40 1.37 1.37 1.33 1.31 1.30 1.29 1.28 1.27 1.27 1.25 1.24 1.22 0.88 0.87
Me
N
OTf
N
Boc
O
25
Solvent CDCl3
MHz 500
Nucleus 1H
f1 (ppm)
1.00
1.02
1.02
1.00
1.05
1.07
1.09
2.24
2.10
9.34
3.13
1.65
3.06

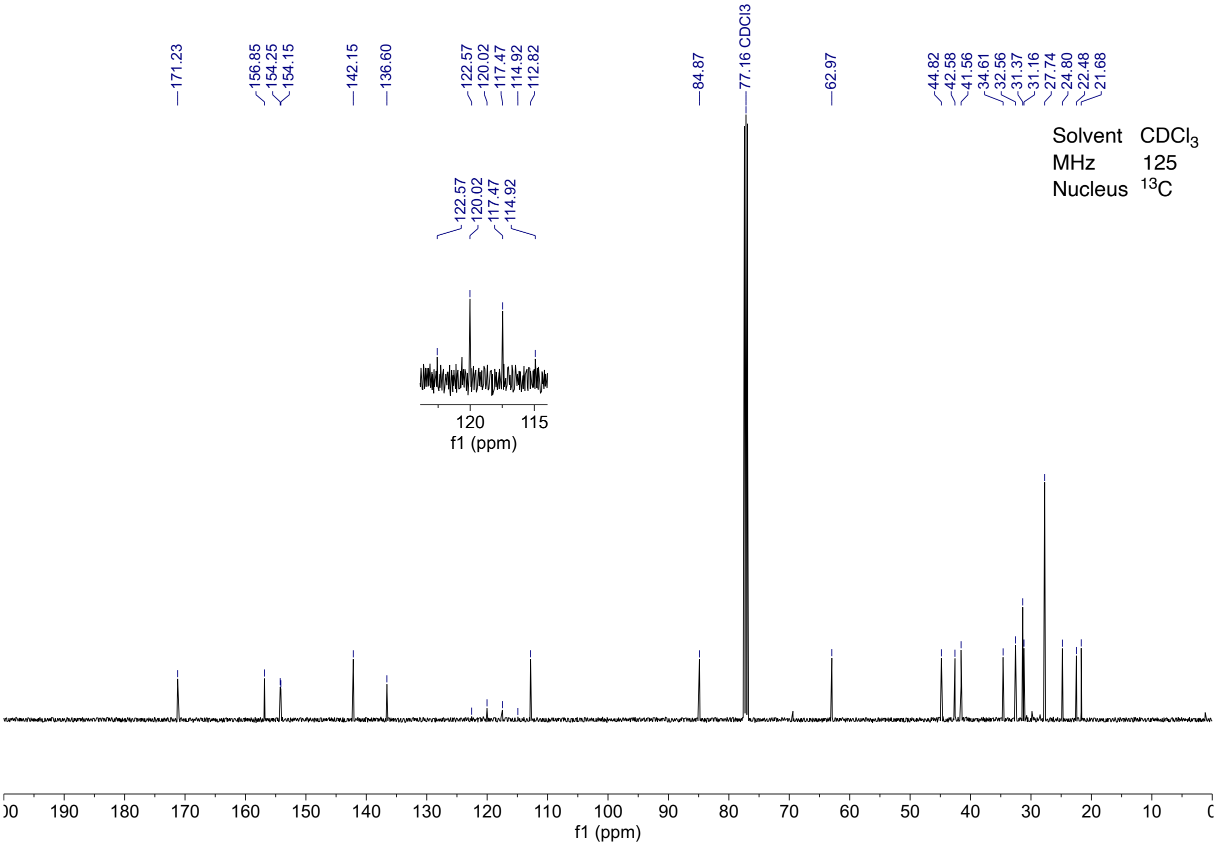
171.23
156.85 154.25 154.15
142.15
136.60
122.57 120.02 117.47 114.92 112.82
84.87
77.16 CDCl3
62.97
44.82 42.58 41.56 34.61 32.56 31.37 31.16 27.74 24.80 22.48 21.68
Solvent CDCl3
MHz 125
Nucleus 13C
f1 (ppm)

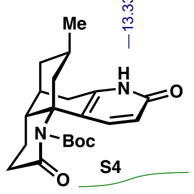
S4

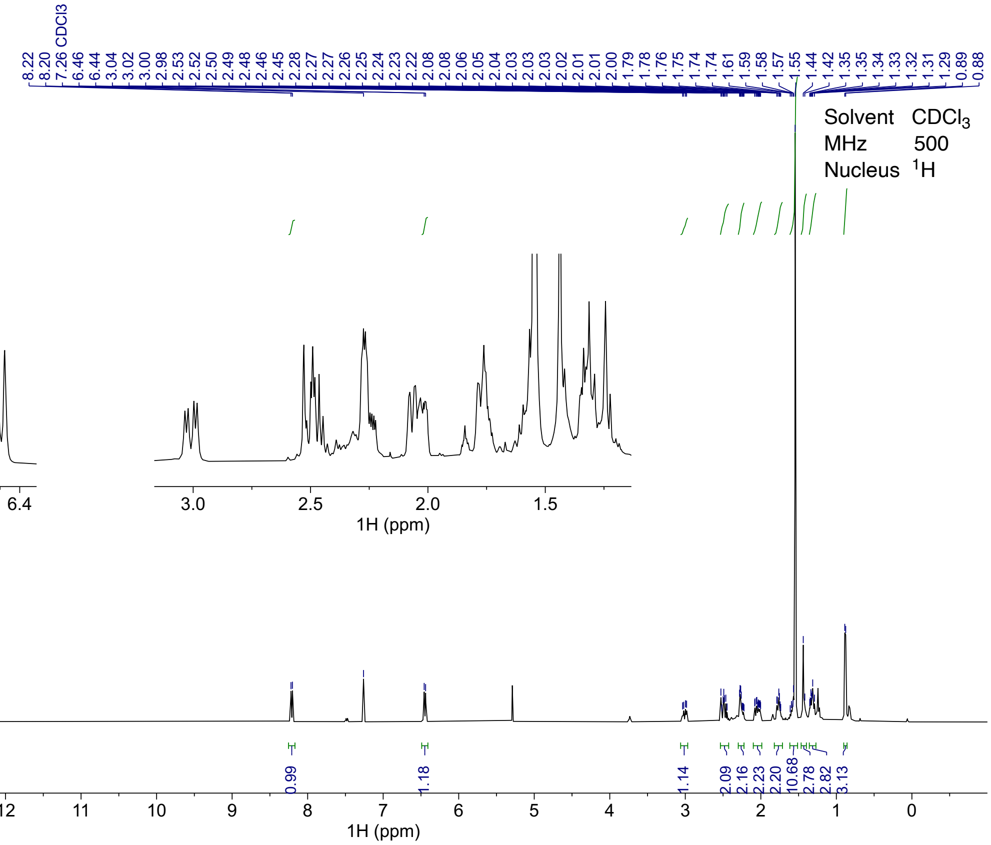
13.33
8.22 8.20 7.26 CDCl3 6.46 6.44 3.04 3.02 3.00 2.98 2.53 2.52 2.50 2.49 2.48 2.46 2.45 2.28 2.27 2.27 2.26 2.25 2.24 2.23 2.22 2.08 2.08 2.06 2.05 2.04 2.03 2.03 2.03 2.02 2.01 2.01 2.00 1.79 1.78 1.76 1.75 1.74 1.74 1.61 1.59 1.58 1.57 1.55 1.44 1.42 1.35 1.35 1.34 1.33 1.32 1.31 1.29 0.89 0.88
Solvent CDCl3
MHz 500
Nucleus 1H
8.2 8.1 6.5 6.4
1H (ppm)
3.0 2.5 2.0 1.5
1H (ppm)
0.97 0.99 1.18 1.14 2.09 2.16 2.23 2.20 10.68 2.78 2.82 3.13
1H (ppm)

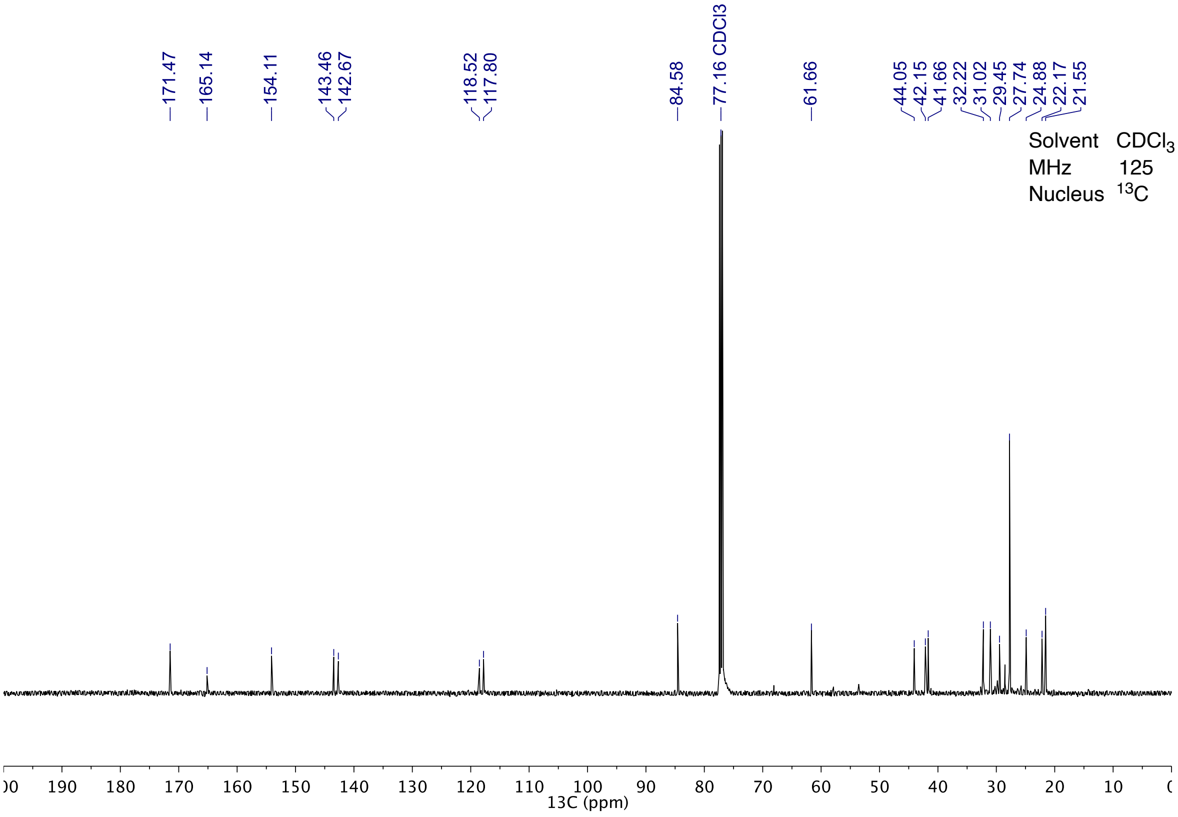
171.47 165.14 154.11 143.46 142.67 118.52 117.80 84.58 77.16 CDCl3 61.66 44.05 42.15 41.66 32.22 31.02 29.45 27.74 24.88 22.17 21.55
Solvent CDCl3
MHz 125
Nucleus 13C
13C (ppm)

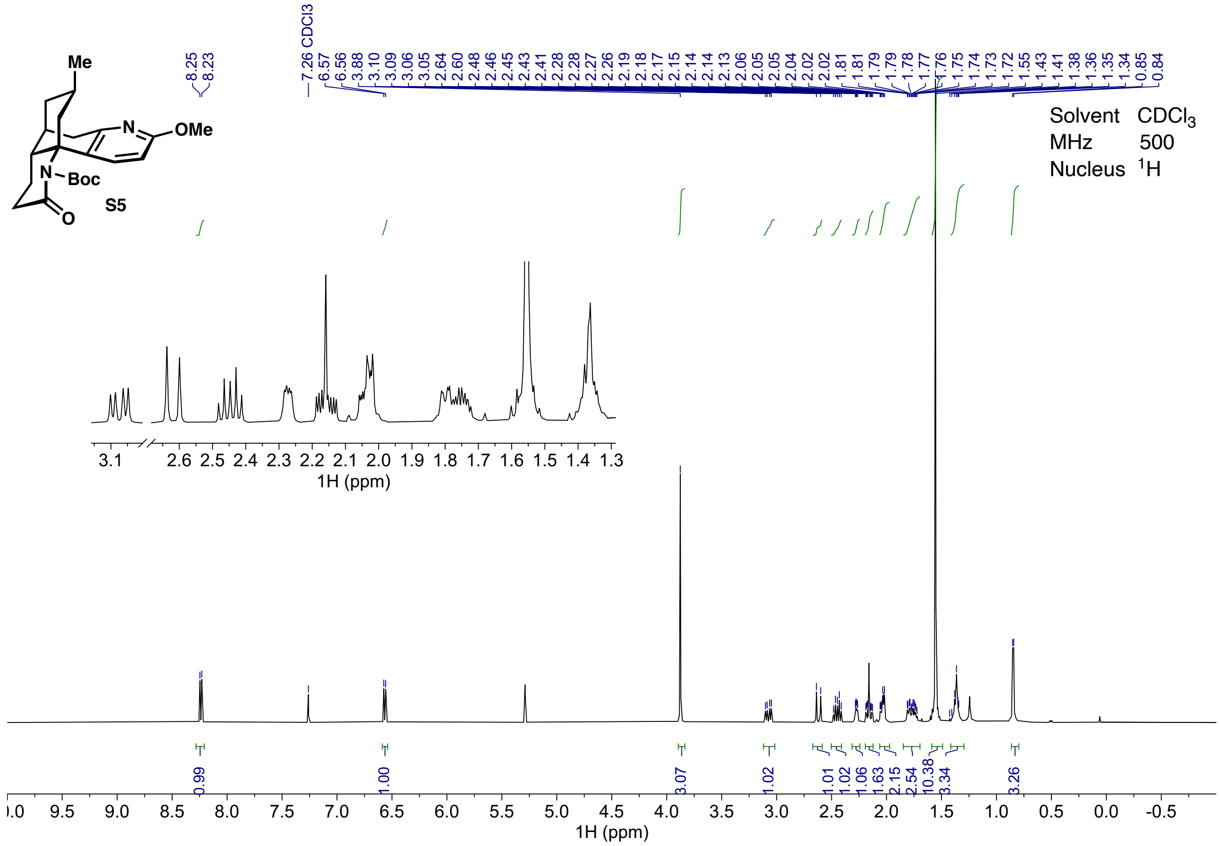

Me
N
OMe
N
Boc
O
S5
8.25
8.23
7.26 CDCl3
6.57
6.56
3.88
3.10
3.09
3.06
3.05
2.64
2.60
2.48
2.46
2.45
2.43
2.41
2.28
2.28
2.27
2.26
2.19
2.18
2.17
2.15
2.14
2.13
2.06
2.05
2.04
2.02
2.02
1.81
1.81
1.79
1.78
1.77
1.76
1.75
1.74
1.73
1.72
1.55
1.43
1.41
1.38
1.36
1.35
1.34
0.85
0.84
Solvent CDCl3
MHz 500
Nucleus 1H
0.99
1.00
3.07
1.02
1.01
1.02
1.06
1.63
2.15
2.54
10.38
3.34
3.26
1H (ppm)

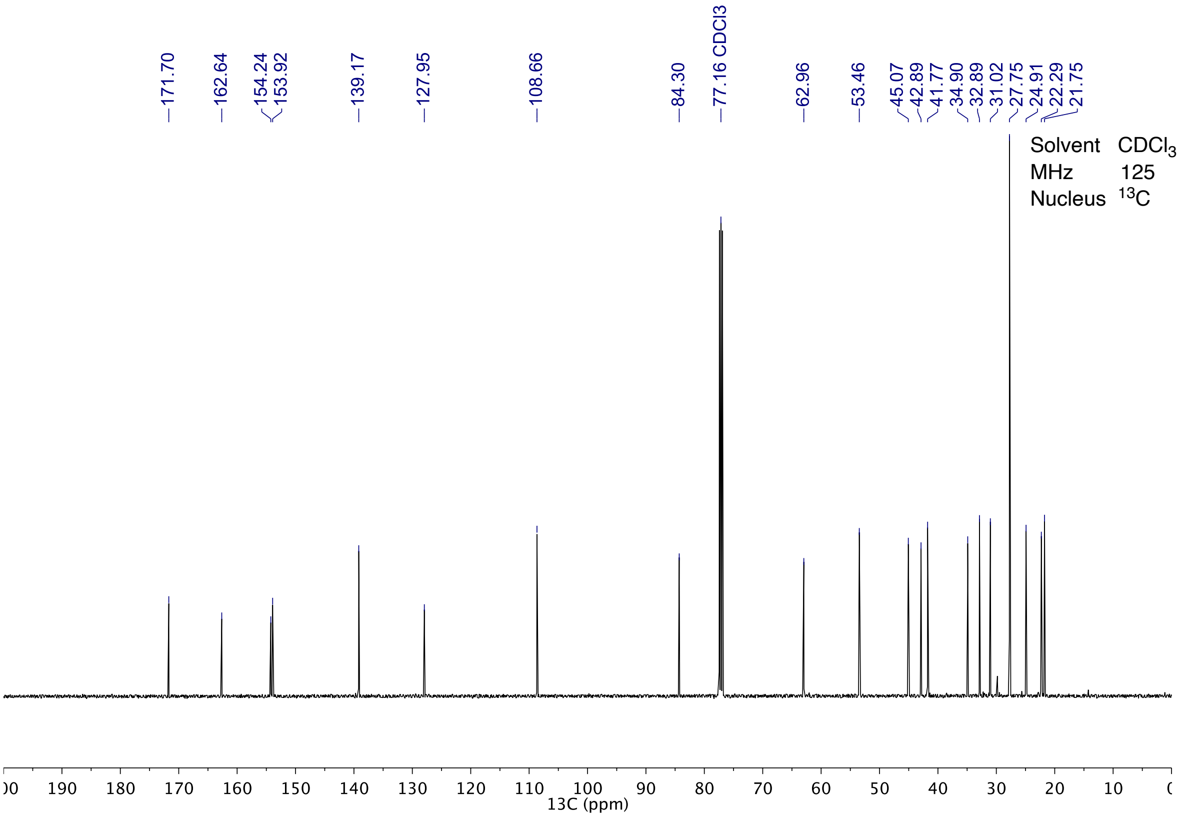

171.70
162.64
154.24
153.92
139.17
127.95
108.66
84.30
77.16 CDCl3
62.96
53.46
45.07
42.89
41.77
34.90
32.89
31.02
27.75
24.91
22.29
21.75
Solvent CDCl3
MHz 125
Nucleus 13C
13C (ppm)

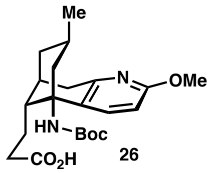
Me
N
OMe
HN
Boc
CO2H
26

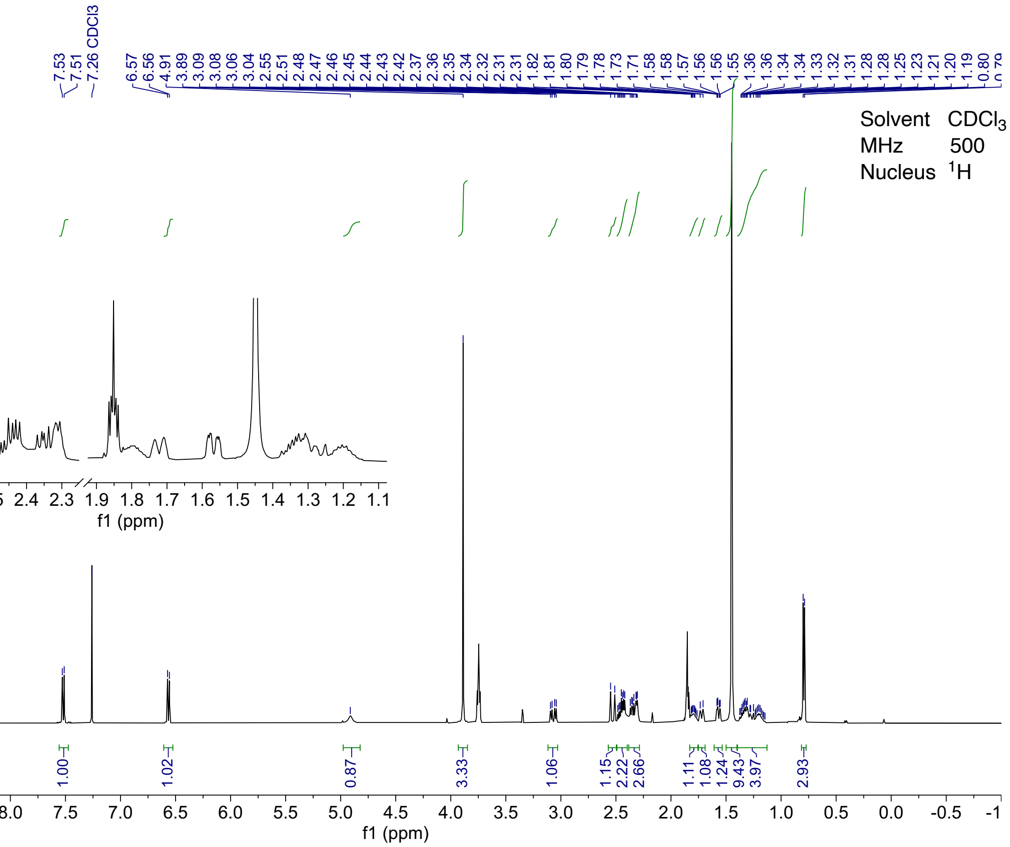
Solvent CDCl3
MHz 500
Nucleus 1H
7.26 CDCl3
f1 (ppm)

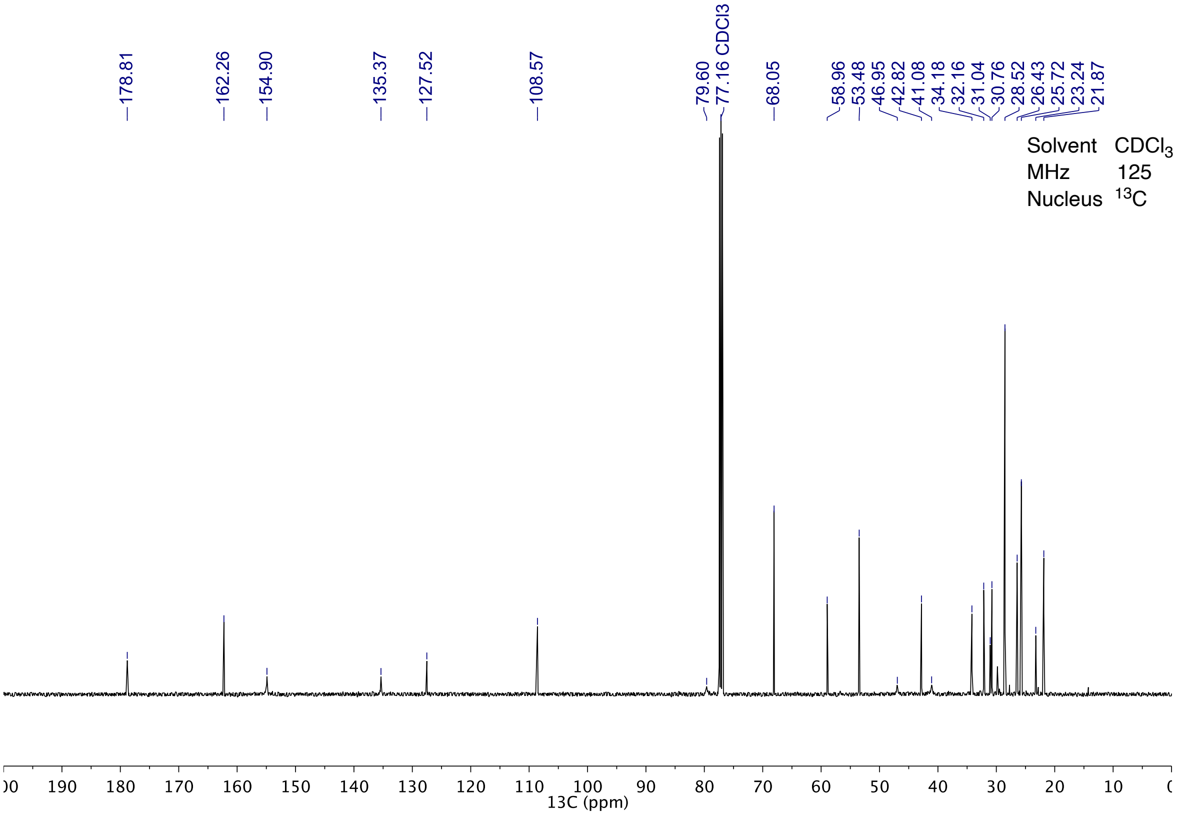
178.81
162.26
154.90
135.37
127.52
108.57
79.60
77.16 CDCl3
68.05
58.96
53.48
46.95
42.82
41.08
34.18
32.16
31.04
30.76
28.52
26.43
25.72
23.24
21.87
Solvent CDCl3
MHz 125
Nucleus 13C
13C (ppm)

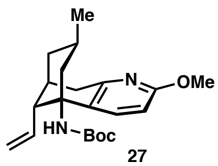

27

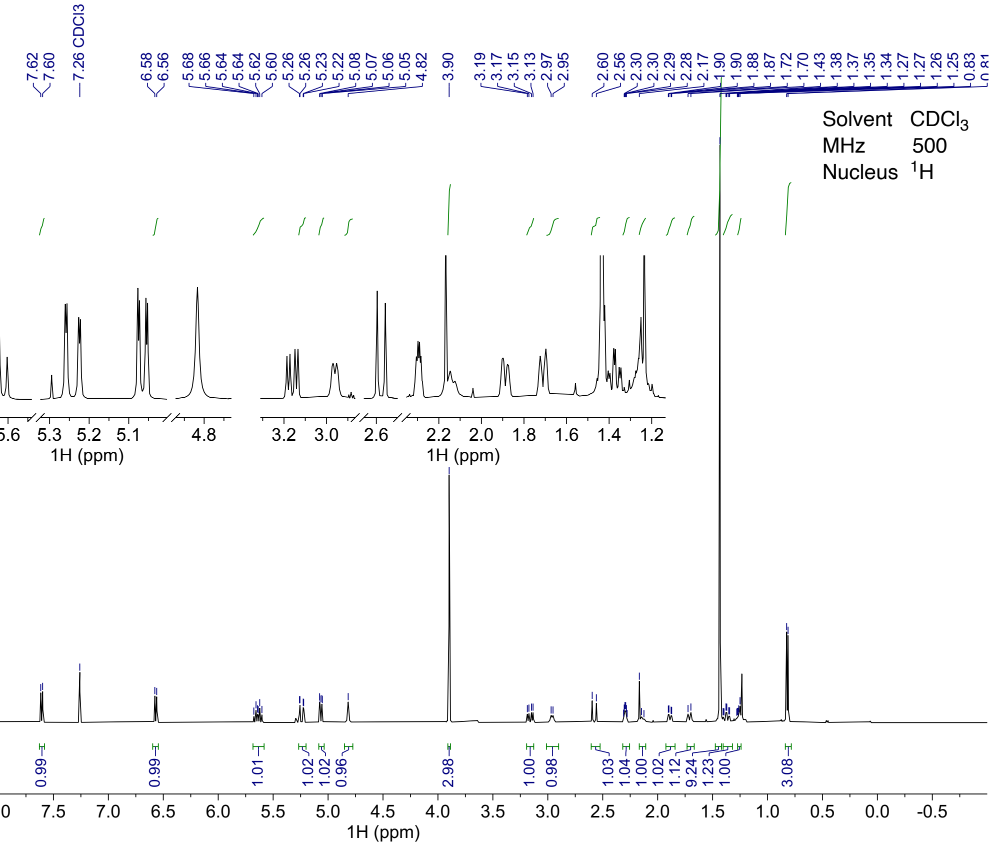

Solvent CDCl3
MHz 500
Nucleus 1H
1H (ppm)

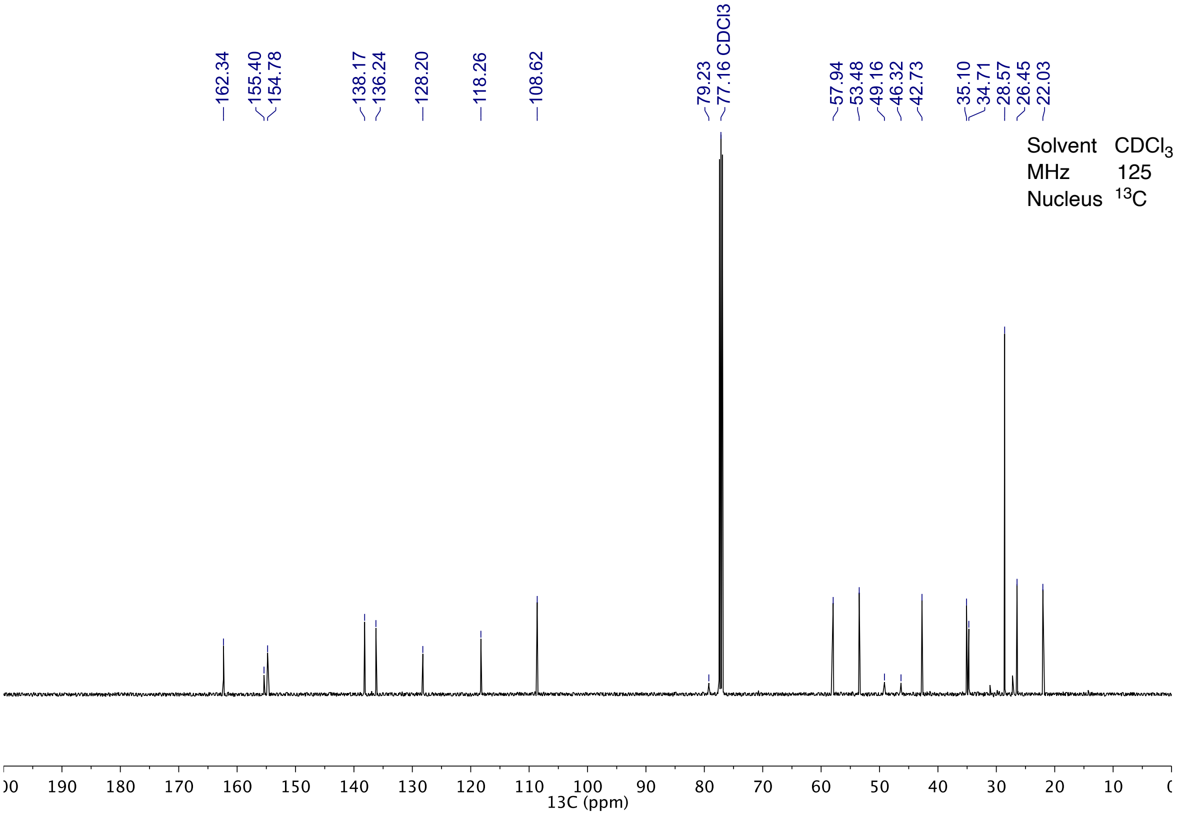

Solvent CDCl3
MHz 125
Nucleus 13C
13C (ppm)

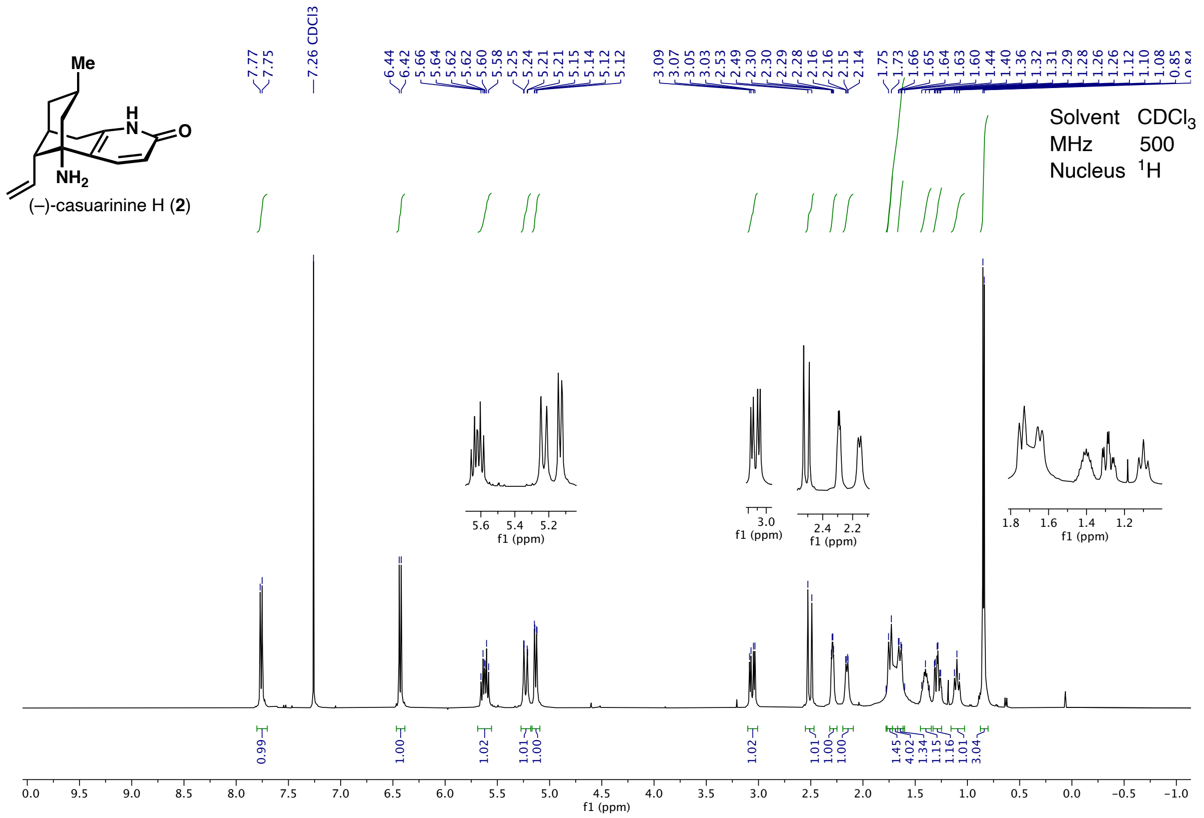
Me
H
N
O
NH2
(–)-casuarinine H (2)
Solvent CDCl3
MHz 500
Nucleus 1H
f1 (ppm)

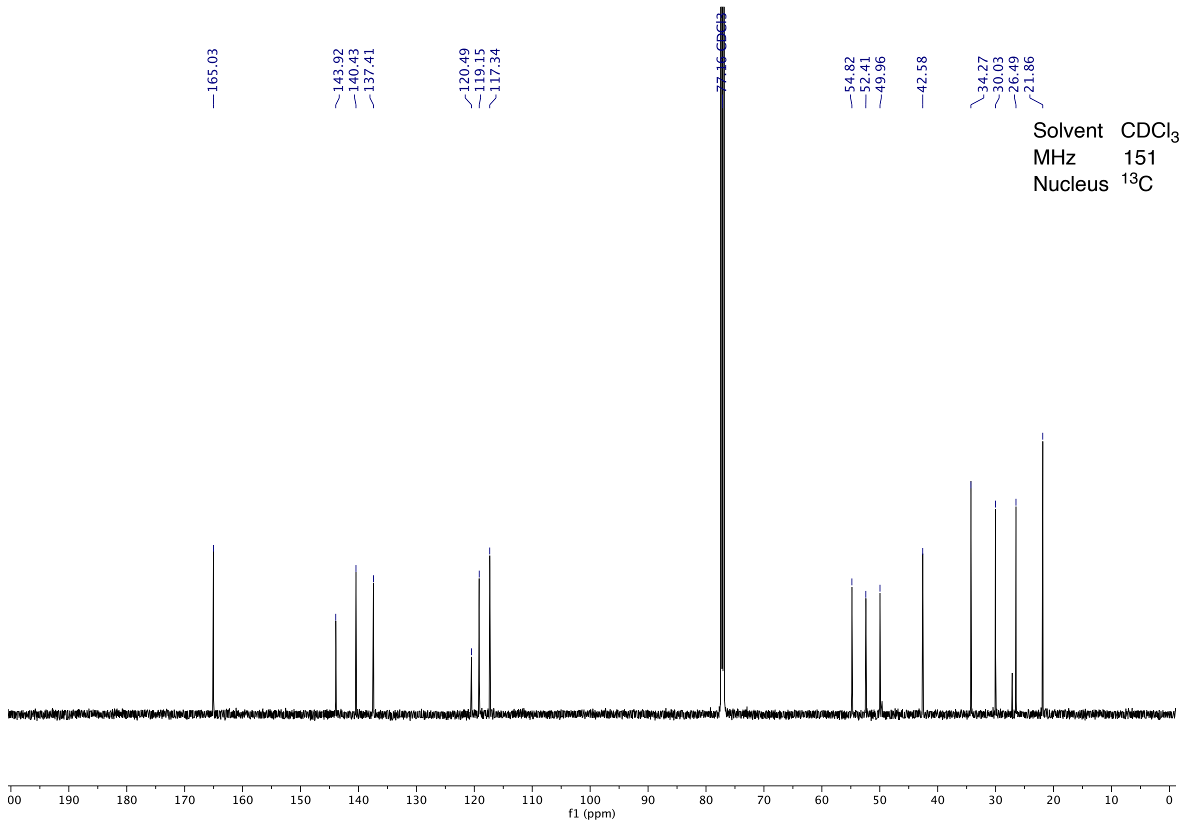
Solvent CDCl3
MHz 151
Nucleus 13C
f1 (ppm)

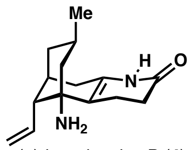

(+)-lycoplatyrine B (**4**)

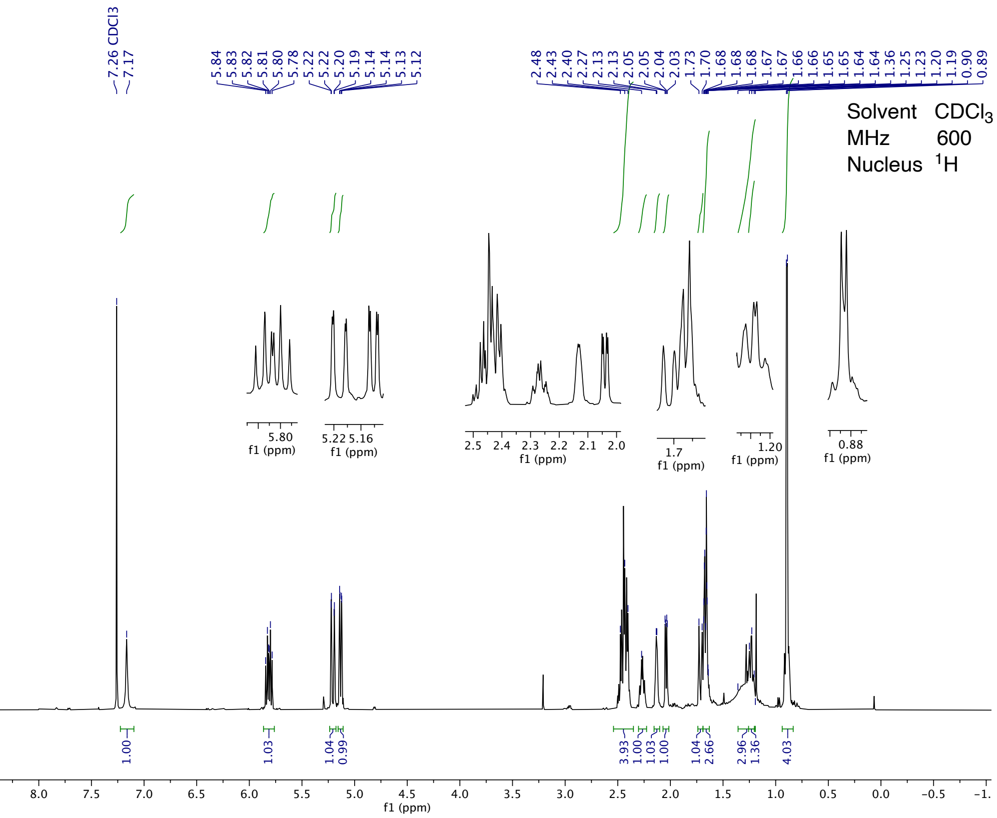

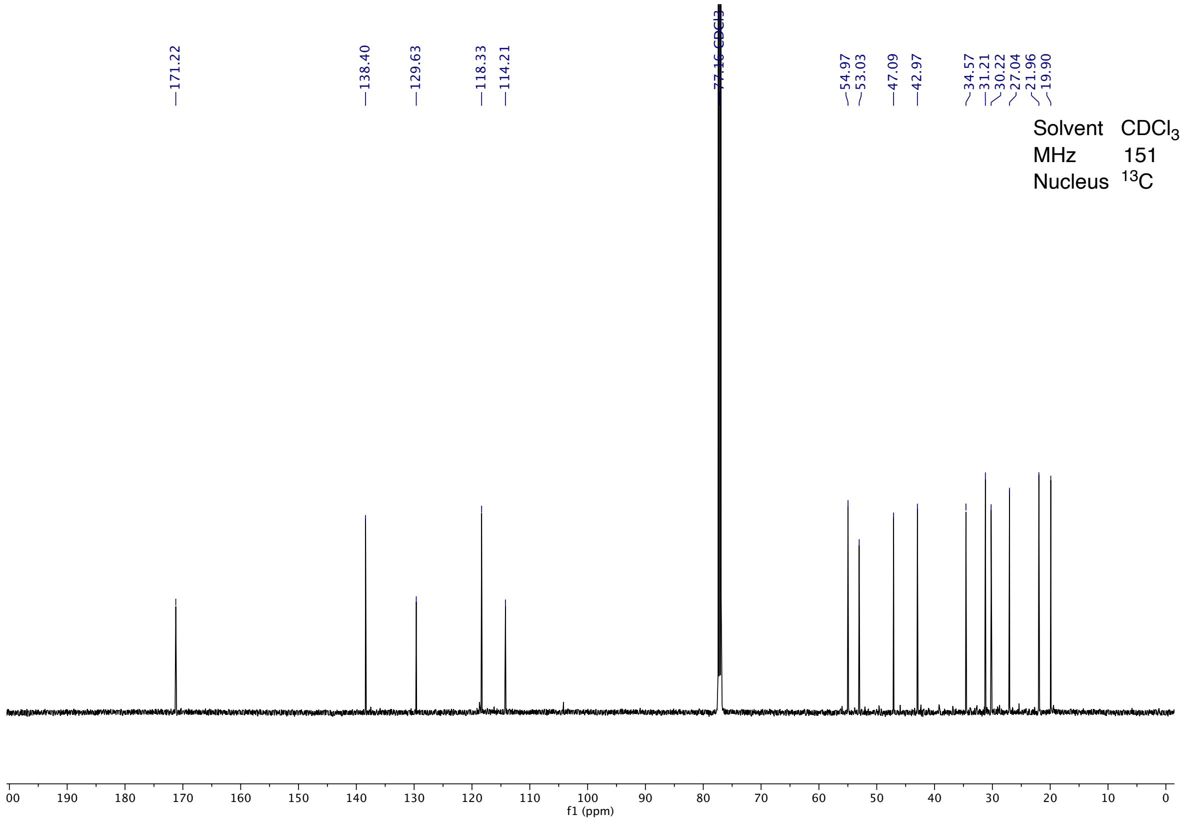

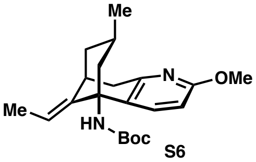
Me
N
OMe
Me
HN
Boc

S6

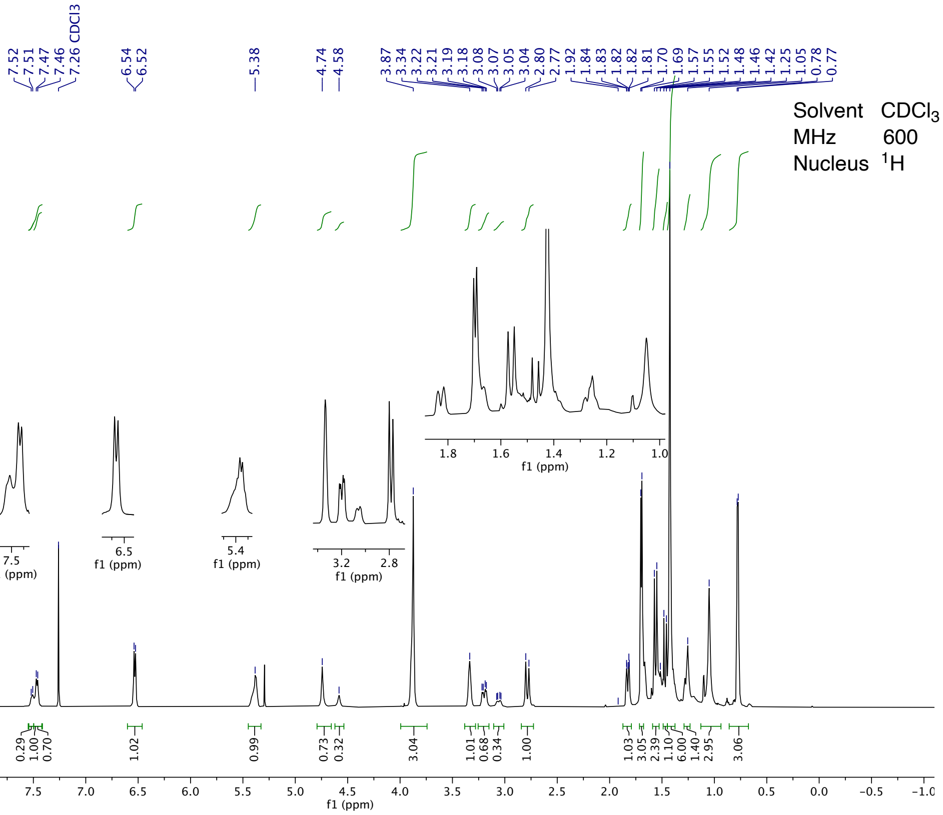
7.52
7.51
7.47
7.46
7.26 CDCl3
6.54
6.52
5.38
4.74
4.58
3.87
3.34
3.22
3.21
3.19
3.18
3.08
3.07
3.05
3.04
2.80
2.77
1.92
1.84
1.83
1.82
1.82
1.81
1.70
1.69
1.57
1.55
1.52
1.48
1.46
1.42
1.25
1.05
0.78
0.77
Solvent CDCl3
MHz 600
Nucleus 1H
f1 (ppm)

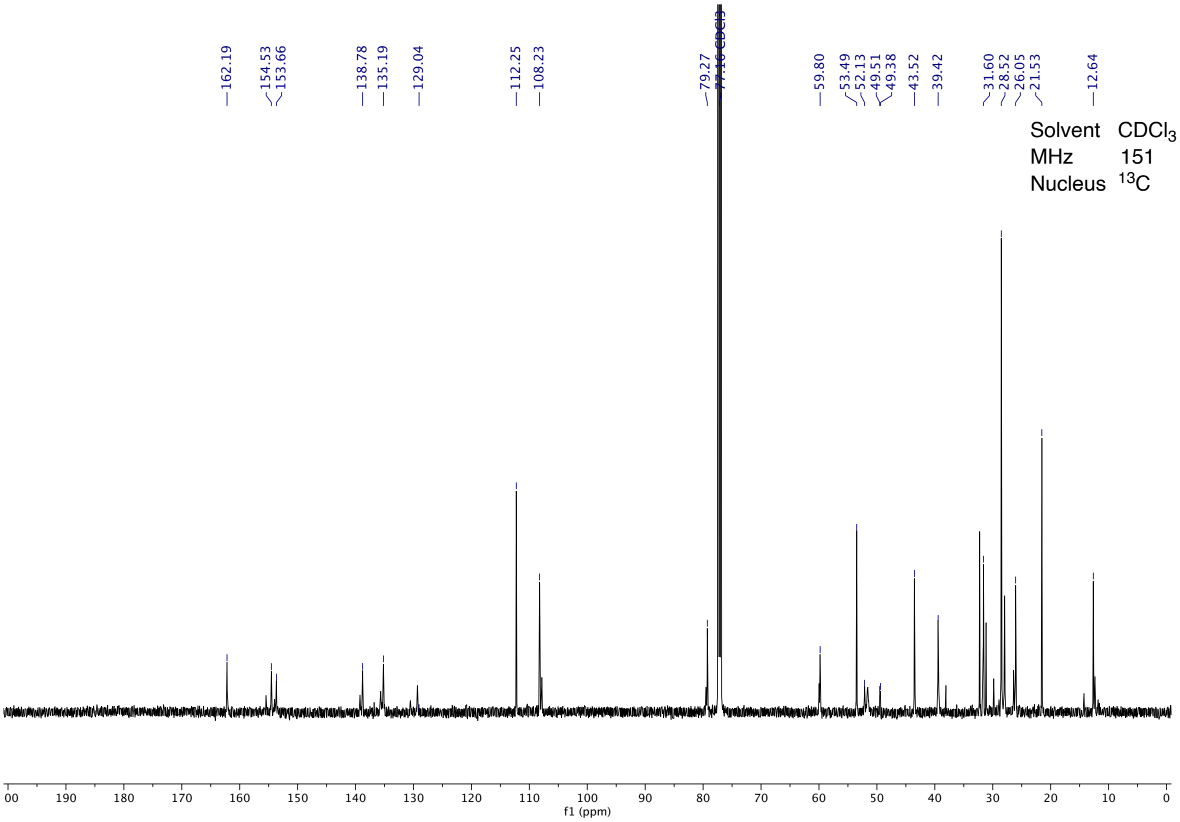
162.19
154.53
153.66
138.78
135.19
129.04
112.25
108.23
79.27
77.16 CDCl3
59.80
53.49
52.13
49.51
49.38
43.52
39.42
31.60
28.52
26.05
21.53
12.64
Solvent CDCl3
MHz 151
Nucleus 13C
f1 (ppm)

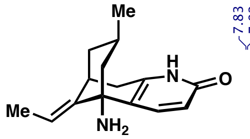
(–)-8,15-dihydrohuperzine A (**3**)

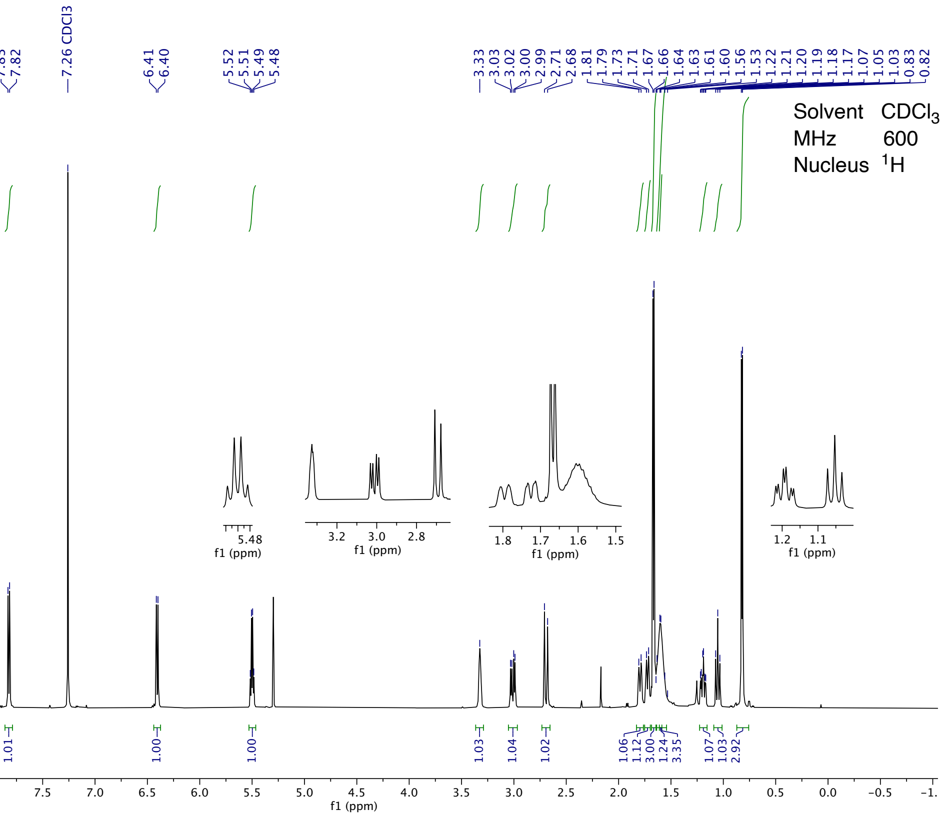

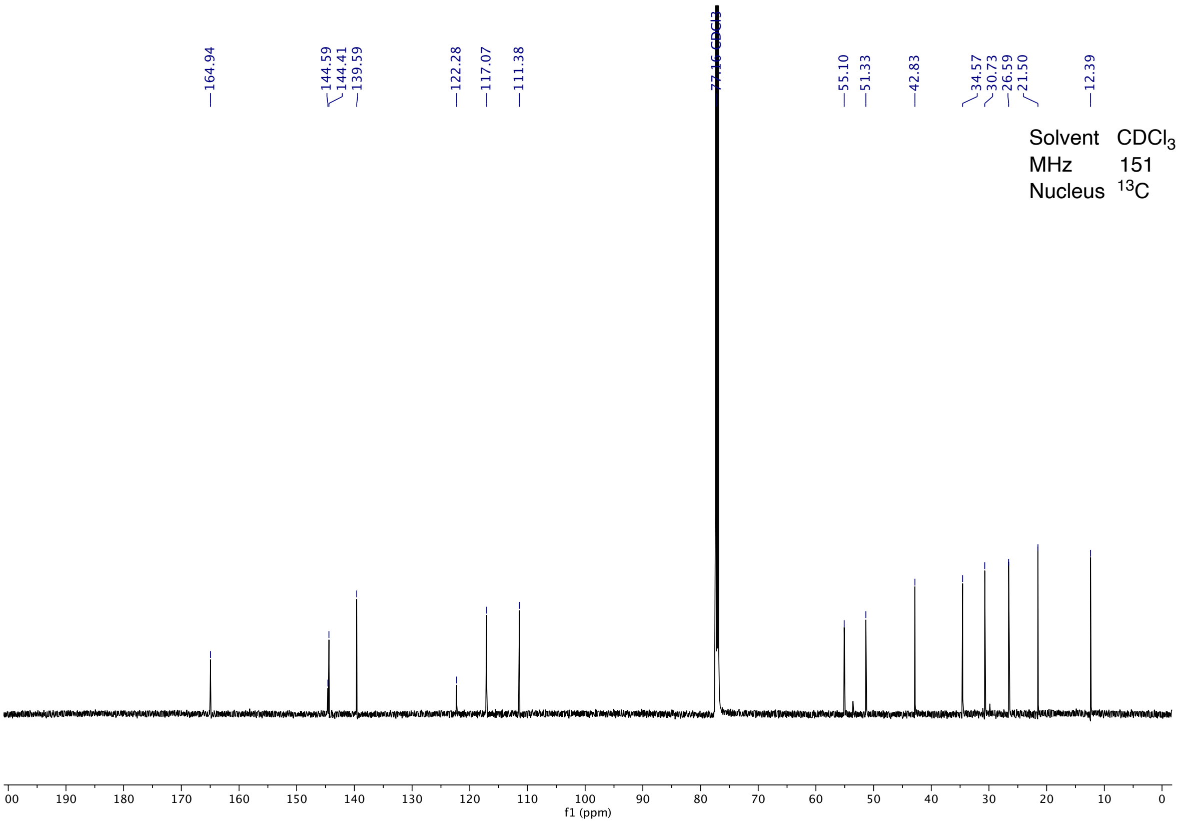

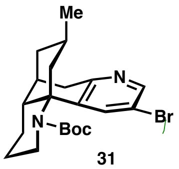
Me
N
Br
N
Boc

31

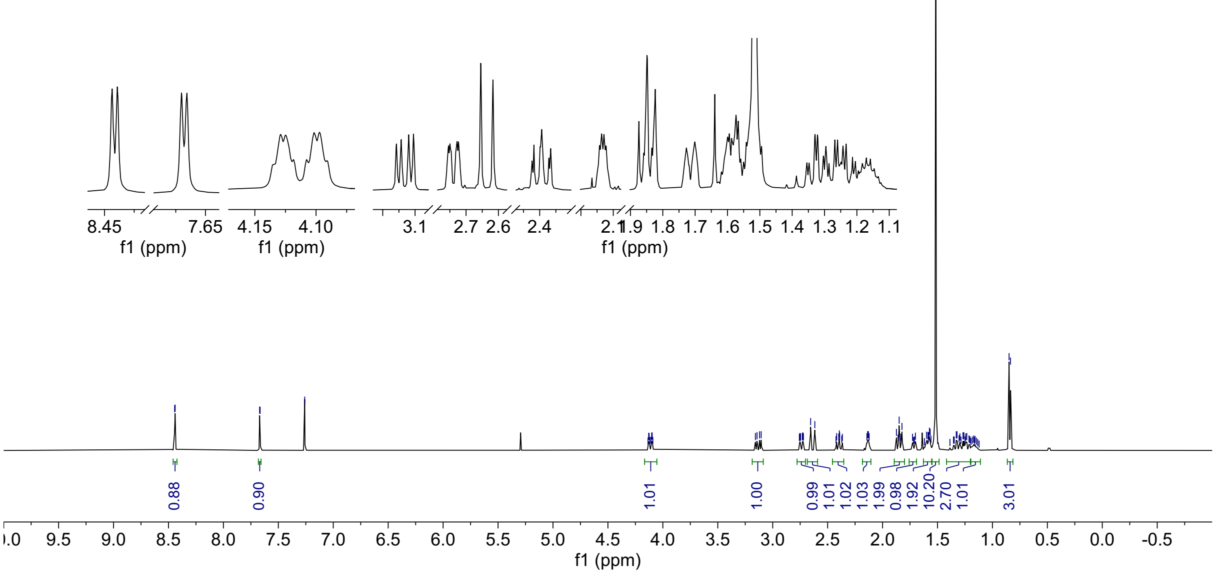
8.44
8.44
7.67
7.26 CDCl3
4.13
4.12
4.12
4.11
4.10
4.10
3.16
3.14
3.12
3.10
2.76
2.75
2.75
2.75
2.73
2.73
2.72
2.72
2.65
2.62
2.42
2.42
2.40
2.39
2.39
2.37
2.37
2.14
2.14
2.13
2.12
1.87
1.86
1.85
1.83
1.82
1.73
1.72
1.71
1.70
1.60
1.59
1.58
1.58
1.57
1.57
1.56
1.52
1.35
1.35
1.33
1.32
1.30
1.30
1.29
1.27
1.26
1.25
1.24
1.23
1.21
1.20
1.18
1.17
1.16
0.85
0.84
Solvent CDCl$_3$
MHz 500
Nucleus ^{1}H
8.45
7.65
f1 (ppm)
4.15
4.10
f1 (ppm)
3.1
2.7
2.6
2.4
2.1
1.9
1.8
1.7
1.6
1.5
1.4
1.3
1.2
1.1
f1 (ppm)
0.88
0.90
1.01
1.00
0.99
1.01
1.02
1.03
1.99
0.98
1.92
10.20
2.70
1.01
3.01
f1 (ppm)

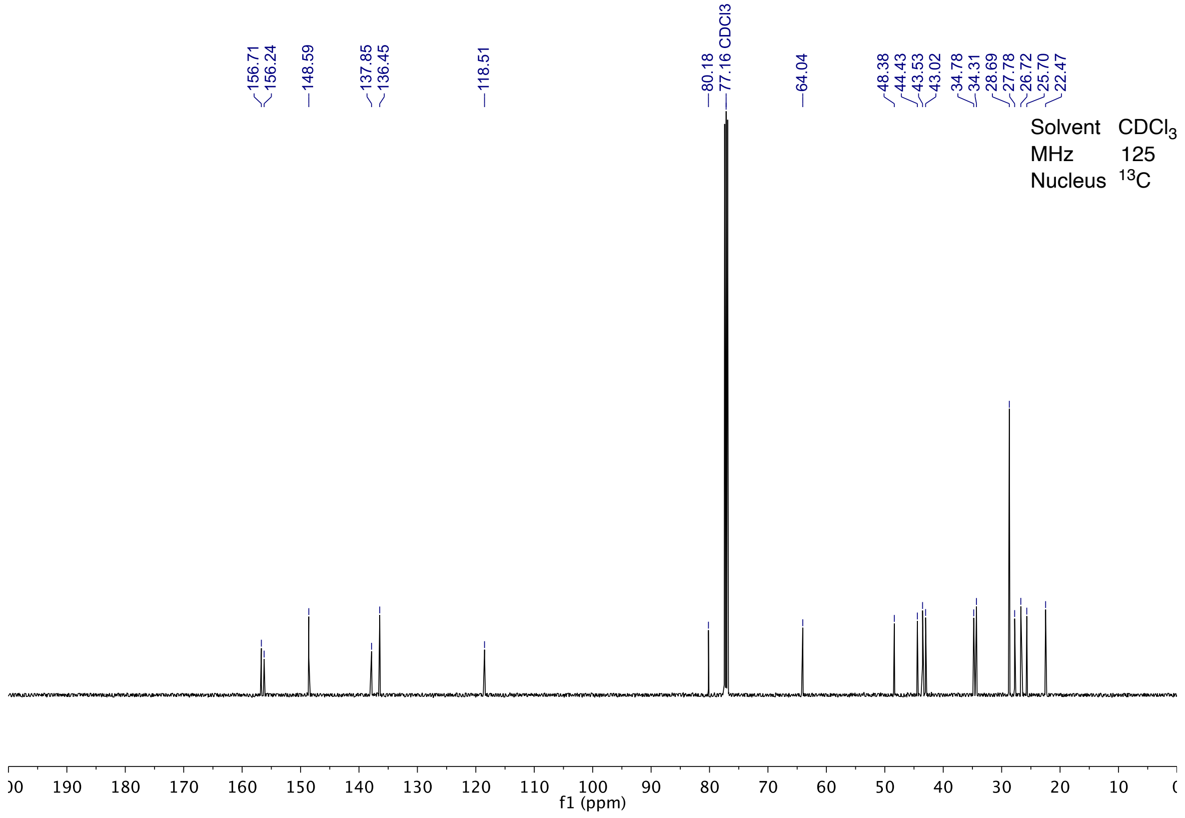
156.71
156.24
148.59
137.85
136.45
118.51
80.18
77.16 CDCl3
64.04
48.38
44.43
43.53
43.02
34.78
34.31
28.69
27.78
26.72
25.70
22.47
Solvent CDCl$_3$
MHz 125
Nucleus ^{13}C
f1 (ppm)

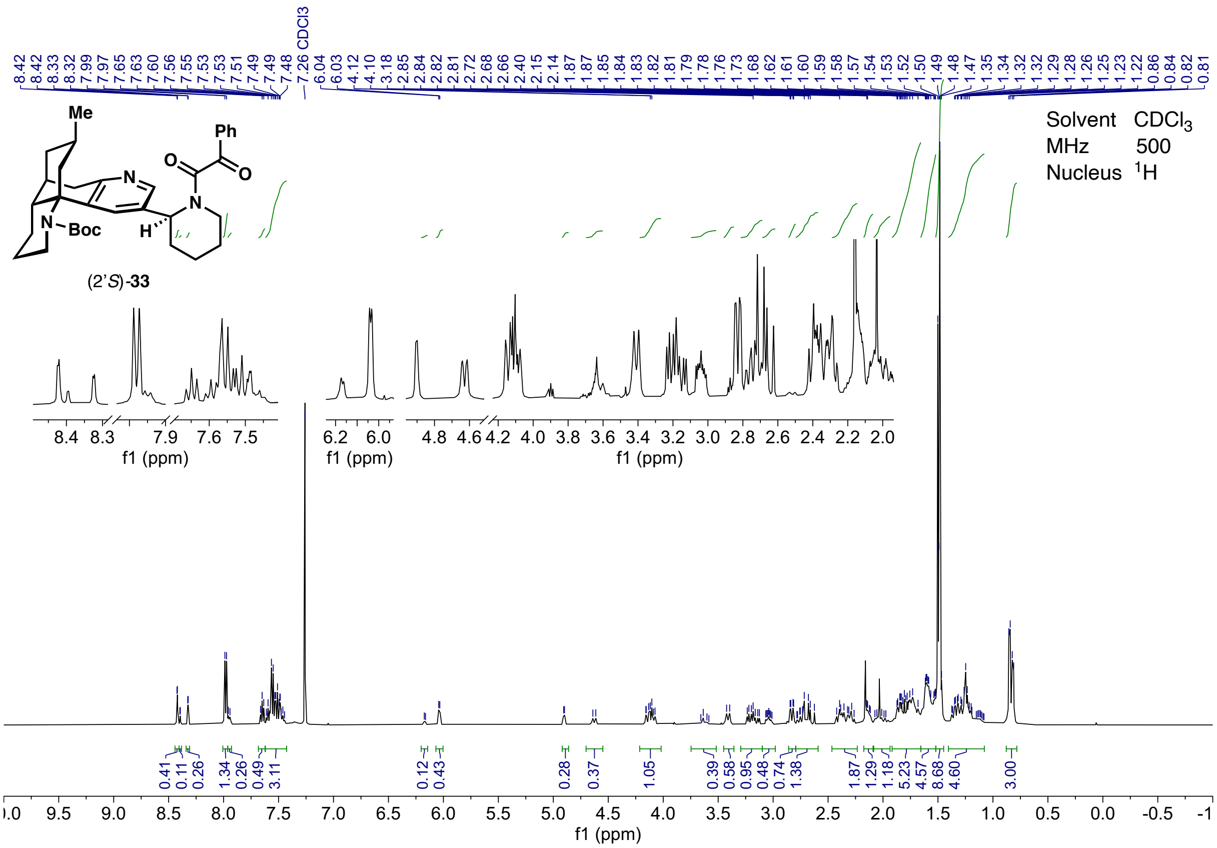
8.42 8.42 8.33 8.32 7.99 7.97 7.65 7.63 7.60 7.56 7.55 7.53 7.53 7.51 7.49 7.48
7.26 CDCl3
6.04 6.03 4.12 4.10 3.18 2.85 2.84 2.82 2.81 2.72 2.68 2.66 2.40 2.15 2.14 1.87 1.87 1.85 1.84 1.83 1.82 1.81 1.79 1.78 1.76 1.73 1.68 1.62 1.61 1.60 1.59 1.58 1.57 1.54 1.53 1.52 1.50 1.49 1.48 1.47 1.35 1.34 1.32 1.32 1.29 1.28 1.26 1.25 1.23 1.22 0.86 0.84 0.82 0.81
Me
Ph
N
O
O
N
H
N
Boc
(2'S)-33
Solvent CDCl3
MHz 500
Nucleus 1H
f1 (ppm)
0.41 0.11 0.26 1.34 0.26 0.49 3.11 0.12 0.43 0.28 0.37 1.05 0.39 0.58 0.95 0.48 0.74 1.38 1.87 1.29 1.18 5.23 4.57 8.68 4.60 3.00

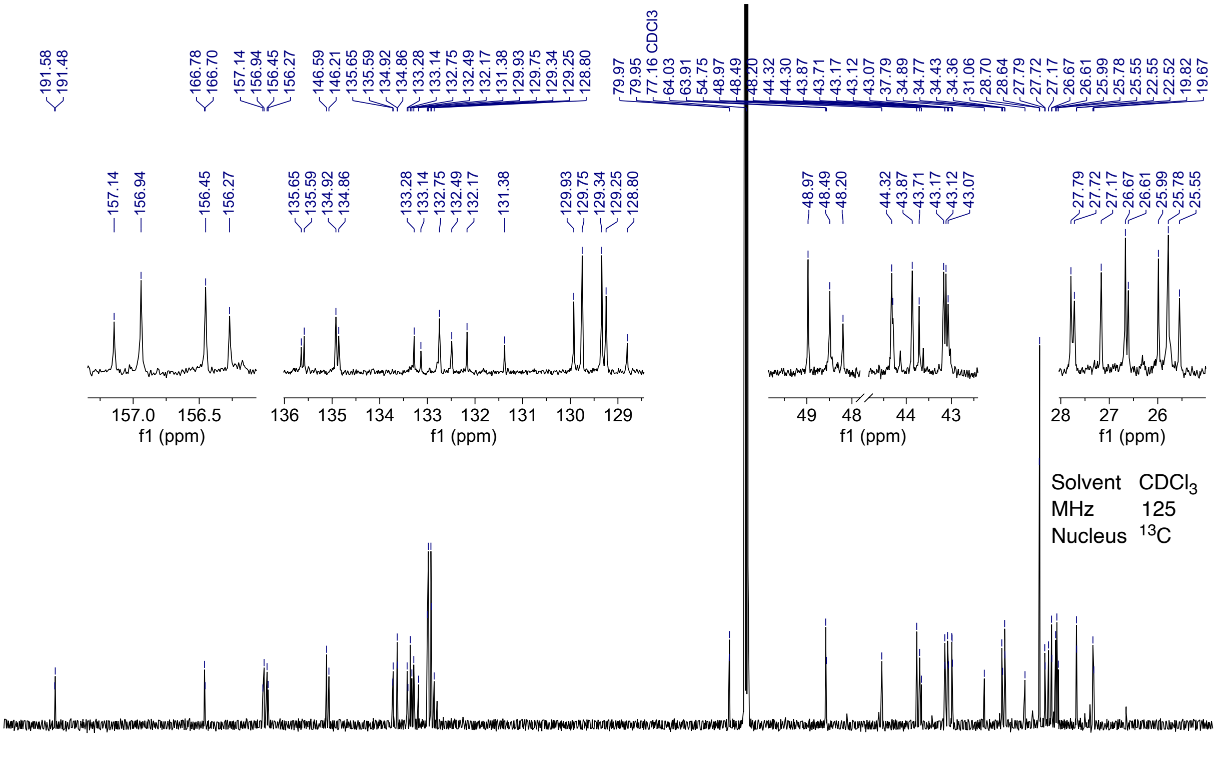
191.58 191.48
166.78 166.70
157.14 156.94 156.45 156.27
146.59 146.21 135.65 135.59 134.92 134.86 133.28 133.14 132.75 132.49 132.17 131.38 129.93 129.75 129.34 129.25 128.80
79.97 79.95
77.16 CDCl3
64.03 63.91 54.75 48.97 48.49 48.20 44.32 44.30 43.87 43.71 43.17 43.12 43.07 37.79 34.89 34.77 34.43 34.36 31.06 28.70 28.64 27.79 27.72 27.17 26.67 26.61 25.99 25.78 25.55 22.55 22.52 19.82 19.67
Solvent CDCl3
MHz 125
Nucleus 13C
f1 (ppm)

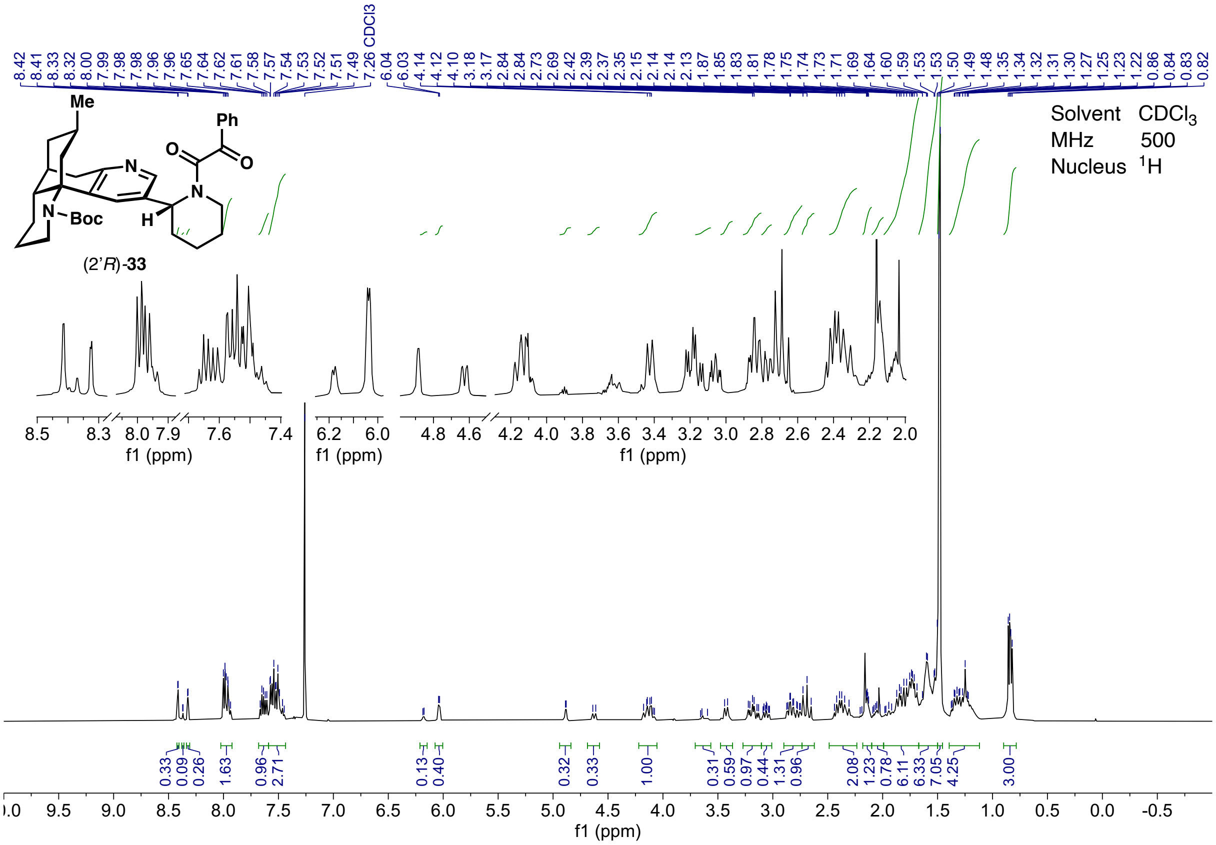
(2'R)-33
Solvent CDCl3
MHz 500
Nucleus 1H
f1 (ppm)

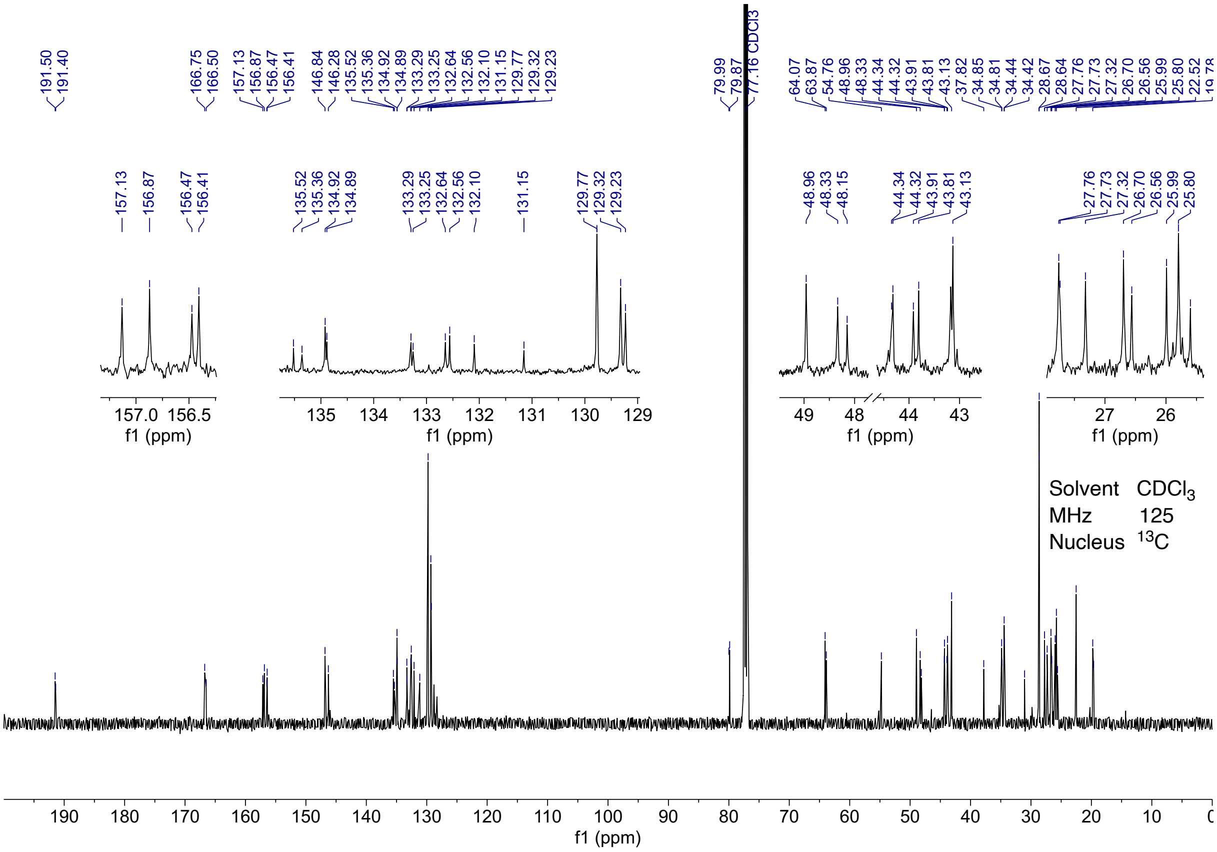
Solvent CDCl3
MHz 125
Nucleus 13C
f1 (ppm)

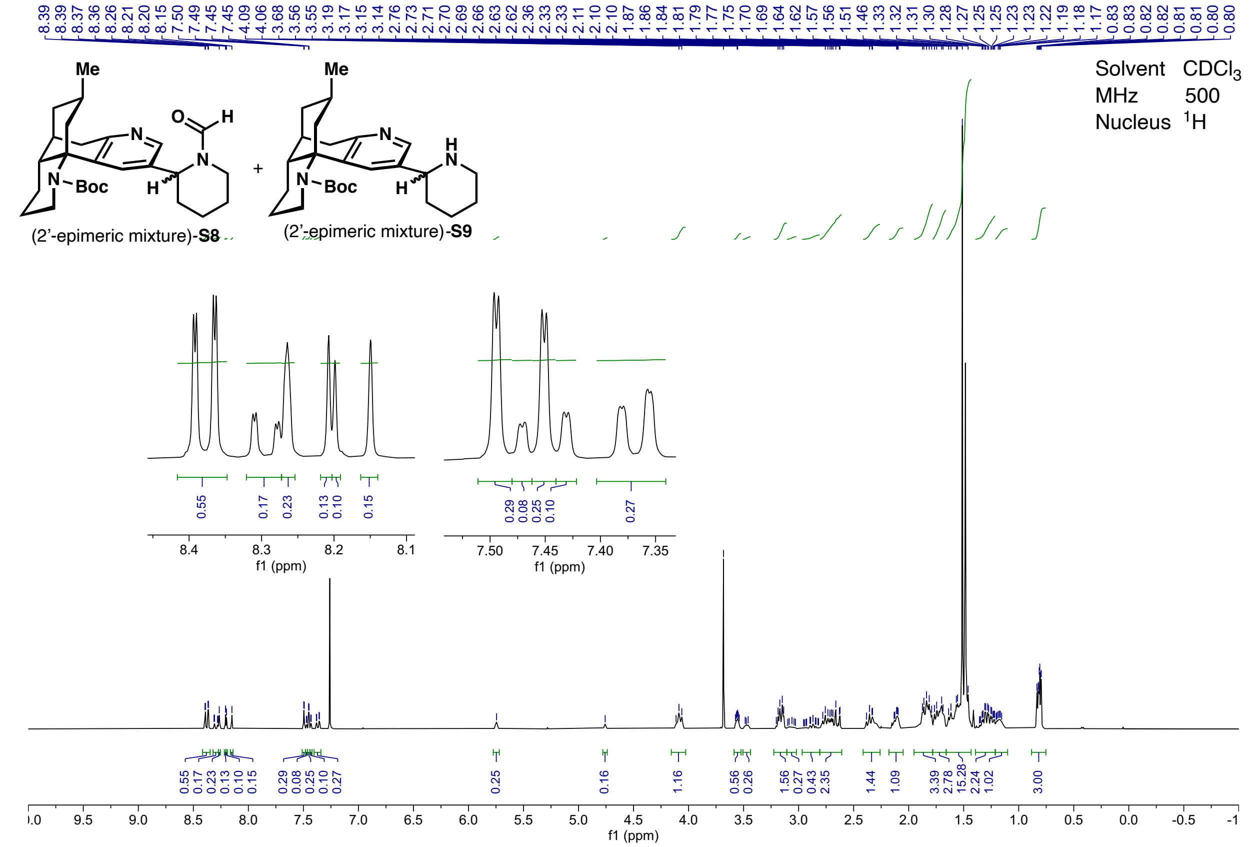
(2'-epimeric mixture)-S8
+
(2'-epimeric mixture)-S9
Me
N
Boc
Solvent CDCl3
MHz 500
Nucleus 1H
f1 (ppm)

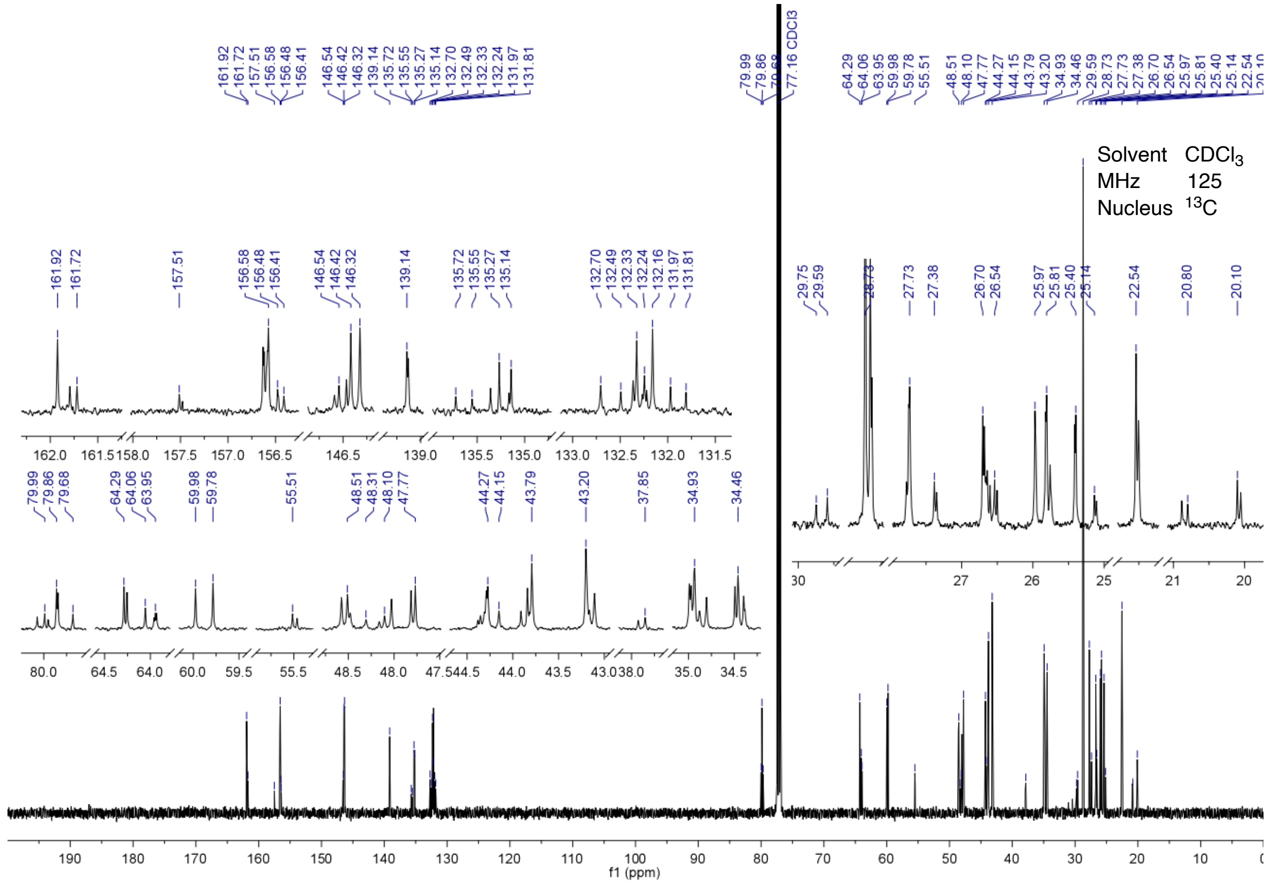
Solvent CDCl3
MHz 125
Nucleus 13C
77.16 CDCl3
f1 (ppm)

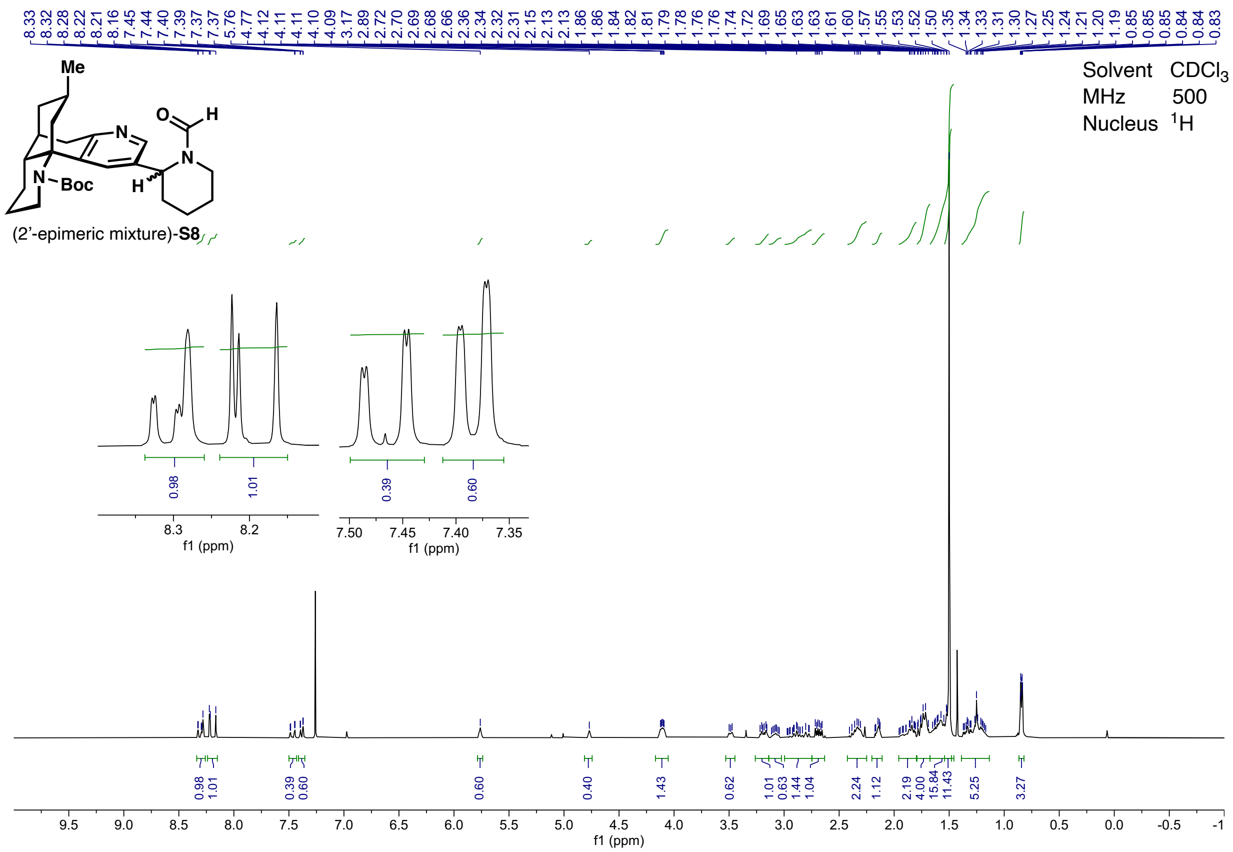
(2'-epimeric mixture)-S8
Solvent CDCl3
MHz 500
Nucleus 1H
f1 (ppm)

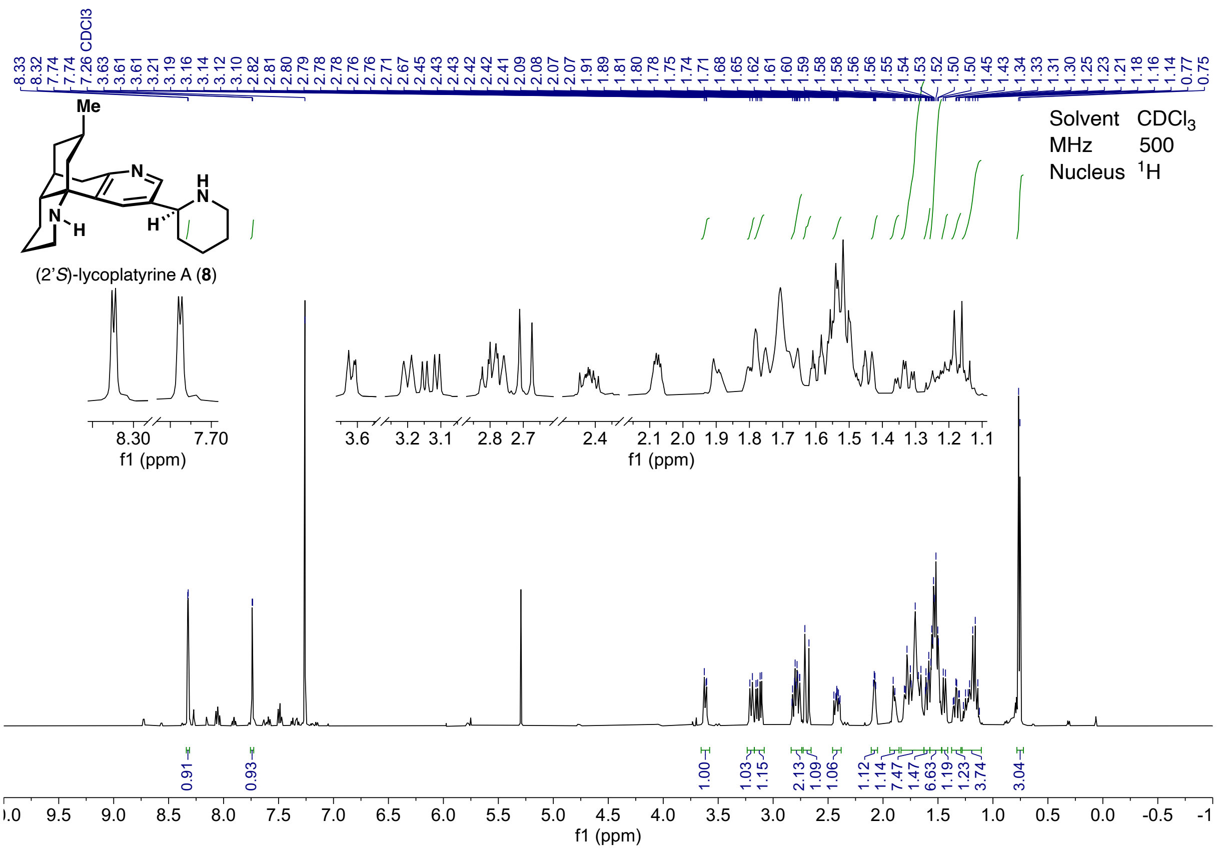
Solvent CDCl3
MHz 500
Nucleus 1H
(2'S)-lycoplatyrine A (8)
f1 (ppm)

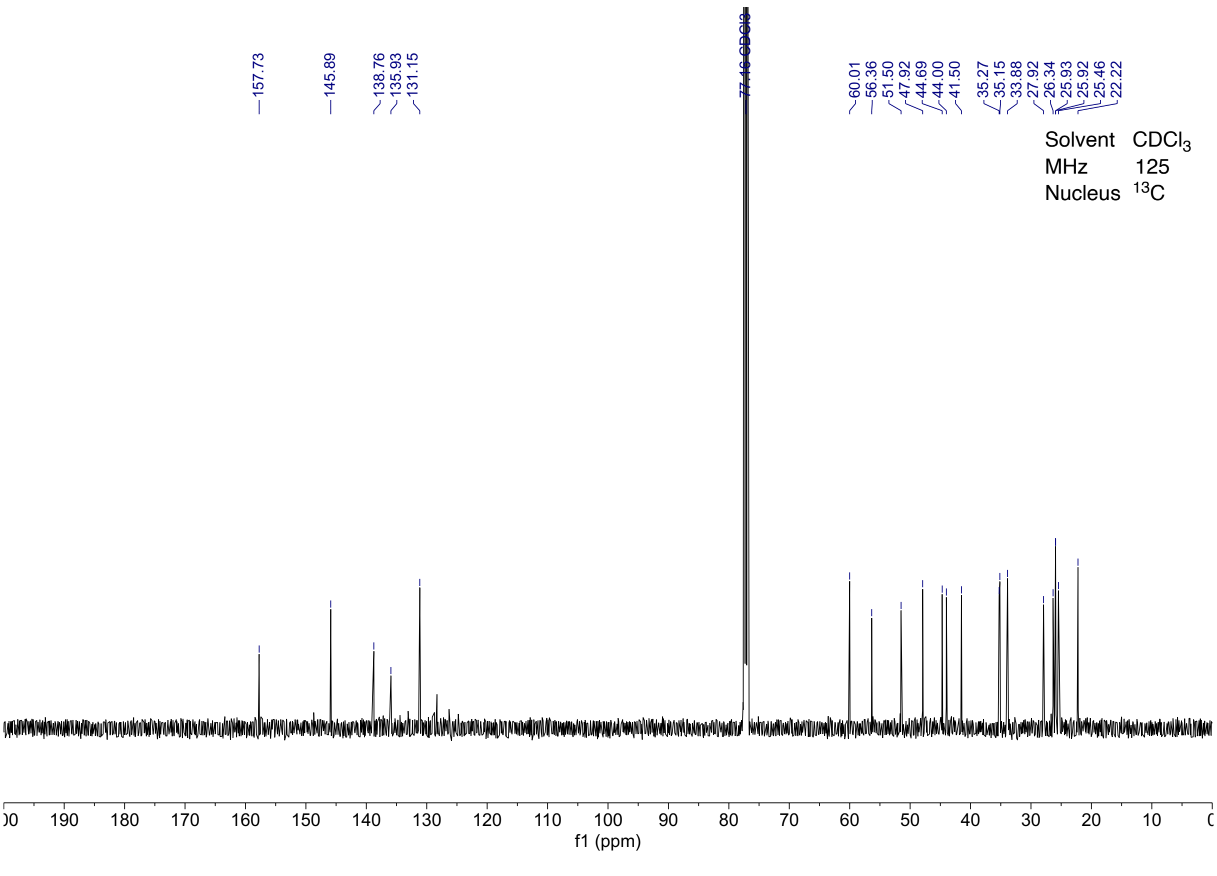
Solvent CDCl3
MHz 125
Nucleus 13C
f1 (ppm)

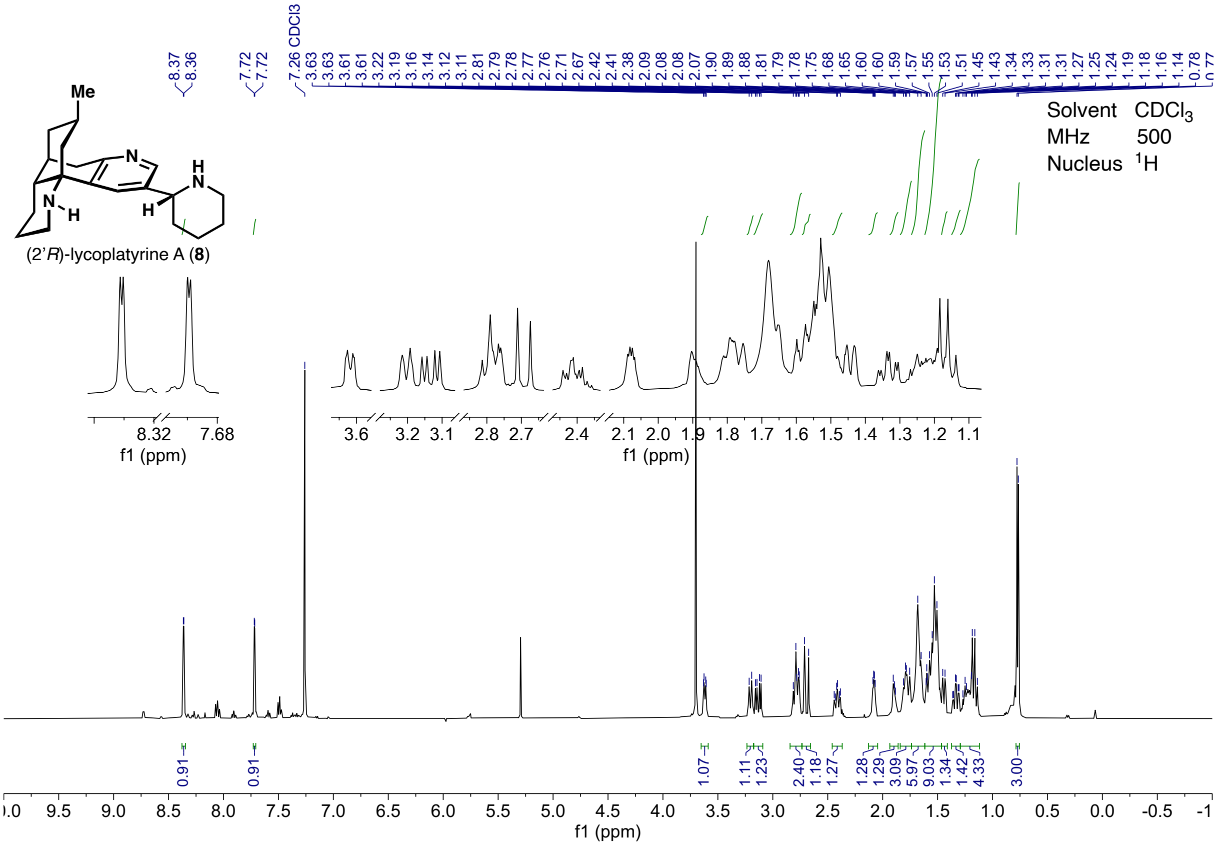
Me
N
H
N
H
H
(2'R)-lycoplatyrine A (8)
Solvent CDCl3
MHz 500
Nucleus 1H
f1 (ppm)

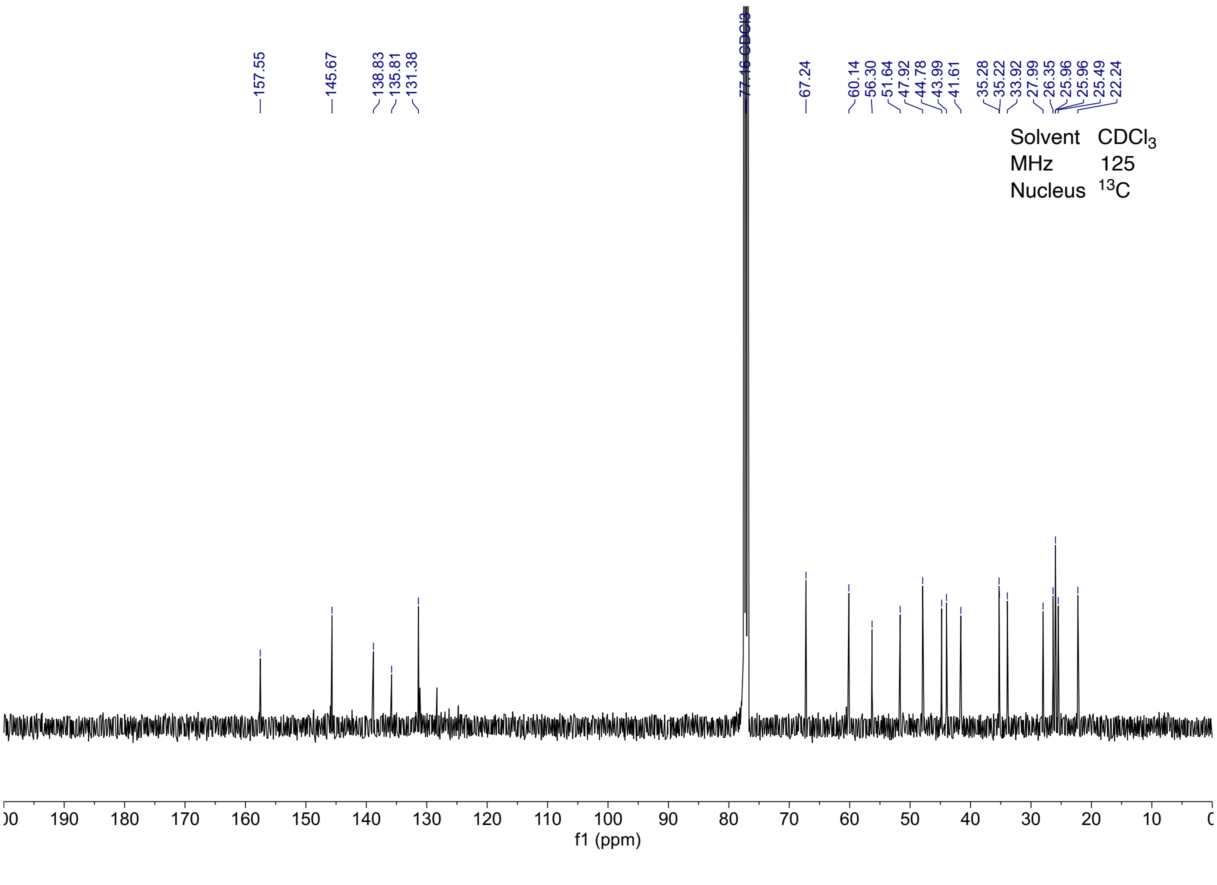
Solvent CDCl3
MHz 125
Nucleus 13C
f1 (ppm)

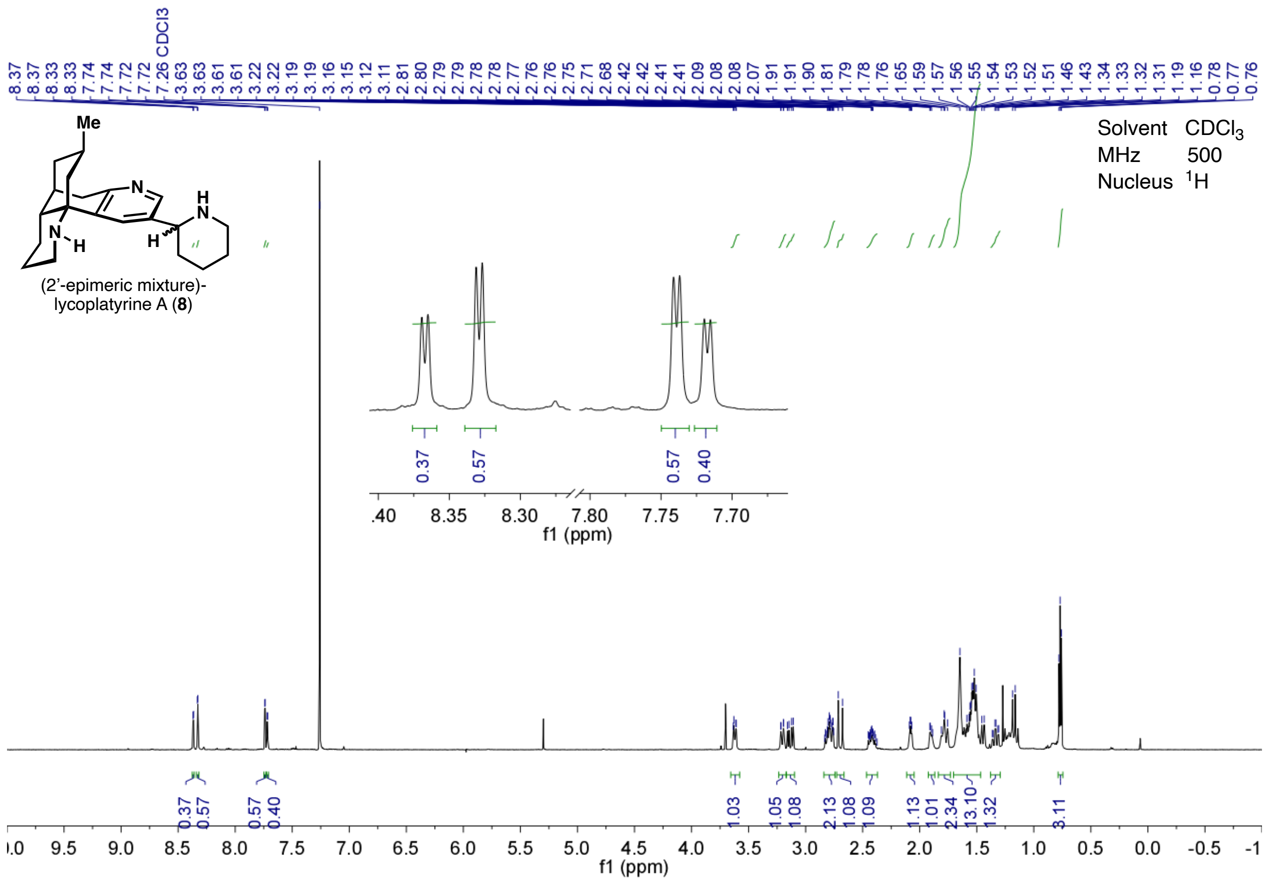
Me
N
H
N
N
H
H
(2'-epimeric mixture)-
lycoplatyrine A (8)
Solvent CDCl3
MHz 500
Nucleus 1H
f1 (ppm)
f1 (ppm)

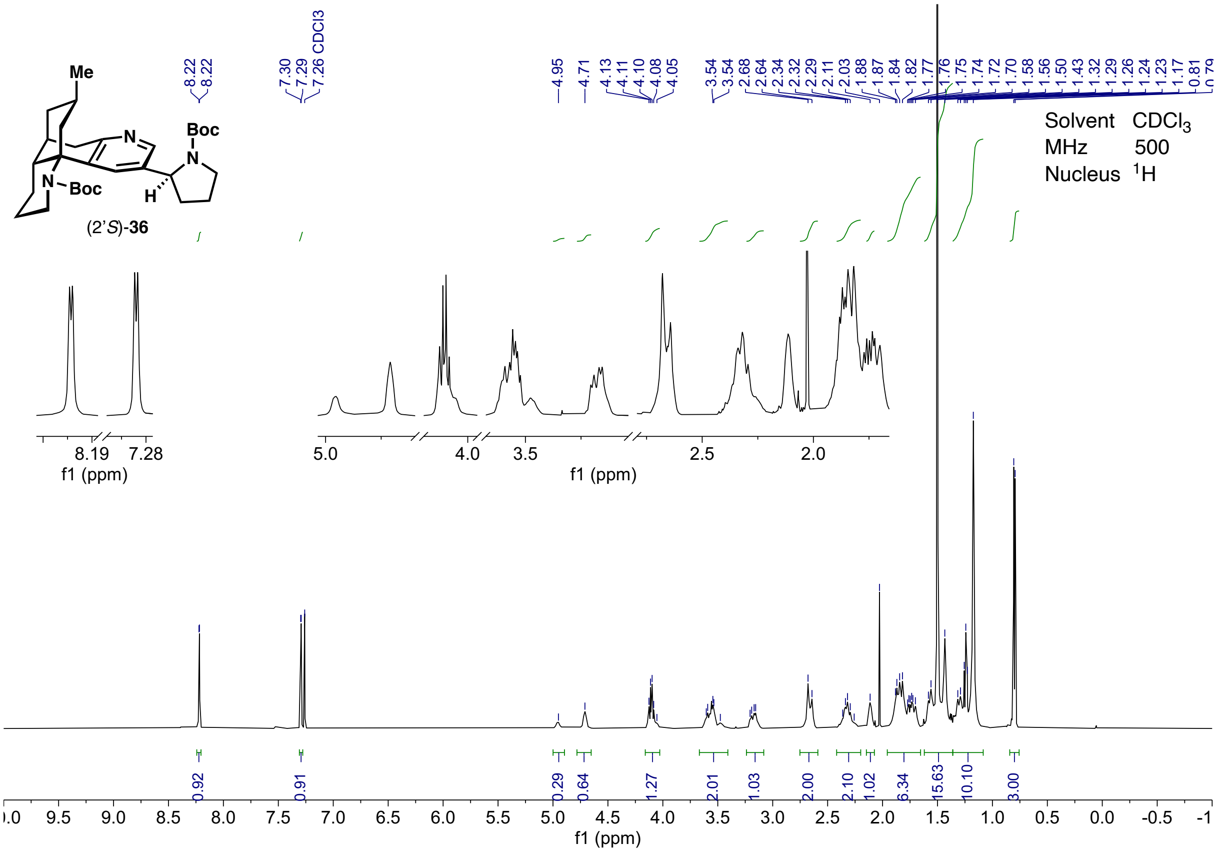
Me
N
Boc
N
H
N
Boc
(2'S)-36
Solvent CDCl3
MHz 500
Nucleus 1H
f1 (ppm)

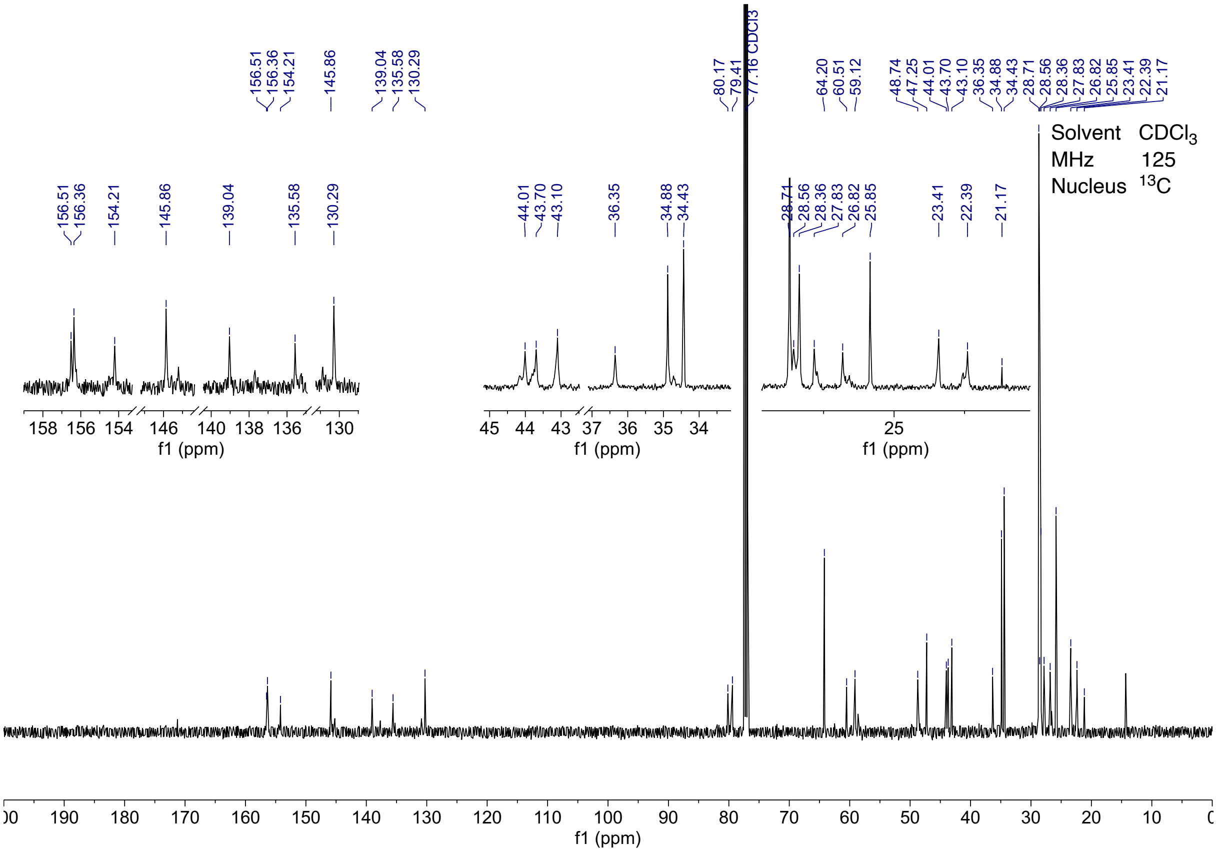
Solvent CDCl3
MHz 125
Nucleus 13C
f1 (ppm)

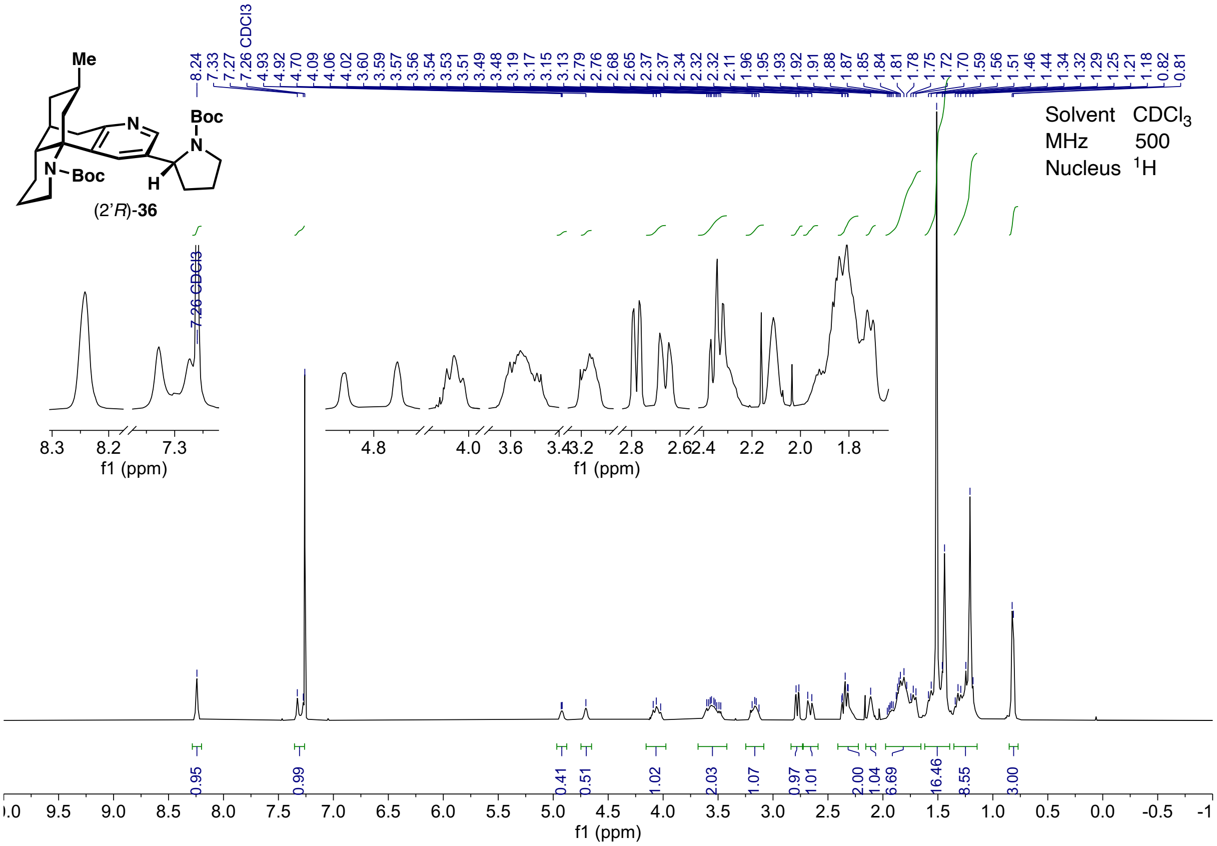
Me
Boc
N
N
Boc
H
(2'R)-36
Solvent CDCl3
MHz 500
Nucleus 1H
f1 (ppm)

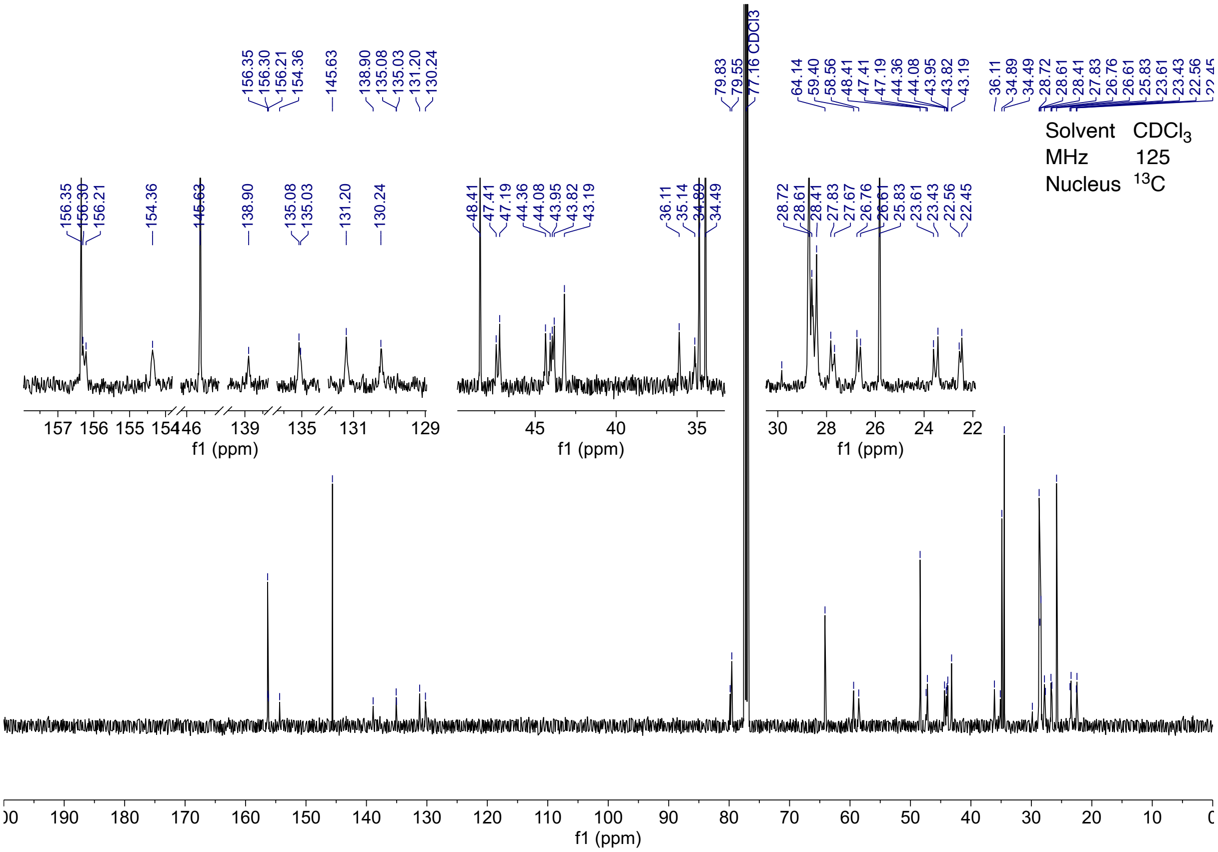
Solvent CDCl3
MHz 125
Nucleus 13C
f1 (ppm)

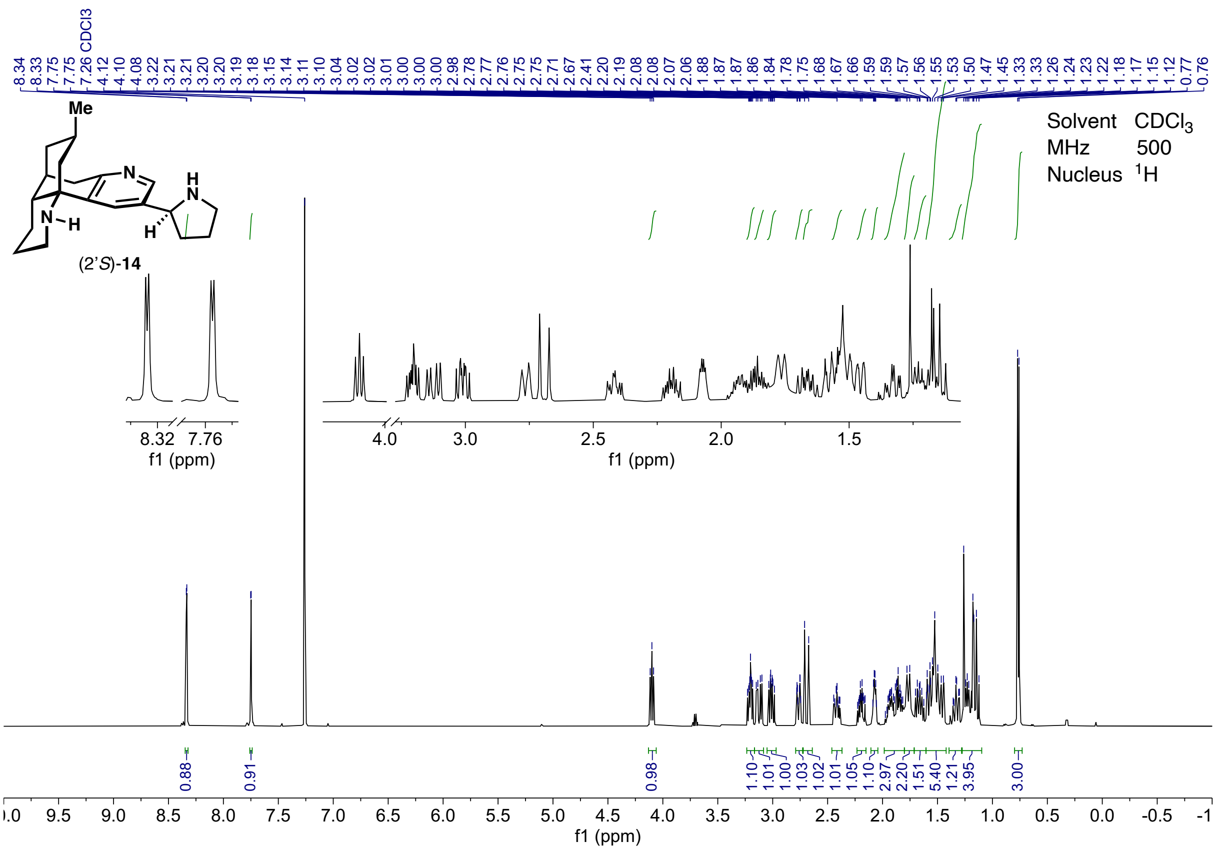

8.34 8.33 7.75 7.26 CDCl3 4.12 4.10 4.08 3.22 3.21 3.21 3.20 3.19 3.18 3.15 3.14 3.11 3.10 3.04 3.02 3.02 3.01 3.00 3.00 2.98 2.78 2.77 2.76 2.75 2.75 2.71 2.67 2.41 2.20 2.19 2.08 2.08 2.07 2.06 1.88 1.87 1.87 1.86 1.84 1.78 1.75 1.68 1.67 1.66 1.59 1.59 1.57 1.56 1.55 1.53 1.50 1.47 1.45 1.33 1.33 1.26 1.24 1.23 1.22 1.18 1.17 1.15 1.12 0.77 0.76
Me
N
H
N
N–H
H
(2'S)-14
Solvent CDCl3
MHz 500
Nucleus 1H
8.32 7.76
f1 (ppm)
4.0 3.0 2.5 2.0 1.5
f1 (ppm)
0.88 0.91 0.98 1.10 1.01 1.00 1.03 1.02 1.01 1.05 1.10 2.97 2.20 1.51 5.40 1.21 3.95 3.00
f1 (ppm)

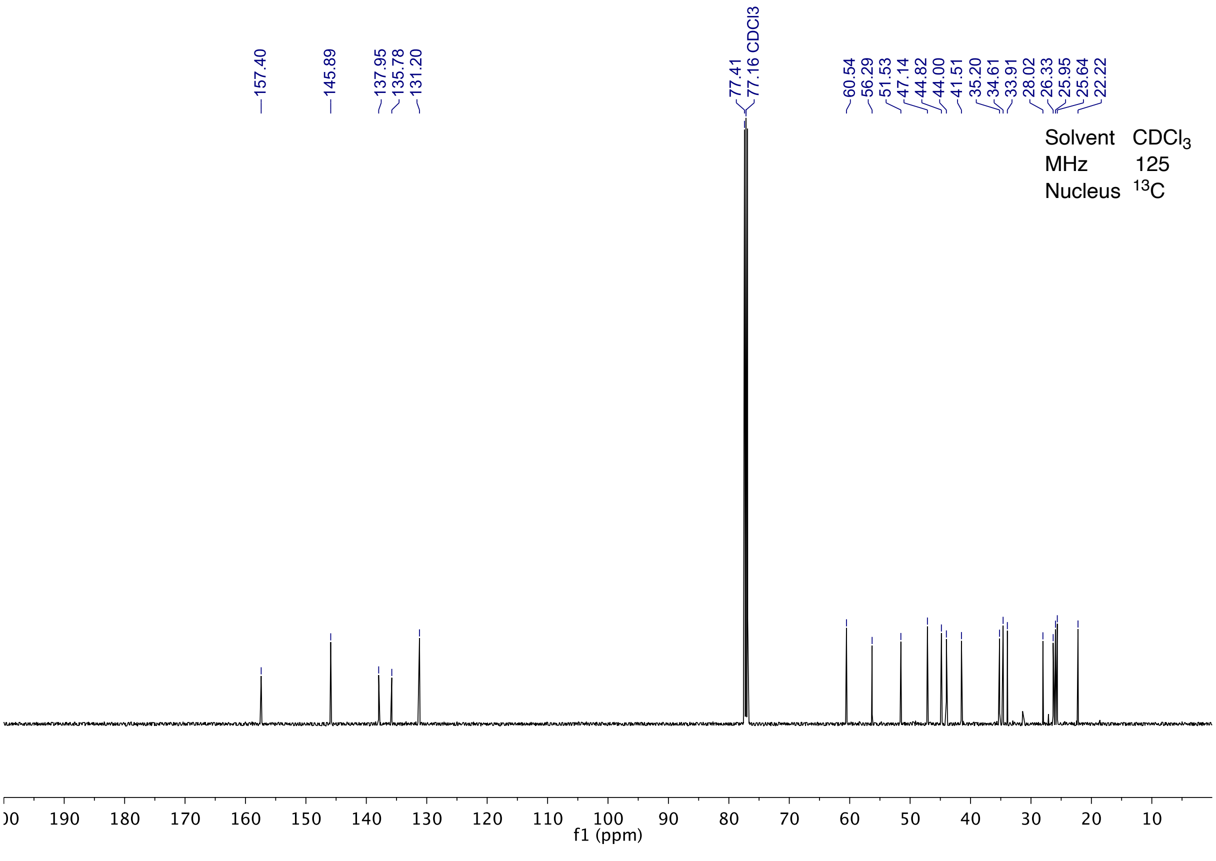

157.40 145.89 137.95 135.78 131.20 77.41 77.16 CDCl3 60.54 56.29 51.53 47.14 44.82 44.00 41.51 35.20 34.61 33.91 28.02 26.33 25.95 25.64 22.22
Solvent CDCl3
MHz 125
Nucleus 13C
f1 (ppm)

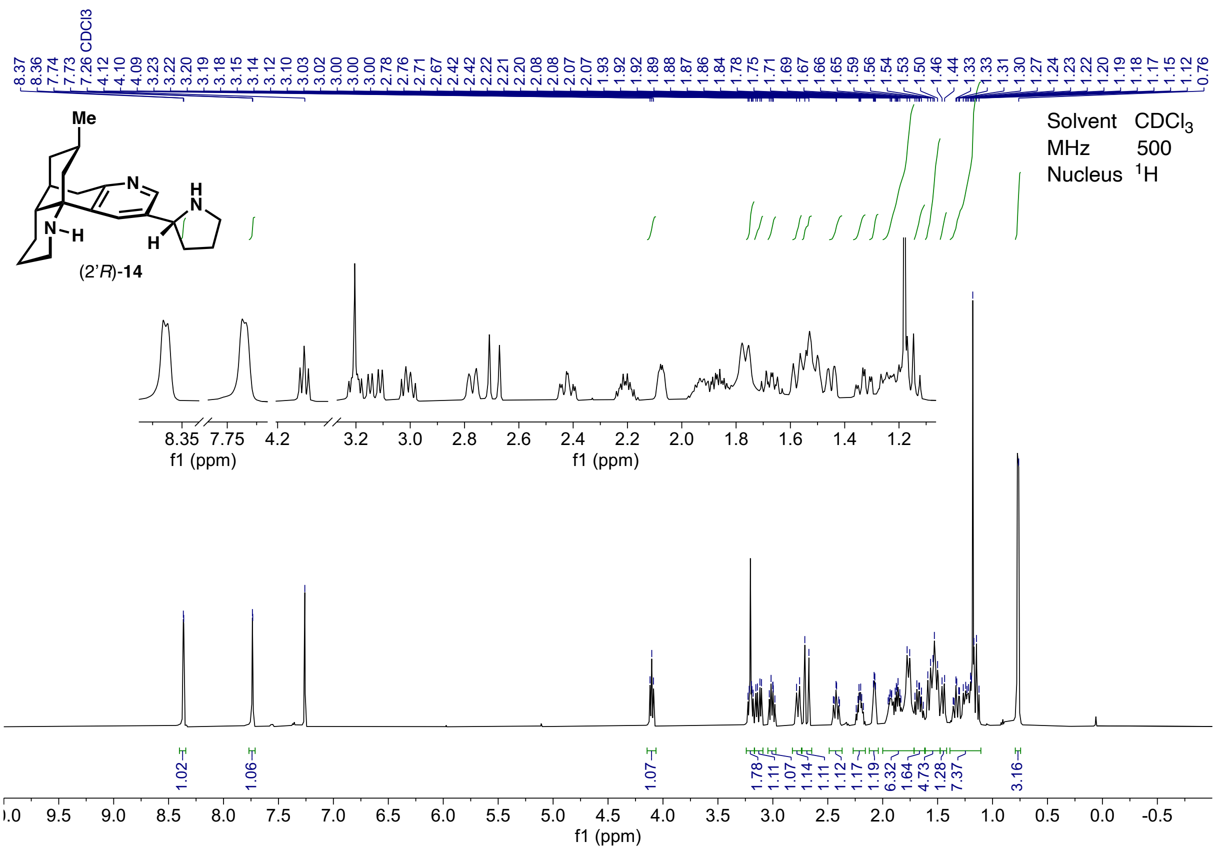
Me
(2'R)-14
Solvent CDCl3
MHz 500
Nucleus 1H
f1 (ppm)

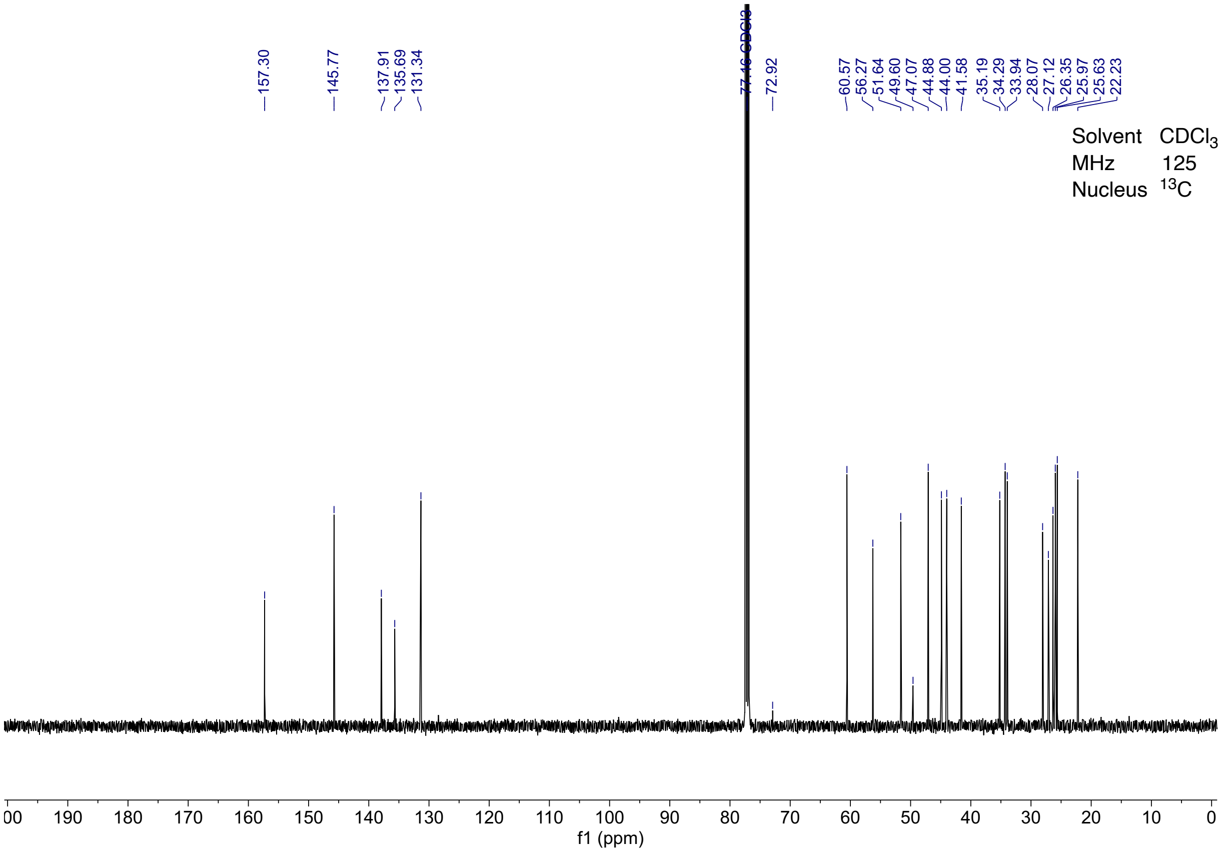
Solvent CDCl3
MHz 125
Nucleus 13C
f1 (ppm)

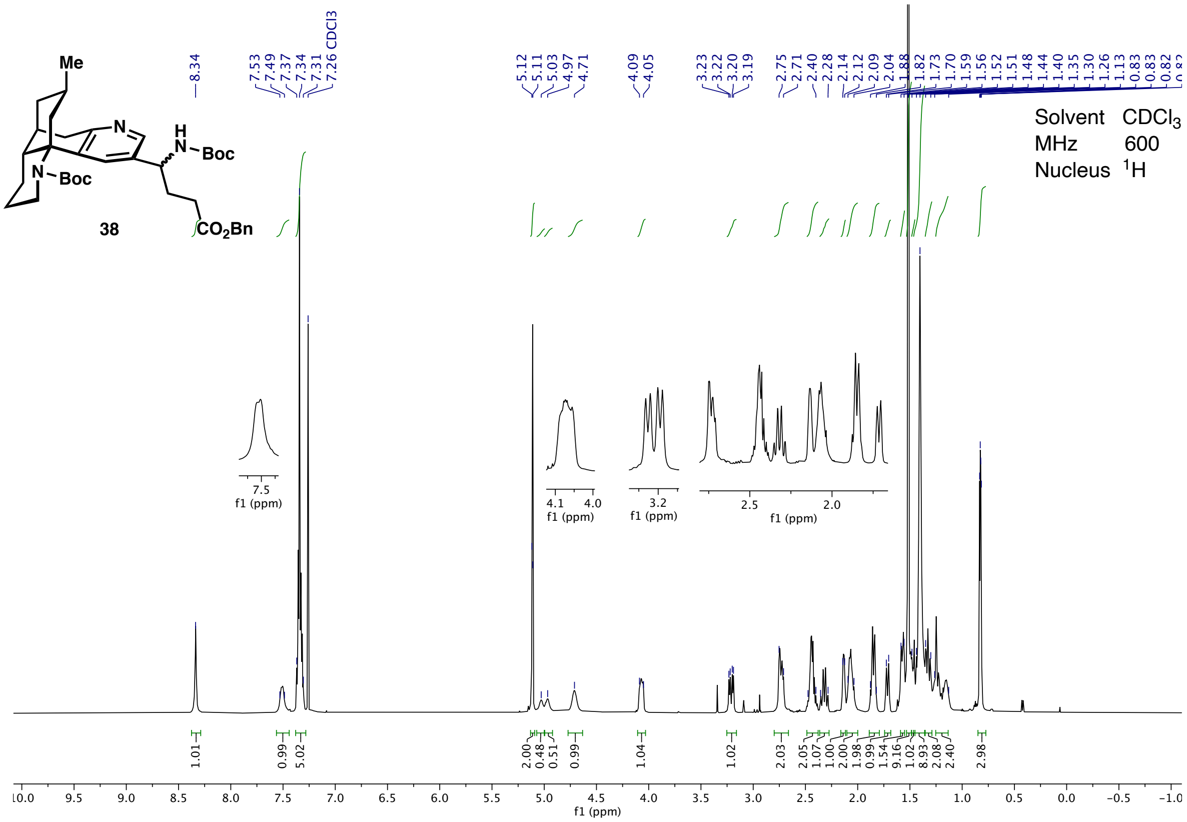
Me
N
H
N
Boc
N
Boc
38
CO2Bn
8.34
7.53 7.49 7.37 7.34 7.31 7.26 CDCl3
5.12 5.11 5.03 4.97 4.71
4.09 4.05
3.23 3.22 3.20 3.19
2.75 2.71 2.40 2.28 2.14 2.12 2.09 2.04 1.88 1.82 1.73 1.70 1.59 1.56 1.52 1.51 1.48 1.44 1.40 1.35 1.30 1.26 1.13 0.83 0.82
Solvent CDCl3
MHz 600
Nucleus 1H
1.01 0.99 5.02 2.00 0.48 0.51 0.99 1.04 1.02 2.03 2.05 1.07 1.00 2.00 1.98 0.99 1.54 9.16 1.02 8.93 2.08 2.40 2.98
f1 (ppm)

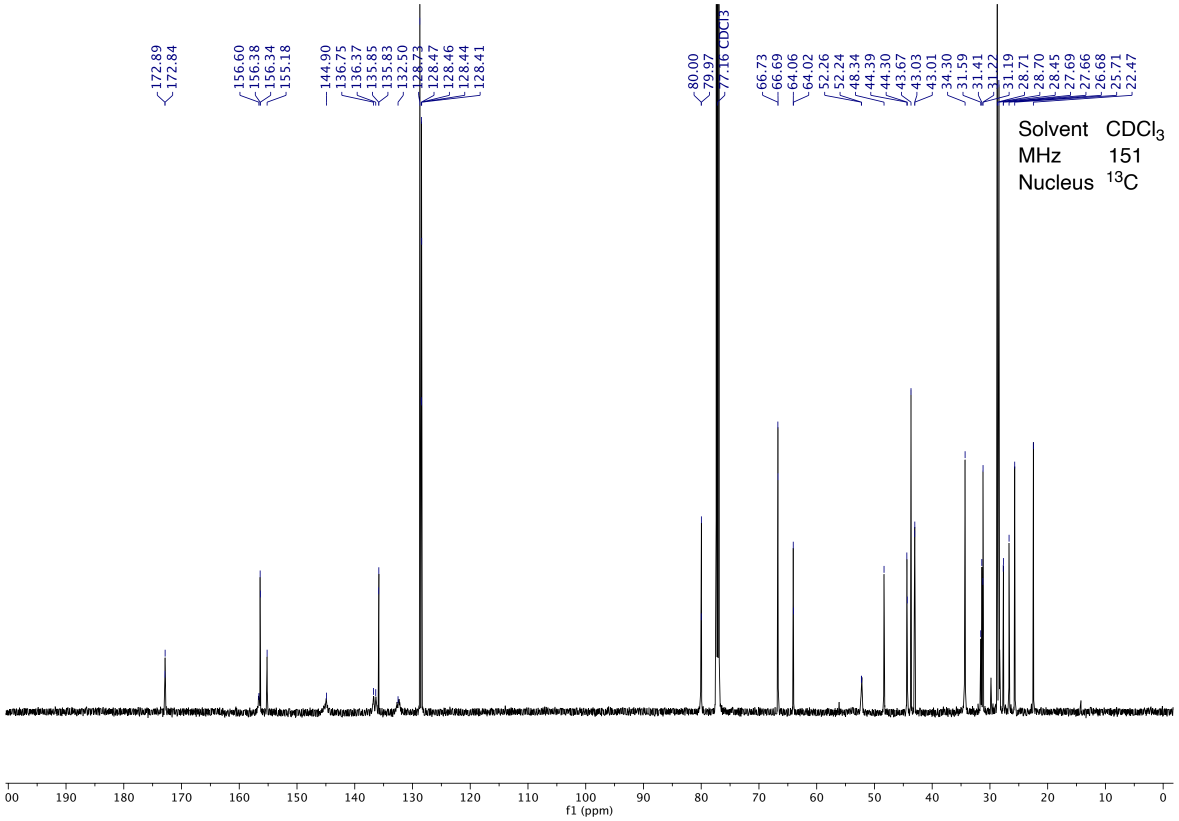
172.89 172.84
156.60 156.38 156.34 155.18
144.90 136.75 136.37 135.85 135.83 132.50 128.73 128.47 128.46 128.44 128.41
80.00 79.97 77.16 CDCl3
66.73 66.69 64.06 64.02 52.26 52.24 48.34 44.39 44.30 43.67 43.03 34.30 31.59 31.41 31.22 31.19 28.71 28.70 28.45 27.69 27.66 26.68 25.71 22.47
Solvent CDCl3
MHz 151
Nucleus 13C
f1 (ppm)

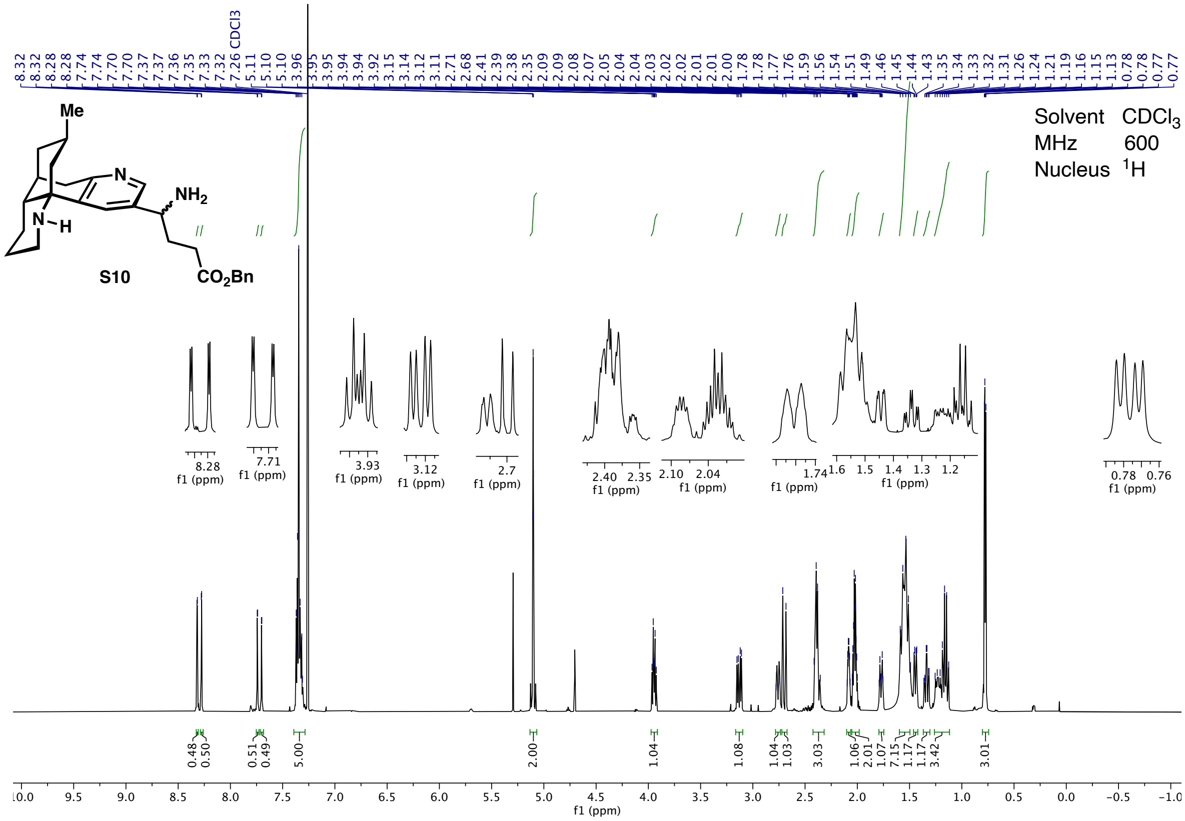
Solvent CDCl3
MHz 600
Nucleus 1H
S10
f1 (ppm)

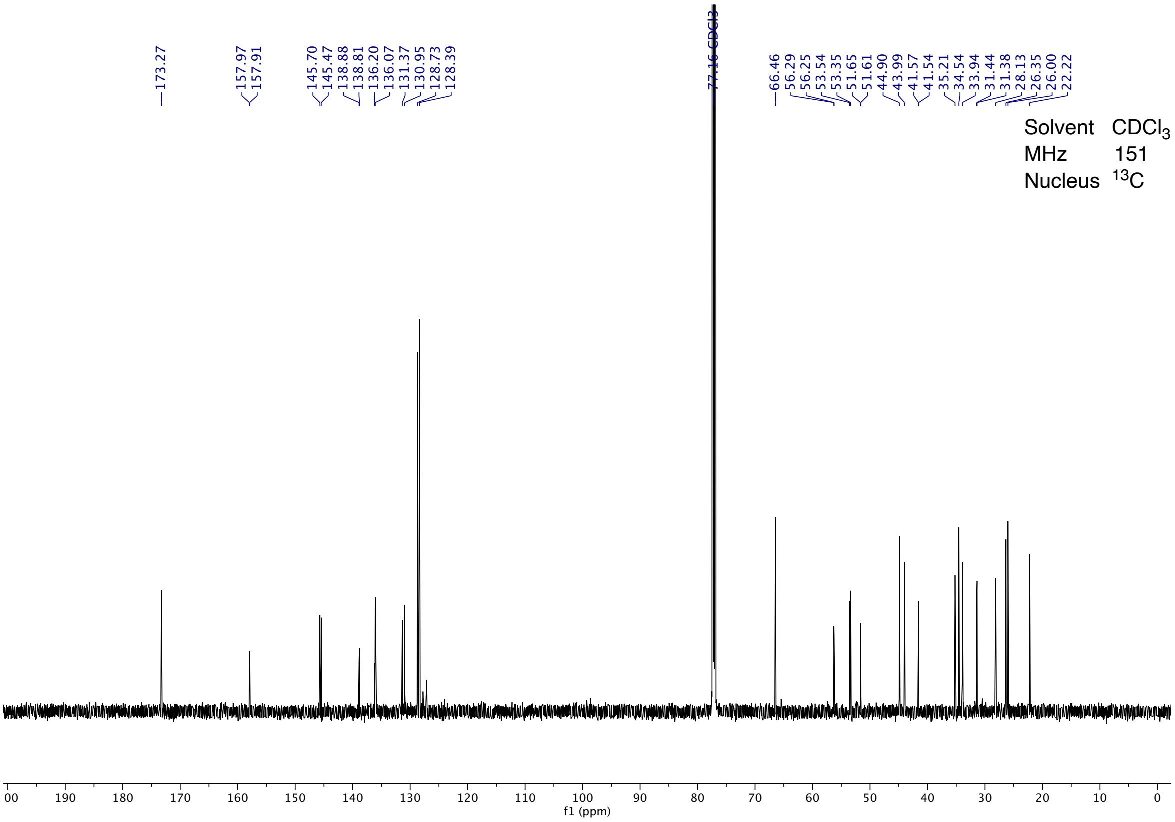
Solvent CDCl3
MHz 151
Nucleus 13C
173.27
157.97
157.91
145.70
145.47
138.88
138.81
136.20
136.07
131.37
130.95
128.73
128.39
77.16 CDCl3
66.46
56.29
56.25
53.54
53.35
51.65
51.61
44.90
43.99
41.57
41.54
35.21
34.54
33.94
31.44
31.38
28.13
26.35
26.00
22.22
f1 (ppm)

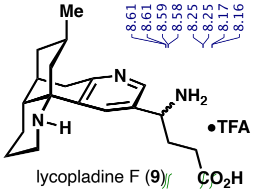

lycopladine F (9)

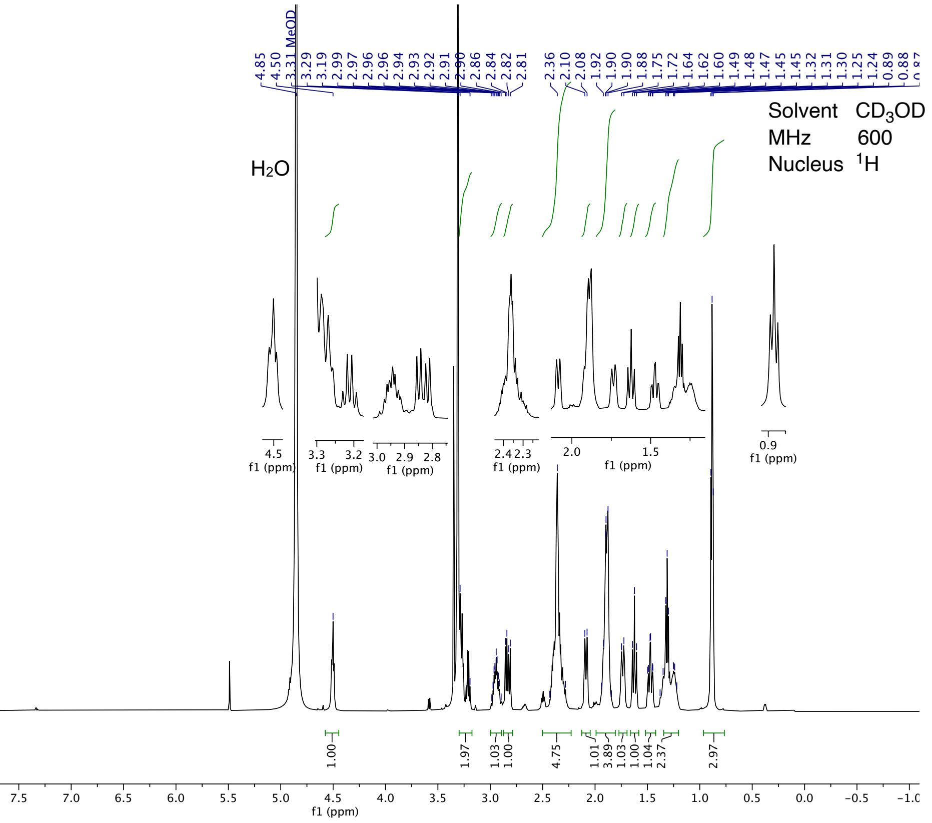

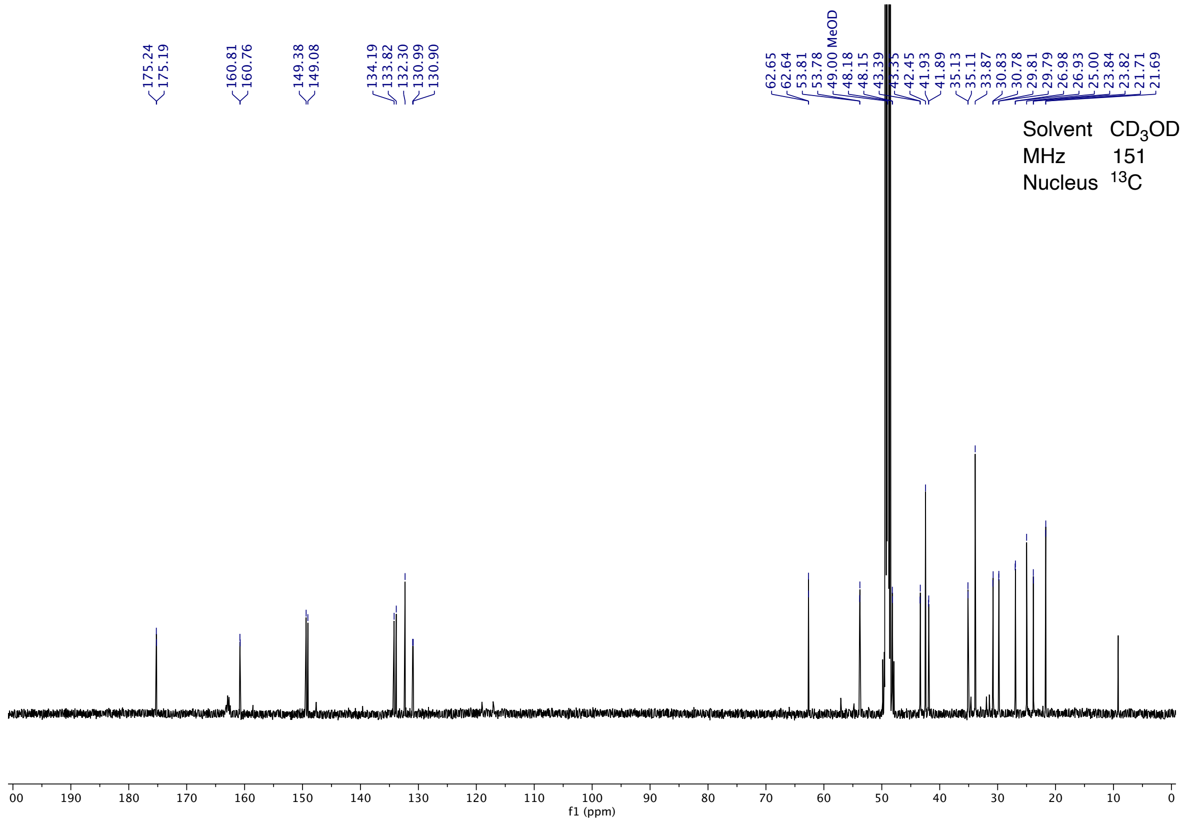

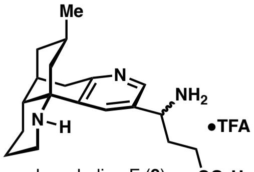

lycopladine F (**9**)

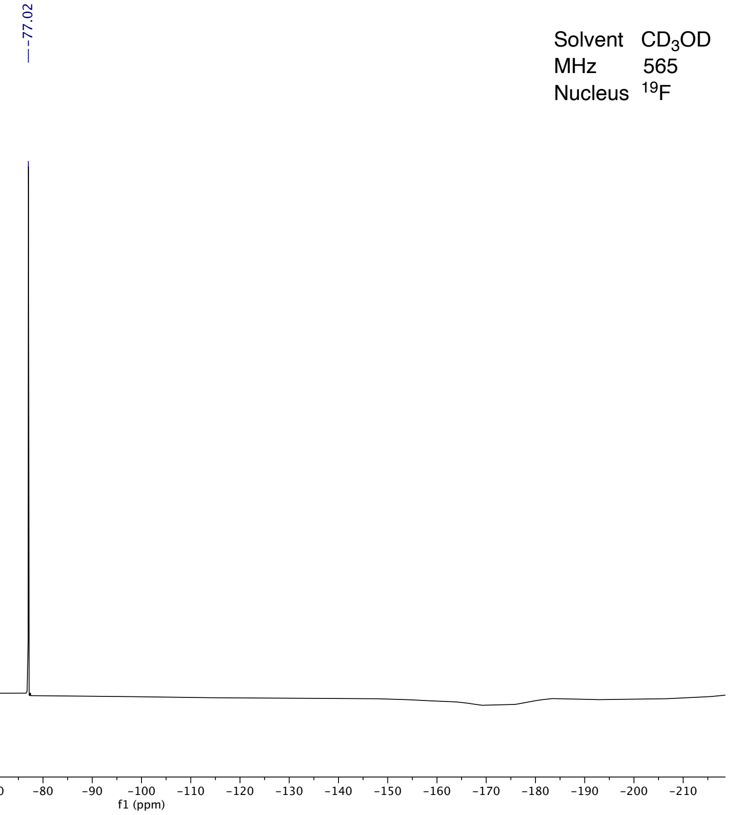

Solvent CD_3OD
MHz 565
Nucleus ^{19}F

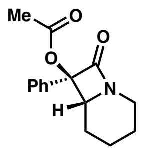

rac-**S13**

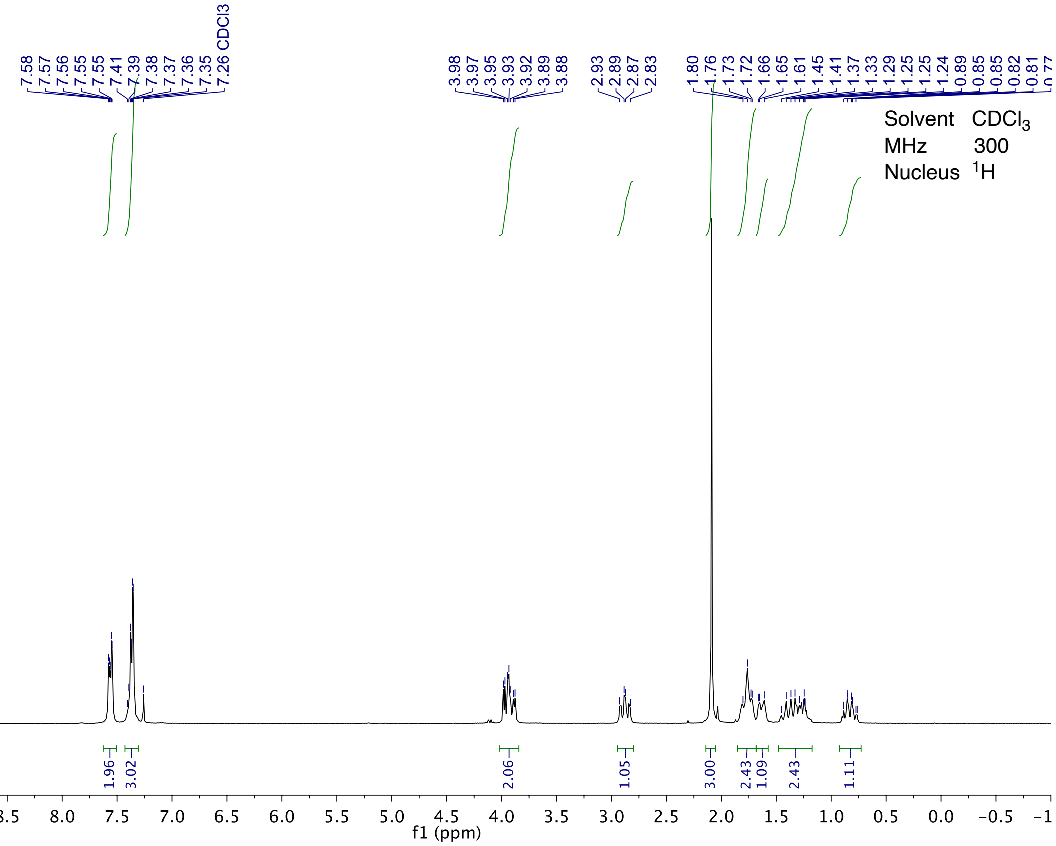
7.58
7.57
7.56
7.55
7.55
7.41
7.39
7.38
7.37
7.36
7.35
7.26 CDCl3
3.98
3.97
3.95
3.93
3.92
3.89
3.88
2.93
2.89
2.87
2.83
1.80
1.76
1.73
1.72
1.66
1.65
1.61
1.45
1.41
1.37
1.33
1.29
1.25
1.25
1.24
0.89
0.85
0.85
0.82
0.81
Solvent CDCl3
MHz 300
Nucleus 1H
1.96
3.02
2.06
1.05
3.00
2.43
1.09
2.43
1.11
f1 (ppm)

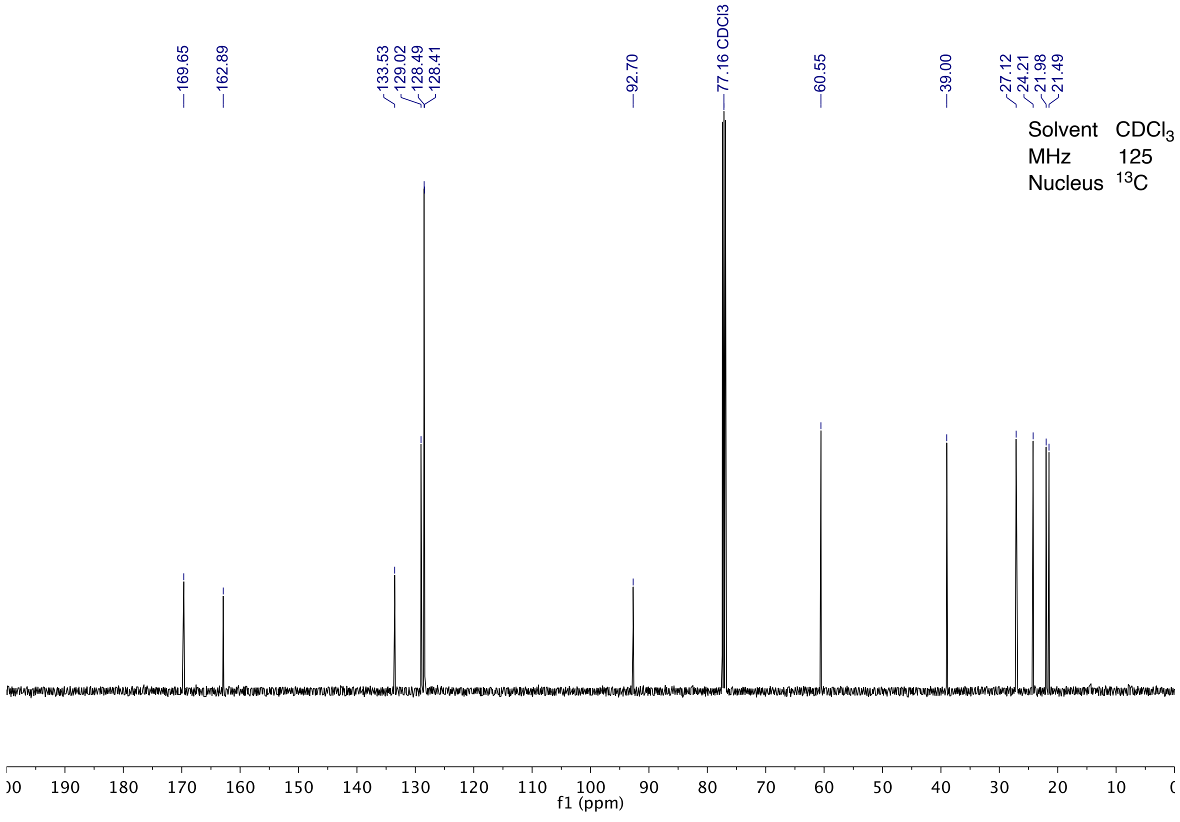
169.65
162.89
133.53
129.02
128.49
128.41
92.70
77.16 CDCl3
60.55
39.00
27.12
24.21
21.98
21.49
Solvent CDCl3
MHz 125
Nucleus 13C
f1 (ppm)

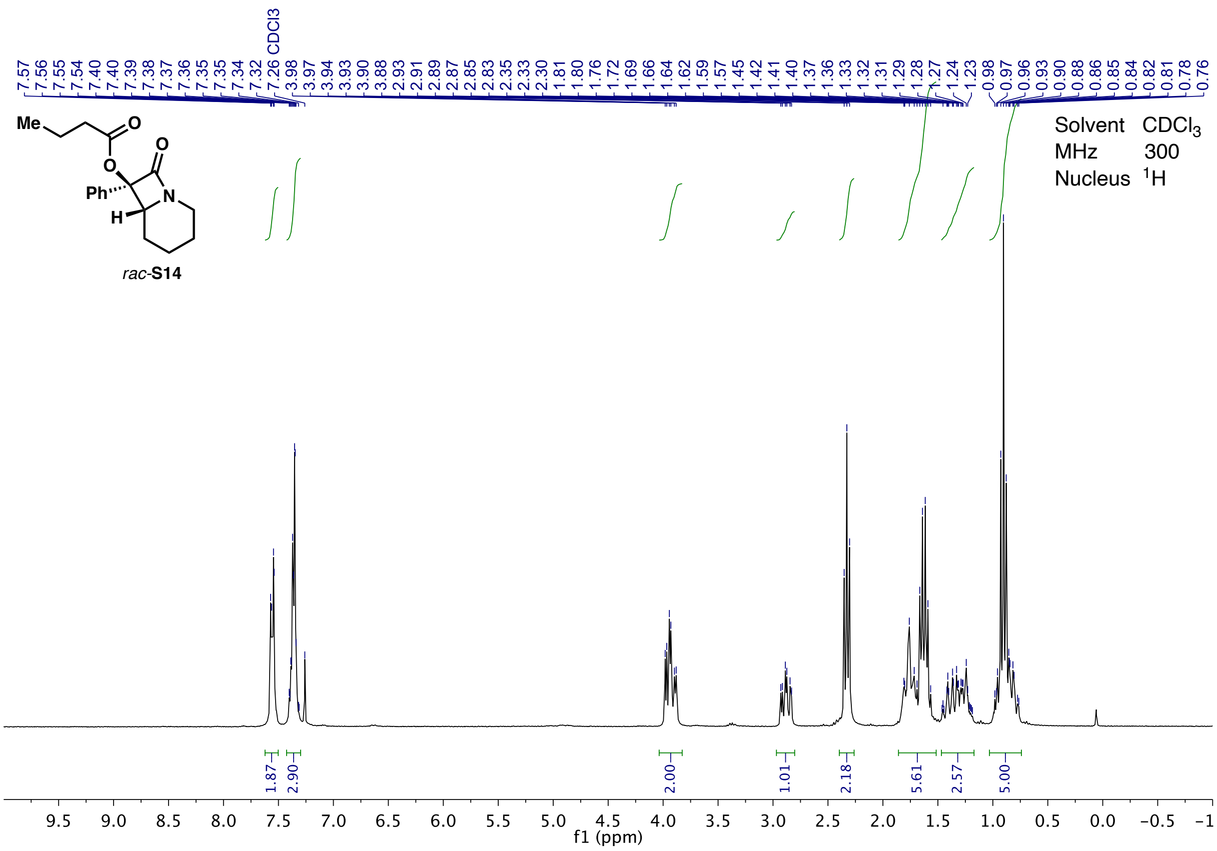
7.57 7.56 7.55 7.54 7.40 7.40 7.39 7.38 7.37 7.36 7.35 7.35 7.34 7.32 7.26 CDCl3 3.98 3.97 3.94 3.93 3.90 3.88 2.93 2.91 2.89 2.87 2.85 2.83 2.35 2.33 2.30 1.81 1.80 1.76 1.72 1.69 1.66 1.64 1.62 1.59 1.57 1.45 1.42 1.41 1.40 1.37 1.36 1.33 1.32 1.31 1.29 1.28 1.27 1.24 1.23 0.98 0.97 0.96 0.93 0.90 0.88 0.86 0.85 0.84 0.82 0.81 0.78 0.76
Me
O
O
O
Ph
H
N
rac-S14
Solvent CDCl3
MHz 300
Nucleus 1H
1.87 2.90 2.00 1.01 2.18 5.61 2.57 5.00
9.5 9.0 8.5 8.0 7.5 7.0 6.5 6.0 5.5 5.0 4.5 4.0 3.5 3.0 2.5 2.0 1.5 1.0 0.5 0.0 −0.5 −1
f1 (ppm)

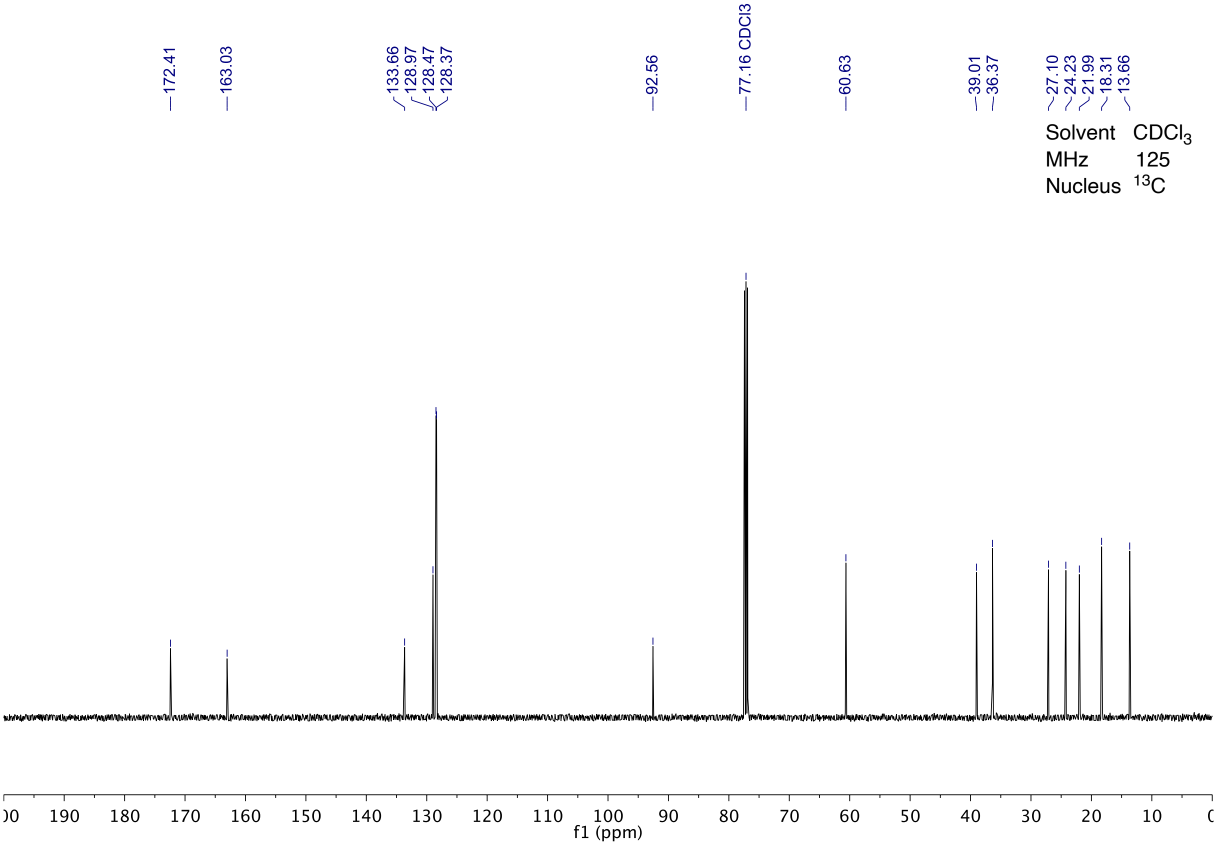
172.41 163.03 133.66 128.97 128.47 128.37 92.56 77.16 CDCl3 60.63 39.01 36.37 27.10 24.23 21.99 18.31 13.66
Solvent CDCl3
MHz 125
Nucleus 13C
190 180 170 160 150 140 130 120 110 100 90 80 70 60 50 40 30 20 10
f1 (ppm)
